# Design, Properties and Applications of Multiple Dynamic Hydrogels

**DOI:** 10.3390/ma19163458

**Published:** 2026-08-14

**Authors:** Haofei Yang, Silu Wang, Shehzadi Mehboob, Jianhua Zhang

**Affiliations:** 1 Key Laboratory of Systems Bioengineering of the Ministry of Education, Department of Polymer Science and Engineering, School of Chemical Engineering and Technology, Tianjin University, Tianjin 300350, China; yang_ha0fei@tju.edu.cn (H.Y.); wangsilu@tju.edu.cn (S.W.); shehzadimehboob53@gmail.com (S.M.); 2 Tianjin Key Laboratory of Membrane Science and Desalination Technology, Tianjin University, Tianjin 300350, China

**Keywords:** hydrogels, dynamic covalent bonds, non-covalent interactions, multiple crosslinking, self-healing, multifunctional materials

## Abstract

**Highlights:**

- Systematically summarizing the advances in multiple dynamic hydrogels by synergistic integration of dynamic covalent and non-covalent bonds.
- Focusing on the design principles—kinetic matching, orthogonal responsiveness, and structure–function integration.
- Highlighting the current challenges and future directions of design, functionalization and applications of multiple dynamic hydrogels.

**Abstract:**

Hydrogels are highly hydrated polymeric soft materials that closely mimic the mechanical characteristics of biological soft tissues, offering immense promise for biomedical applications such as tissue engineering, drug delivery, flexible electronics, and wound repair. Conventional hydrogels crosslinked by static covalent bonds suffer from irreversible network disruption upon mechanical damage and lack active responsiveness to environmental stimuli, severely limiting their practical use. Incorporating both dynamic covalent bonds and dynamic non-covalent interactions endows hydrogels with intelligent features, including self-healing, injectability, and stimuli responsiveness. A single dynamic crosslinking mode often fails to achieve an optimal balance between mechanical robustness and rapid dynamic reversibility, whereas the synergistic integration of multiple dynamic bonds—exploiting their complementarity in kinetics, energy dissipation, and stimuli-responsiveness—has emerged as a cutting-edge strategy to overcome this limitation. Using dynamic non-covalent interactions as the classification framework, this review systematically summarizes recent advances in hydrogels crosslinked by combinations of dynamic non-covalent interactions with dynamic covalent bonds, covering hydrogen bonds, host–guest interactions, metal–ligand interactions, electrostatic interactions, hydrophobic interactions, π–π stacking interactions, and other emerging multiple dynamic crosslinking systems. The design principles, synergistic mechanisms, multifunctional applications, and key challenges and future directions of each system are discussed and prospected.

## 1. Introduction

Hydrogels are three-dimensional network materials formed by the crosslinking of hydrophilic polymer chains through physical or chemical interactions, with water content typically exceeding 90%. Owing to their mechanical properties that closely resemble those of biological soft tissues and their excellent biocompatibility, hydrogels have been widely applied in tissue engineering, drug delivery, wound dressings, flexible sensors, and related fields [1,2,3,4,5]. However, conventional hydrogels are generally crosslinked by irreversible static covalent bonds. Once subjected to mechanical damage, such as tearing or puncturing, the network structure undergoes permanent destruction, leading to irreversible deterioration of critical functions, including mechanical strength and electrical conductivity. Moreover, static networks lack dynamic responsiveness to environmental stimuli such as temperature, pH, and redox conditions, making it difficult to meet the demands of complex physiological environments. Consequently, the development of novel hydrogel systems that combine high mechanical strength with dynamic adaptive capabilities has become a central research direction in this field.

The introduction of dynamic bonds provides a fundamental strategy to address these challenges. Based on their bonding nature, dynamic bonds can be divided into two categories: dynamic covalent bonds (DCBs) and non-covalent interactions [6]. Common dynamic covalent bonds include Schiff base (i.e., imine) bonds, boronate ester bonds, disulfide bonds, acylhydrazone bonds, and Diels–Alder adducts [7,8,9,10,11]. Dynamic covalent bonds possess both the stability and bond-energy characteristics of covalent bonds and the ability to undergo reversible cleavage and reformation under specific stimuli, such as pH changes, temperature, or light, thereby imparting intelligent features, including self-healing, injectability, and stimuli-responsiveness, to polymeric materials [12,13,14]. Non-covalent interactions encompass hydrogen bonds, electrostatic interactions, host–guest interactions, metal–ligand interactions, hydrophobic interactions, π–π stacking, and others [15,16,17,18,19,20,21]. Compared with dynamic covalent bonds, non-covalent interactions exhibit faster exchange kinetics and lower reorganization energy barriers, enabling efficient energy dissipation and self-healing at room temperature [22,23].

Nevertheless, a single type of dynamic bond inevitably suffers from inherent limitations. Hydrogels crosslinked solely by dynamic covalent bonds possess relatively high network stability; yet, their slow bond-exchange kinetics typically necessitate external stimuli, such as heating or pH adjustment, to trigger self-healing. Hydrogels crosslinked exclusively by non-covalent interactions exhibit rapid self-healing but insufficient mechanical strength, falling short of the requirements for load-bearing applications. For instance, temperature-responsive hydrogels represent a prominent class of smart materials that exploit reversible sol–gel transitions, and recent reviews have comprehensively summarized the molecular engineering and dynamic cross-linking strategies employed to optimize their therapeutic applications [24]. Therefore, the strategy of combining different types of dynamic bonds has emerged as a core paradigm in the design of multifunctional hydrogels in recent years. Inspired by the synergistic interplay of multiple weak interactions—such as hydrogen bonds, coordination bonds, and hydrophobic interactions—found in natural biomaterials (e.g., bone, spider silk, and mussel byssus), researchers have extensively explored integrating distinct dynamic bonds within a single hydrogel network. By exploiting their kinetic differences and the complementarity of their stimuli-triggered responses, these bonds can be assigned distinct functional roles. These include network skeleton maintenance, energy dissipation, rapid self-healing, and multiple environmental responsiveness, thereby achieving the synergistic optimization of mechanical strength, toughness, self-healing efficiency, and multi-stimuli responsiveness [25,26,27,28,29,30,31].

The practical utility of multiple dynamic bond-crosslinked hydrogels is inherently linked to their application scenarios, and different bond combinations offer distinct advantages for specific functions. Hydrogen bond/DCB systems, owing to their rapid reversibility and ubiquitous design basis, are particularly suited for high-stretchability sensors and self-healing coatings where rapid room-temperature recovery is essential. Metal–ligand/DCB systems provide tunable bond energies and metal-specific bioactivities, making them preferred candidates for high-strength structural hydrogels, antibacterial wound dressings, and bioactive ion-releasing scaffolds. Host–guest/DCB systems offer molecular-level precision and orthogonal responsiveness, ideally positioned for programmable drug delivery and logic-gated release systems. Electrostatic/DCB systems, with their inherent ionic conductivity and salt/pH sensitivity, excel in flexible electronics, ionic skins, and gastrointestinal drug delivery. Hydrophobic/DCB systems leverage temperature-strengthening effects and hydrophobic drug loading capacity for shape-memory materials and sustained-release depots. π–π stacking/DCB systems enable ordered microstructure formation and photothermal conversion, finding applications in tissue engineering scaffolds and remotely triggered drug release. This application-oriented perspective provides readers with a practical framework for selecting appropriate dynamic bond combinations based on specific functional requirements.

While several excellent reviews have summarized the design and applications of dynamic hydrogels, most have focused either on a single type of dynamic bond or on specific application contexts without systematically addressing the synergistic integration of multiple dynamic bonds with distinct kinetics, bond energies, and responsive mechanisms. In particular, the critical question of how different types of dynamic bonds can be rationally combined within a single network to achieve properties unattainable by any single bond type has not been comprehensively reviewed. Our group has long been interested in this question. We have previously demonstrated that dynamic covalent bonds such as boronate ester and disulfide linkages can be integrated to construct multisensitive and self-healing hydrogels, and that the synergy between dynamic covalent bonds and non-covalent interactions can yield hydrogels with both robust mechanical properties and autonomous self-healing [32,33,34]. Building upon this foundation, the present review provides a systematic and unified framework that classifies multiple dynamic hydrogels by the type of non-covalent interaction synergized with dynamic covalent bonds, elucidates the underlying design principles—kinetic matching, orthogonal responsiveness, and structure–function integration—and critically evaluates the performance trade-offs across different combination strategies.

This review focuses on the combination of dynamic non-covalent interactions with dynamic covalent bonds as the central theme. It highlights multifunctional hydrogels constructed through the synergistic combination of hydrogen bonds, host–guest interactions, metal–ligand interactions, electrostatic interactions, hydrophobic interactions, and π–π stacking with dynamic covalent bonds. The network design strategies, synergistic mechanisms, and representative applications of each system are systematically analyzed, and future development trends are prospected.

## 2. Classification and Characteristics of Dynamic Bonds in Hydrogels

Understanding the chemical nature and reaction characteristics of various dynamic bonds is the foundation for the design and construction of hydrogels crosslinked by multiple dynamic bonds. This section provides a systematic overview of the types, crosslinking mechanisms, and performance characteristics of dynamic bonds commonly employed in hydrogels from the two perspectives of dynamic covalent bonds and non-covalent interactions.

### 2.1. Dynamic Non-Covalent Interactions

Dynamic non-covalent interactions are not constituted by chemical bonds involving shared electrons; rather, they rely entirely on non-covalent interactions between molecular chains to form three-dimensional networks. Like dynamic covalent bonds, these interactions are dynamic and reversible, meaning they can dissociate and reassociate under specific conditions, thereby enabling materials to exhibit “smart” characteristics such as self-healing, stimulus responsiveness, and tunable degradation [22,23]. Compared with dynamic covalent bonds, dynamic non-covalent interactions span a wider range of interaction strengths and encompass a wider variety of types, making them the core toolbox for constructing physical hydrogels.

Hydrogen bonds are among the most directional and design-flexible non-covalent interactions. They form when a hydrogen atom covalently bonded to a highly electronegative atom (e.g., oxygen or nitrogen) is electrostatically attracted to a nearby lone pair of electrons. The bond energy of a single hydrogen bond is only 5–40 kJ·mol^−1^, yet multiple hydrogen bonding arrays—such as the dimerization of ureidopyrimidinone (UPy)—can substantially enhance the overall association constant and mechanical contribution [35,36,37,38,39]. The rapid cleavage and reformation of hydrogen bonds make them ideal sacrificial bonds, capable of effectively dissipating energy under mechanical loading and immediately restoring network integrity upon unloading; their directionality and saturability also impart structural controllability to hydrogen-bonded networks, while their sensitivity to temperature, solvent environment, and pH provides a basis for designing multi-responsive hydrogels [40,41,42,43,44,45].

Host–guest interactions represent a class of highly specific molecular recognition processes in which host molecules (e.g., cyclodextrins, cucurbiturils, calixarenes) selectively encapsulate guest molecules (e.g., adamantane, azobenzene, ferrocene) through a combination of size matching, hydrophobic effects, and van der Waals forces [46,47,48]. This “lock-and-key” type of complexation endows the crosslinking network with precise stoichiometry and orthogonal responsiveness-structural changes of the guest molecule (e.g., the photoisomerization of azobenzene) or changes in its redox state (e.g., oxidation of ferrocene) can modulate the association and dissociation of the inclusion complex, thereby enabling dynamic network transitions under exogenous stimuli such as light or redox conditions [49,50,51]. During self-healing processes, host–guest recognition also plays a unique interfacial guiding role: when two fractured surfaces are brought into contact, host and guest moieties at the interface re-recognize and bind to each other, rapidly anchoring and aligning the surfaces, thereby providing ideal spatial proximity for the subsequent exchange and repair by other dynamic bonds [52,53,54].

Metal–ligand interactions (also commonly referred to as metal coordination bonds) are formed between metal ions and coordinating groups (e.g., carboxyl, catechol, histidine, phosphate). Their bond energies, which lie between those of strong covalent bonds and weak physical interactions, can be tuned over a wide range by selecting the metal ion species and oxidation state as well as by adjusting the environmental pH. Multivalent metal ions (e.g., Fe^3+^, Al^3+^) are capable of forming crosslinking junctions with a coordination number of three or even higher with multiple ligands, significantly enhancing the crosslinking density and mechanical toughness of the network while simultaneously imparting electrical conductivity, self-healing properties, and antibacterial activity to the hydrogels [55,56,57]. The reversible dissociation–re-association equilibrium of metal–ligand interactions makes them highly efficient sacrificial bonds that can absorb large amounts of energy upon loading and rapidly re-establish after unloading, thus representing an important non-covalent tool for constructing highly tough hydrogels [58,59,60,61,62,63].

Electrostatic interactions (often intuitively referred to as ionic bonds) are based on the electrostatic attraction between oppositely charged groups and are widely observed in polyelectrolyte complexes and zwitterionic polymer systems. Their strength is highly sensitive to salt concentration and pH: increasing salt concentration shields electrostatic interactions, thereby weakening ionic crosslinking, while adjusting pH alters the protonation state of ionic groups, directly affecting the number and distribution of crosslinking points. This characteristic enables rapid and reversible modulation of the mechanical properties and swelling behavior of hydrogels in response to the ionic environment. Because a large number of mobile counterions are intrinsically present within ionically crosslinked networks, such hydrogels typically possess high ionic conductivity, making them ideal candidates for flexible sensors and solid-state electrolytes [64,65,66,67,68,69,70].

Hydrophobic interactions arise from the spontaneous aggregation of non-polar groups in aqueous solution, driven primarily by the entropy gain from the release of structured water molecules. Hydrophobic moieties such as long alkyl chains and cholic acid groups form hydrophobic microdomains in water that serve as physical crosslinking points. Unlike most non-covalent interactions, hydrophobic interactions strengthen with increasing temperature, offering a unique pathway for designing thermally responsive hydrogels whose modulus does not decrease but instead increases at elevated temperatures [71,72,73,74]. Hydrophobic microdomains can also effectively encapsulate hydrophobic drug molecules, improving drug loading capacity and sustained-release performance, and enhancing adhesion in aqueous environments by excluding interfacial water molecules [75,76,77,78,79,80].

π–π stacking refers to face-to-face or edge-to-face interactions between aromatic rings, often occurring synergistically with hydrophobic effects. Pyrene, naphthalene, and phenyl groups, as well as carbon nanomaterials such as graphene and carbon nanotubes, can all form physical crosslinking points via π–π stacking. This directional interaction can drive the formation of ordered nanofibers or helical structures, imparting unique micromorphology and mechanical anisotropy to hydrogels while effectively enhancing their mechanical properties [81,82,83,84,85,86]. Carbon nanomaterials such as graphene not only provide π–π crosslinking points but also endow hydrogels with electrical conductivity and highly efficient photothermal conversion capability, enabling remote heating of the gel via near-infrared light to trigger the exchange reaction of dynamic covalent bonds or induce the reorganization of hydrophobic microdomains, thereby achieving remotely controllable self-healing and drug release [87,88,89,90,91,92,93,94].

### 2.2. Dynamic Covalent Bonds

Dynamic covalent bonds refer to covalent chemical bonds that can undergo reversible cleavage and reformation under specific conditions, combining the high bond energy characteristic of traditional covalent bonds with the reversibility of non-covalent interactions.

Schiff base bonds are formed through the condensation reaction between a primary amine (-NH_2_) and an aldehyde (-CHO) or ketone group under mild conditions without the need for an external catalyst, and the reaction can proceed under neutral or weakly alkaline conditions. Their reversibility stems from the hydrolysis of the imine bond: under acidic conditions or in the presence of excess water, the imine bond hydrolyzes, regenerating the amine and the aldehyde/ketone; upon neutralizing the pH, the condensation reaction proceeds again. This pH-dependent reversible characteristic makes Schiff base bonds an ideal choice for designing pH-responsive hydrogels [95,96,97,98]. Schiff base-crosslinked hydrogels generally exhibit favorable self-healing capability and injectability; however, owing to the relatively fast dynamic exchange rate of imine bonds in aqueous environments, the mechanical strength of a purely Schiff base-crosslinked network is often low, necessitating synergy with other crosslinking modalities to enhance overall performance [99,100,101,102].

Boronate ester bonds are formed through the reversible covalent esterification between a boronic acid group (-B(OH)_2_) and a cis-1,2- or 1,3-diol. The key feature of this reaction lies in its dual responsiveness to pH and sugar molecules: under alkaline conditions, boron exists as the tetrahedral boronate anion, which favors the formation of stable boronate ester bonds with diols; under acidic conditions, the boronate ester bond dissociates. Furthermore, free sugar molecules can compete with the diol groups for the boronic acid binding sites, thereby enabling sugar responsiveness. The exchange rate and bond strength of boronate ester bonds can be modulated by pH and molecular structure, offering considerable flexibility in the design of multifunctional hydrogels [103,104,105]. Boronate ester-crosslinked hydrogels, owing to their distinctive stimuli-responsive applications, including insulin-controlled release and glucose sensing [106,107,108,109].

Disulfide bonds (-S-S-) are covalent bonds formed between two sulfur atoms and are widely present in the maintenance of three-dimensional protein structures [110]. In hydrogel systems, disulfide bonds are typically introduced through the oxidative coupling of thiol (-SH)-containing compounds. Disulfide bonds are highly sensitive to the redox environment: under reducing conditions (e.g., in the presence of reductants such as glutathione or dithiothreitol), disulfide bonds are reduced and cleaved into thiols; under oxidizing conditions, they can be regenerated. This redox-responsive characteristic closely matches the redox potential gradient between the intracellular and extracellular environments, endowing disulfide-crosslinked hydrogels with distinct advantages in drug delivery and the controlled release of bioactive molecules [111,112,113,114,115]. The bond energy of disulfide bonds is relatively high, and purely disulfide-crosslinked networks exhibit comparatively superior mechanical properties; however, their self-healing typically requires chemical stimuli or thermal assistance.

Acylhydrazone bonds are generated through the condensation of a hydrazide group (-CONHNH_2_) with an aldehyde or ketone group. They are structurally analogous to Schiff base bonds but possess higher hydrolytic stability owing to the conjugation effect of the adjacent carbonyl group. The exchange rate exhibits moderate dynamic behavior under neutral and weakly acidic conditions. Hydrogels based on acylhydrazone bonds often exhibit injectability, and, because the reaction conditions are mild, are particularly suitable for use in combination with bioactive molecules [116,117,118,119].

Diels–Alder adducts are obtained through the Diels–Alder reaction, a [4 + 2] cycloaddition between a diene and a dienophile. They exhibit temperature-dependent reversible behavior: at lower temperatures, the forward reaction forms the adduct; at higher temperatures, the retro-Diels–Alder reaction occurs, leading to dissociation. This thermal reversibility makes them a distinctive tool for designing temperature-responsive hydrogels [120,121,122,123].

### 2.3. Design Principles for the Synergistic Integration of Multiple Dynamic Bonds

The integration of two or more types of dynamic bonds into a single network enables them to assume different yet complementary functional roles during mechanical loading and repair processes. These bonds possess distinct bond energies, exchange kinetics, and stimuli-responsive characteristics. The first is the principle of kinetic matching: the cleavage-reformation rates of the various dynamic bonds within the system should form an ordered gradient distribution. The bonds with the fastest exchange rates—typically hydrogen bonds or electrostatic crosslinks among non-covalent interactions—are the first to rupture under stress. These bonds act as sacrificial units that dissipate energy and instantly reform upon unloading. Bonds with intermediate exchange rates (such as metal coordination or hydrophobic associations) undertake secondary energy dissipation and medium-term structural maintenance. The bonds with the slowest exchange rates (dynamic covalent bonds) serve as the permanent or semi-permanent skeleton of the network, preserving the overall shape and elastic recovery. This fast–intermediate–slow kinetic cascade ensures that the hydrogel possesses corresponding dissipation and repair mechanisms across different timescales of mechanical loading, circumventing the problems of insufficient dissipation or excessive creep encountered by single-bond systems within a given frequency range. From a quantitative perspective, the exchange kinetics of different dynamic bonds span approximately six orders of magnitude in relaxation time. Hydrogen bonds and electrostatic interactions typically exhibit relaxation times on the order of milliseconds to seconds (τ ≈ 10^−3^–10^0^ s), enabling instantaneous energy dissipation and rapid interfacial re-establishment [22,23]. Metal–ligand coordination bonds display tunable kinetics depending on the metal ion species: Cu^2+^-based coordination exchanges on the timescale of ~0.1 s, Zn^2+^ on ~10 s, and Ni^2+^ on >100 s, offering a precisely controllable kinetic window from fast to slow [124]. Host–guest inclusion complexes exhibit binding constants ranging from 10^3^ to 10^13^ M^−1^, with dissociation half-lives varying from seconds (e.g., α-CD/Azo under UV irradiation) to hours (e.g., β-CD/Ada under physiological conditions) [125,126,127]. Dynamic covalent bonds, in contrast, generally exhibit much slower exchange kinetics, with characteristic relaxation times ranging from minutes to hours depending on the specific chemistry and environmental conditions: Schiff base bonds typically exchange within minutes to hours under acidic catalysis [95,96,97,98], boronate ester bonds within tens of minutes to hours with pH dependence [103,104,105], and disulfide bonds within hours under redox stimuli [110,111,112,113,114,115]. This wide kinetic window—from milliseconds for the fastest non-covalent interactions to hours for the slowest dynamic covalent bonds—provides the fundamental timescale separation necessary for hierarchical energy dissipation and cascade self-healing. The second is the principle of stimuli-responsive orthogonality: different types of dynamic bonds should respond to different environmental stimuli without mutual interference. Orthogonal responsiveness enables the hydrogel to execute differentiated mechanical changes in complex environments. One stimulus modulates the physical crosslinking density of the network. At the same time, another alters the chemical crosslinking state—the two operate independently yet can synergistically superimpose, thereby achieving stepwise mechanical regulation unattainable by a single-responsive system. The third is the principle of structure–function integration: the Introduction of non-covalent interactions not only provides mechanical contributions but also enables the full exploitation of the additional functionalities they inherently carry. Integrating these functions with the self-healing ability and chemical responsiveness of dynamic covalent bonds enables a leap from purely mechanical reinforcement to multifunctional integration.

These three principles are not independent of one another but are interrelated. Kinetic matching determines the temporal coordination of the various bond types during mechanical loading and self-healing processes, stimuli-responsive orthogonality dictates how the material senses and responds to multidimensional environmental signals, and structure–function integration unifies mechanical properties and additional functionalities within the same network. The organic combination of the three is key to transforming the design of multiple dynamic bond synergy from empirical attempts toward rational construction. The following sections will, based on the above design principles, sequentially elaborate on the specific implementation strategies, synergistic mechanisms, and application characteristics of the synergistic integration of various types of dynamic non-covalent interactions with dynamic covalent bonds.

## 3. Multiple Dynamic Bond-Crosslinked Hydrogels

Crosslinking strategies for hydrogels have shifted from single dynamic bonds to the synergistic integration of multiple dynamic bonds. Single-dynamic crosslinked hydrogels can impart reversible, self-healing properties to the network. This applies whether they rely on one type of dynamic covalent bond (e.g., Schiff base bonds, boronate ester bonds, or disulfide bonds) or one type of non-covalent interaction (e.g., hydrogen bonds or electrostatic interactions). However, the inherent kinetic and energetic constraints of a single bonding mode make it difficult to simultaneously optimize multiple performance metrics, including mechanical strength, self-healing rate, stimuli-responsiveness, and processability. Purely dynamic covalent networks provide robust structural integrity, but their slow bond-exchange kinetics often require external stimuli to trigger healing, hindering spontaneous repair under physiological conditions. Conversely, purely non-covalent networks exhibit rapid self-healing but suffer from insufficient mechanical strength, making them susceptible to creep and irreversible deformation under sustained loading.

Multiple dynamic bond-crosslinked hydrogels overcome the performance ceiling of single dynamic bond systems by integrating two or more types of dynamic bonds with distinct bond energies, exchange kinetics, and stimuli-responsive characteristics into a single network. These bonds are assigned complementary functional roles—structural framework maintenance, energy dissipation, rapid self-healing, and multi-stimuli responsiveness. The advantages of this strategy are threefold. First, at the mechanical level, weak bonds with fast exchange kinetics (e.g., hydrogen bonds, electrostatic crosslinks) act as sacrificial bonds that preferentially rupture upon loading to dissipate energy, while strong bonds with slow exchange kinetics (dynamic covalent bonds) maintain the elastic framework. This hierarchical energy dissipation mechanism imparts a combination of high strength, high toughness, and fatigue resistance. Second, at the self-healing level, weak bonds rapidly re-form upon damage, bringing fractured surfaces into immediate contact, followed by permanent covalent repair mediated by strong bonds. This cascade of fast and slow bond dynamics reconciles the often-conflicting demands of healing rate and healing efficiency. Third, at the functional integration level, distinct dynamic bonds respond to different environmental signals in an orthogonal and non-interfering manner. Non-covalent interactions are sensitive to temperature, ionic strength, or light, whereas dynamic covalent bonds respond to pH or redox conditions, thereby endowing the hydrogel with multi-modal stimuli-responsiveness and programmable deformation. Furthermore, the intrinsic functionalities of non-covalent interactions are seamlessly integrated with the self-healing and chemical responsiveness of dynamic covalent bonds. These functionalities include tissue adhesiveness from hydrogen bonds, ionic conductivity from electrostatic interactions, hydrophobic drug loading from hydrophobic domains, photothermal conversion from π–π stacking, and antibacterial or catalytic activity from metal coordination, enabling a leap from purely mechanical reinforcement to multifunctional integration.

The core of multiple dynamic bond-crosslinked hydrogel design lies in the synergistic combination of non-covalent interactions with dynamic covalent bonds. Based on the type of non-covalent interaction employed, the systems reported to date can be classified into six principal strategies: (i) hydrogen bonds + dynamic covalent bonds, (ii) host–guest recognition + dynamic covalent bonds, (iii) metal–ligand interactions + dynamic covalent bonds, (iv) electrostatic interactions + dynamic covalent bonds, (v) hydrophobic interactions + dynamic covalent bonds, and (vi) π–π stacking + dynamic covalent bonds. Additionally, emerging systems that hierarchically integrate multiple non-covalent interactions with dynamic covalent bonds, or that incorporate nanomaterials and biological macromolecules, continue to expand the scope of this field. The following sections sequentially elaborate on the construction methodologies, synergistic mechanisms, and functional characteristics of each strategy.

To provide readers with a clear roadmap, Table 1 summarizes the key challenges identified in the Introduction and maps them to the corresponding strategies elaborated in Section 3.1, Section 3.2, Section 3.3, Section 3.4, Section 3.5 and Section 3.6. Each strategy is designed to address one or more of these challenges, and the subsequent subsections will demonstrate how integrating different dynamic bonds progressively overcomes the limitations of single-bond systems.

### 3.1. Synergistic Integration of DCBs and Hydrogen Bonds

Hydrogen bonds, owing to their directionality, reversibility, and nearly ubiquitous design basis, represent the most versatile non-covalent component in multiple dynamic bond-crosslinked hydrogels. The synergistic combination of hydrogen bonds with dynamic covalent bonds constitutes the most extensively investigated and widely applied category of such systems, and the design principles and optimization strategies developed for these systems have laid the foundation for subsequent studies on other combination types. The core synergistic logic of integrating hydrogen bond physical crosslinking with dynamic covalent bonds lies in the following: hydrogen bonds serve as the “fast bonds” responsible for rapid energy dissipation and immediate self-healing. In contrast, dynamic covalent bonds act as the “slow bonds” that maintain the elastic framework and long-term structural stability of the network. The cascade coordination of these two types of bonds in terms of kinetics is key to achieving high toughness, rapid self-healing, and fatigue resistance. In gel fabrication, the synergy between hydrogen bonds and dynamic covalent bonds is primarily realized through three strategies: double-network interpenetration, single-network multiple crosslinking, and polyurethane hard-segment hydrogen bonding, which are respectively suited for the construction of polysaccharide-based highly extensible hydrogels, synthetic polymer-based homogeneous networks, and polyurethane-based high-strength hydrogels.

The double-network interpenetration strategy constructs two relatively independent yet mutually interpenetrating crosslinked networks—a dynamic covalent network that serves as a structural framework and for elastic recovery, and a hydrogen-bonded physical network responsible for energy dissipation and rapid self-healing. The two networks are spatially interpenetrated but kinetically distinct: under external force, the hydrogen-bonded network is the first to rupture and dissipate energy, while the covalent framework maintains structural integrity; upon unloading, the hydrogen bonds rapidly reassociate to restore the network, while the covalent bonds repair residual structural damage through slow exchange. This “dual-network division of labor” synergistic mode endows the hydrogel with both high extensibility and rapid self-healing capability.

**Figure 1:**
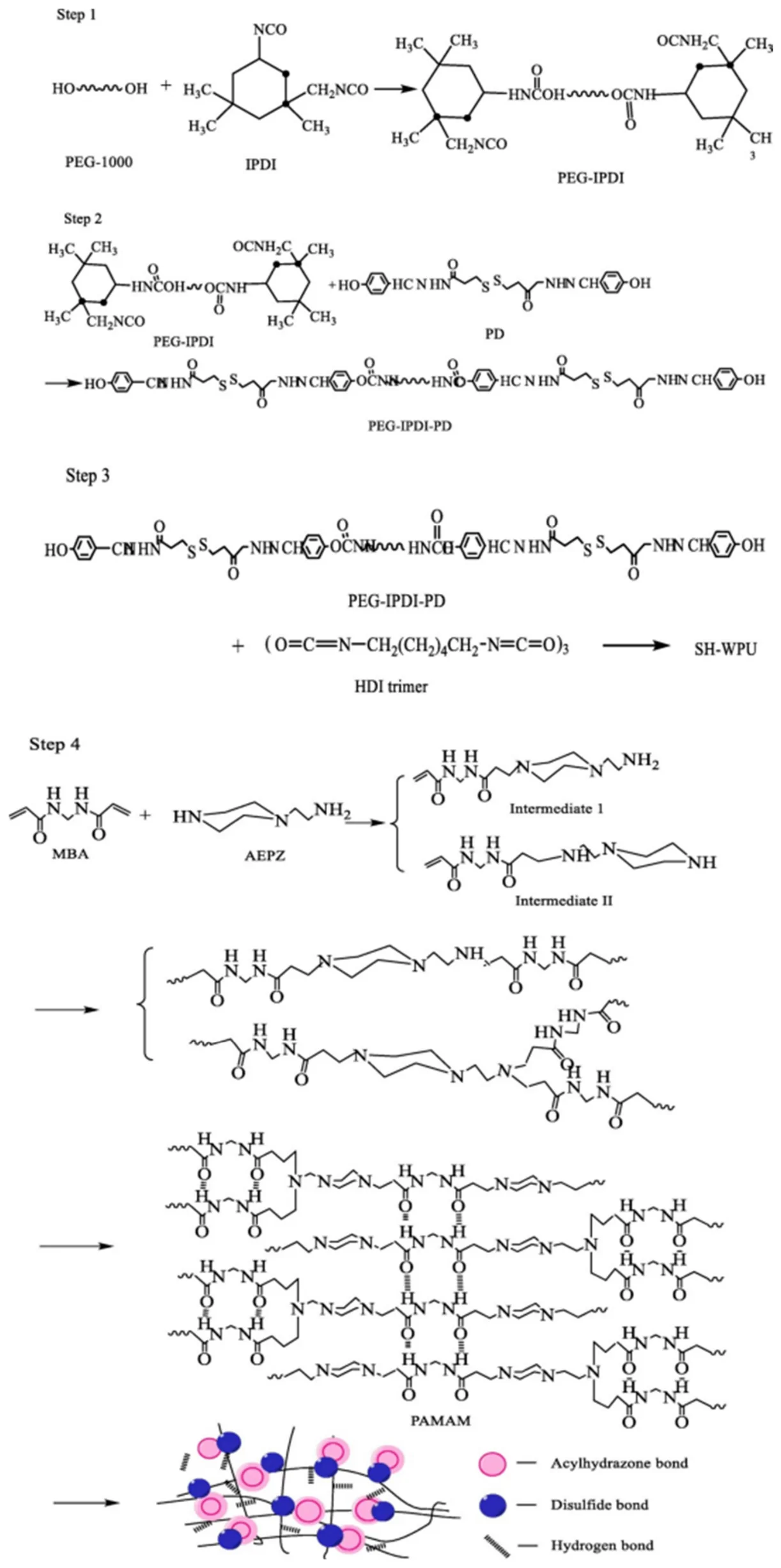
Synthesis route of the SH-WPU/PAMAM full-IPN hydrogel and visual correspondence to the dual-network kinetic cascade mechanism. PEG-1000 reacts with IPDI to form isocyanate-terminated PEG-IPDI (Intermediate I). PD (dihydric alcohol containing acylhydrazone and disulfide bonds) reacts with PEG-IPDI to yield PEG-IPDI-PD (Intermediate II). HDI trimer crosslinks PEG-IPDI-PD chains to form the SH-WPU network. The SH-WPU network is interpenetrated by crosslinked PAMAM to form the full-IPN structure, with hydrogen bonds densely distributed between PAMAM chain segments. Acylhydrazone and disulfide bonds in the SH-WPU backbone constitute the slow-exchanging covalent scaffold (structural framework and elastic recovery). Hydrogen bonds within PAMAM constitute the fast-reassociating physical network (energy dissipation and rapid interfacial re-contact). These two networks are spatially interpenetrated but kinetically distinct—fast H-bond rupture/recovery vs. slow covalent exchange—forming the mechanistic basis for their synergistic “division-of-labor” self-healing mode. Reprinted with permission from Ref. [128].

For instance, Huang et al. [128] developed a self-healing interpenetrating network (IPN) hydrogel that exploits a dual dynamic synergistic mechanism—reversible covalent bonds (disulfide and acylhydrazone bonds) and reversible non-covalent hydrogen bonds—forming a full-IPN architecture. In this polyurethane/polyamide amine (SH-WPU/PAMAM) DN hydrogel, dynamic covalent bonds (disulfide and acylhydrazone) serve as reversible chemical crosslinks within the first network, providing the structural framework and enabling self-healing under acidic, alkaline, or light conditions, while hydrogen bonds within the second PAMAM network act as physical crosslinks that are responsible for rapid energy dissipation and fast initial recovery. This synergy achieves a healing efficiency of 87.35% after 48 h at room temperature under light irradiation, with significant recovery even within 1 h (31.58%), while maintaining good mechanical strength (tensile strength of 0.77 MPa) and high swelling capacity (equilibrium swelling ratio of 354%), demonstrating excellent multi-environment adaptability and long-term self-healing performance (Figure 1). These features render this IPN hydrogel particularly suitable for applications requiring long-term durability in chemically or photochemically aggressive environments, such as protective coatings and biomedical sealants, where the combination of covalent framework stability and hydrogen-bond-mediated energy dissipation is essential.

Wang et al. [129] developed a self-healing ultra-stretchable hydrogel that exploits a dual dynamic synergistic mechanism—boronate ester bonds and hydrogen bonds forming an interpenetrating double network. In this PVA/Alg-PBA/Gel composite hydrogel, boronate ester dynamic covalent bonds serve as reversible chemical crosslinks, imparting rapid room-temperature self-healing ability (initiating within 30 s), while hydrogen bonds act as physical crosslinks that enhance network extensibility and structural stability, achieving ultra-stretchability of up to 1200%. Such ultra-stretchability, combined with rapid room-temperature healing, makes this hydrogel promising for postharvest logistics as a protective coating for fresh produce, where resistance to mechanical damage and instant repair upon puncturing are critical.

Jia et al. [130] developed a multifunctional bio-based wearable ionogel that exploits a hierarchical, dynamic covalent, crosslinked double-network synergistic mechanism—dynamic disulfide bonds, hydrogen bonds, and Bmim^+^-O coordination bonds—forming an interpenetrating double network. In this LA-XEA ionogel based on lipoic acid and alkoxyl xylan, dynamic disulfide covalent bonds serve as reversible chemical crosslinks, imparting rapid room-temperature self-healing capability (98.92% healing efficiency within 12 h), while hydrogen bonds and Bmim^+^-O coordination bonds act as physical crosslinks that enhance network extensibility and structural stability, achieving ultra-stretchability of up to 1500%. The bio-based nature and high stretchability of this ionogel position it as a candidate for wearable flexible electronics and motion sensors, though its long-term stability under sweat exposure and repeated mechanical cycling warrants further investigation.

Ren et al. [131] developed a ROS-responsive bioactive double-network hydrogel that exploits a triple dynamic synergistic mechanism—hydrogen bonds, phenylboronate ester bonds, and acylhydrazone bonds forming an interpenetrating double network. In this PD-OHN hydrogel based on PVA, DPBA, OHA, and HA-ADH, phenylboronate ester dynamic covalent bonds serve as reversible chemical crosslinks, imparting rapid self-healing capability, while hydrogen bonds and acylhydrazone bonds act as physical crosslinks that enhance network stability and tissue adhesion, achieving good stretchability and suitable in vivo retention.

The double-network interpenetration strategy, while successfully achieving the synergy of hydrogen bonds and dynamic covalent bonds, spatially separates the two types of bonds into distinct networks. This spatial segregation limits the efficiency of their molecular-level synergistic interaction, as energy dissipation and framework stabilization occur in separate domains rather than being intimately coupled. To overcome this limitation, the single-network hydrogen-bond-forming polar groups and dynamic covalent-reactive functional groups are simultaneously modified onto the same polymer chain. During gelation, two types of dynamic bonds form in situ at different sites along the same chain, creating a structurally more homogeneous dual dynamic network. In this configuration, the energy released from hydrogen bond rupture upon loading is directly transferred to adjacent dynamic covalent bonds, enabling nearly synchronous energy dissipation and framework protection. During self-healing, the rapid anchoring by hydrogen bonds and the exchange-based repair by dynamic covalent bonds are seamlessly integrated at the nanoscale. This molecular-level intimacy significantly enhances the synergistic efficiency compared to the spatially separated double-network architecture.

The single-network, multiple crosslinking strategy simultaneously modifies the same polymer chain with hydrogen-bond-forming polar groups and functional groups capable of participating in dynamic covalent reactions. During the gelation process, two types of dynamic bonds form in situ at different sites along the same chain, resulting in a structurally more homogeneous dual dynamic network. Compared with the double-network strategy, its synergy with dynamic covalent bonds is more direct and efficient—hydrogen bonds and dynamic covalent bonds are in close proximity at the molecular scale. Under external force, the energy released from hydrogen bond rupture is directly transferred to adjacent dynamic covalent bonds, enabling nearly synchronous energy dissipation and framework protection. During self-healing, the rapid anchoring by hydrogen bonds and the exchange-based repair by dynamic covalent bonds are seamlessly integrated at the nanoscale. The synergistic logic of this strategy has been validated across a variety of dynamic covalent bond systems.

**Figure 2:**
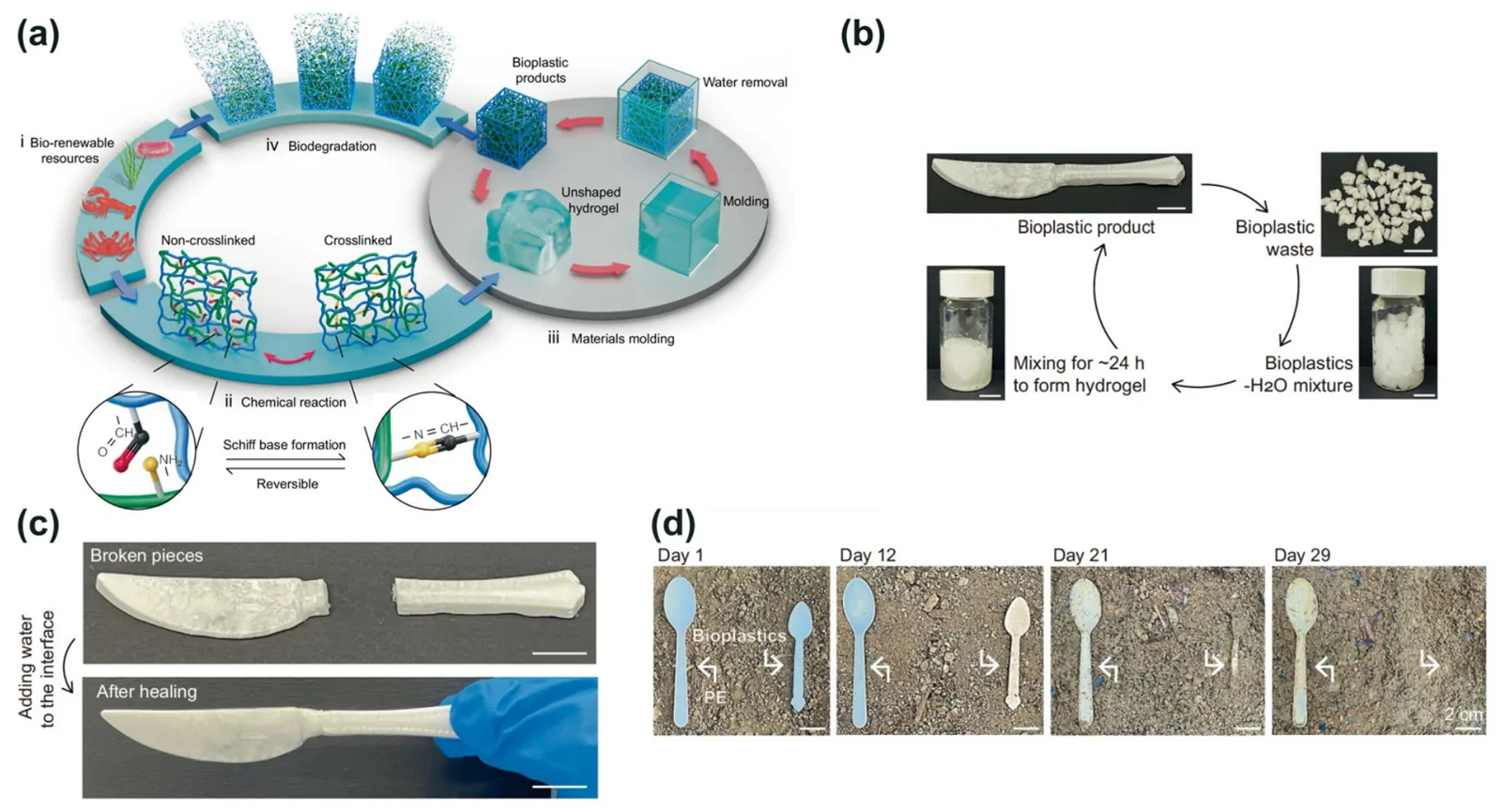
Sustainable DNA-polysaccharide hydrogels exemplifying the single-network multiple crosslinking synergistic mechanism. (**a**) Illustration of the multiple closed-loop cycles of the DNA-polysaccharide-based bioplastics: Cycle I from bio-renewable resources (**i**) through chemical reaction (**ii**) and materials molding (**iii**) to biodegradation (**iv**); Cycle II enabled by reversible imine bond chemistry (**ii**); and Cycle III realized through water-recyclable molding (**iii**). The reversible imine dynamic covalent bonds serve as the chemical foundation for both closed-loop recyclability and reprocessability. (**b**) Schematic of a typical water-processable recycling process, where the hydrogel precursors are re-dissolved and re-gelled without loss of network integrity. (**c**) Photographs of the aqua-healing process: a knife-shaped hydrogel, upon fracture, reconnects within seconds upon water application at the interface, and the healed product sustains stretching without failure. This rapid self-healing arises from the synergistic interplay of fast hydrogen bond reformation (instantaneous interfacial anchoring) and slow imine bond exchange (permanent covalent restoration), achieving seamless integration of rapid recovery and long-term stability. (**d**) Photographs of the biodegradation process of the bioplastics compared with a PE spoon under real-world soil conditions over 29 days. The complete degradation of the DNA-polysaccharide hydrogel reflects the dynamic and reversible nature of both the covalent (imine) and non-covalent (hydrogen bond) crosslinks, which enable network disintegration under environmental stimuli. Adapted with permission from Ref. [132].

For example, Ke et al. [132] developed a sustainable DNA-polysaccharide hydrogel that exploits a single-network multiple crosslinking synergistic mechanism—Schiff base imine bonds and hydrogen bonds forming a homogeneous dual dynamic network on the same polymer chains. In this Dex-DNA hydrogel derived from oxidized dextran and biomass DNA, imine dynamic covalent bonds serve as reversible chemical crosslinks, imparting aqueous healing capability (no significant change in Young’s modulus after healing), while hydrogen bonds act as physical crosslinks that enhance network cohesion and structural stability. Importantly, the bioplastic exhibits multi-closed-loop recyclability through water-based processing (maintaining mechanical properties after 10 recycling cycles) and complete biodegradability in soil within 29 days or enzymatic degradation within 120 min using DNase I, offering a sustainable alternative to conventional petroleum-based plastics (Figure 2). The injectability and rapid gelation of this system make it particularly suitable for minimally invasive drug delivery and cell encapsulation, where the ability to flow through a syringe and rapidly solidify at the target site is paramount.

Huang et al. [133] developed a glucose-responsive, self-healing wet-adhesive hydrogel that exploits a single-network, multiple crosslinking synergistic mechanism—boronate ester dynamic covalent bonds and hydrogen bonds—forming a homogeneous dual-dynamic network. In this PAE hydrogel based on poly(acrylic acid-co-acrylamide) and an E-F crosslinker (EGCG–AFPBA), boronate ester dynamic covalent bonds serve as reversible chemical crosslinks, imparting rapid room-temperature self-healing ability (interfacial healing within 3 min and full recovery over repeated cutting–healing cycles, as confirmed by step-strain rheological tests), while hydrogen bonds act as physical crosslinks that enhance network cohesion and structural stability. Importantly, the PAE hydrogel exhibited a distinct glucose-responsive drug release behavior, with the cumulative release of EGCG significantly accelerated in a high-glucose solution (25 mM) compared to PBS, enabling sustained, on-demand therapeutic delivery tailored to the diabetic wound microenvironment.

Chen et al. [134] developed a lignin-based supramolecular hydrogel that exploits a single-network multiple crosslinking synergistic mechanism—disulfide dynamic covalent bonds and hydrogen bonds—forming a homogeneous dual-dynamic network on the same polymer chains. In this GpLSH hydrogel derived from chemically modified lignin (L-cystine graft esterification polymerization of glyoxal-polymerized alkali lignin), disulfide dynamic covalent bonds serve as reversible energy-dissipating crosslinks, imparting low energy dissipation (minimum 5.58 kJ/m^3^) and excellent creep resistance (93% recovery within 3 min after unloading), while hydrogen bonds, ionic bonds and hydrophobic interactions act as physical crosslinks that enhance network cohesion and structural stability, achieving outstanding flexibility with an elongation at break of up to 1250% and a fracture stress of 0.95 MPa.

Guo et al. [135] developed a tough and biocompatible hydrogel that exploits a dual dynamic synergistic mechanism—acylhydrazone dynamic covalent bonds and hydrogen bonds forming an interpenetrating double network. In this P(AAm-co-DAAm)/PVP hydrogel, acylhydrazone dynamic covalent bonds serve as reversible chemical crosslinks, imparting rapid room-temperature self-healing ability (up to 87.5% healing efficiency within 10 h) and pH-switchable injectability, while hydrogen bonds act as physical crosslinks that enhance network cohesion and structural stability, achieving fast self-recovery (ca. 91% toughness recovery within 1.0 min) and excellent fatigue resistance (maintaining mechanical performance over 10 successive loading–unloading cycles).

González et al. [136] developed a starch-based hydrogel that exploits a dual-dynamic synergistic mechanism—Diels–Alder (DA) adduct dynamic covalent bonds and hydrogen bonds, forming a single-network dual-crosslinking architecture. In this furan-modified starch/bismaleimide (S-FI/BMI) hydrogel, DA adduct bonds serve as reversible covalent crosslinks, imparting effective network formation and structural stability (storage modulus increased from 1600 Pa to 3750 Pa with increasing crosslinker content), while hydrogen bonds between starch chains act as physical crosslinks that enhance network cohesion and water retention, achieving tunable swelling behavior (equilibrium swelling ratio up to ~800%) and improved resistance to hydrolytic degradation (weight loss reduced from 79% to ~52% with higher crosslinking density).

Despite the enhanced synergy achieved by the single-network dual-crosslinking strategy, the resulting hydrogels still possess only a single chemically responsive dimension, typically responding to either pH or redox conditions but not both simultaneously. To address this limitation, researchers have further introduced two different types of dynamic covalent bonds together with hydrogen bonds into the same single-network system, constructing triple composite dynamic crosslinking networks. In this design, the two types of dynamic covalent bonds respond to distinct chemical signals—for instance, one to pH and the other to redox conditions—in an orthogonal, non-interfering manner, while hydrogen bonds continue to serve as rapidly reversible sacrificial bonds, providing energy dissipation and interfacial anchoring functions. This “one non-covalent + two covalent” design enables the gel to retain rapid self-healing while acquiring multidimensional chemically orthogonal responsiveness and viscoelastic tunability over a wider timescale, representing the frontier direction of the single-network multiple crosslinking strategy as it extends from dual crosslinking to multi-covalent crosslinking.

**Figure 3:**
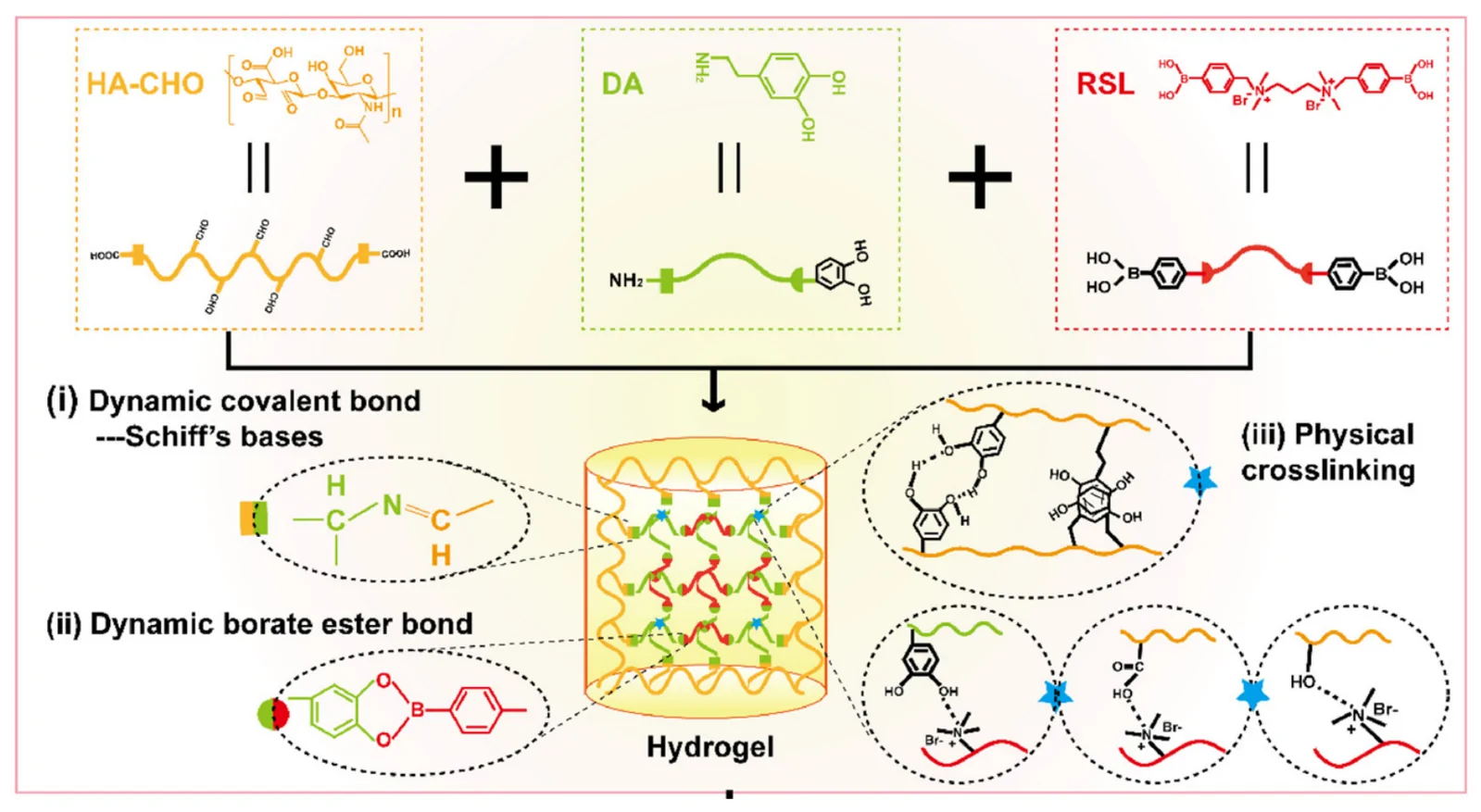
Schematic diagram of the formation of the Lipo/CXB@Hydrogel, where three reversible crosslinking interactions are marked: pH-responsive Schiff base dynamic covalent bonds, ROS-sensitive boronate ester bonds, and hydrogen bonds alongside π–π stacking physical crosslinks coexist on identical polymer backbones. The two distinct dynamic covalent moieties visualized deliver orthogonal stimulus responses, while hydrogen bond aggregates act as fast sacrificial supramolecular units, visually demonstrating the kinetically cascaded, triple-composite crosslinking synergistic system. Adapted with permission from Ref. [137].

For example, Zhao et al. [137] developed a multifunctional hydrogel that exploits a synergistic triple-composite dynamic crosslinking mechanism—Schiff base bonds, boronate ester bonds, and hydrogen bonds—forming a single-network triple-crosslinking architecture. In this dopamine-crosslinked oxidized hyaluronic acid (HA-CHO)/reactive oxygen species-sensitive linker (RSL) hydrogel, Schiff base and boronate ester dynamic covalent bonds serve as reversible crosslinks responsive to pH and ROS respectively, imparting rapid self-healing capability (recovering within 2 min and maintaining stability over repeated step-strain cycles), while hydrogen bonds and π–π stacking act as physical crosslinks that enhance network cohesion, achieving strong tissue adhesion (adhesive strength of 39.5 kPa, 2.6 times higher than commercial fibrin glue), stable mechanical properties (G′ consistently exceeding G″ across the frequency sweep range), and tunable ROS-responsive degradation (complete degradation within 2 weeks in H_2_O_2_, with sustained drug release of 89.3% for CXB and 83.3% for Lipo under oxidative conditions) (Figure 3). This triple-component network is specifically designed for postoperative wound management and obesity-associated tumor immunotherapy, where the combination of strong tissue adhesion, ROS-responsive degradation, and sustained drug release addresses the critical need for localized therapy in the inflammatory tumor microenvironment.

Xie et al. [138] developed a double dynamic covalent cellulose-based hydrogel that exploits a triple composite dynamic crosslinking synergistic mechanism—Schiff base bonds, disulfide bonds, and hydrogen bonds—forming a single-network triple-crosslinking architecture. In this UV-cured cellulose nanofiber/GelMA/F127DA hydrogel, Schiff base dynamic covalent bonds serve as reversible interfacial crosslinks between the hydrogel and tissue surfaces (achieving a strong adhesion strength of 215.7 kPa), while disulfide bonds act as reversible crosslinks within the hydrogel skeleton, imparting triggerable detachment capability upon treatment with GSH/acetic acid detachment solution (enabling benign separation without secondary tissue damage, as confirmed by H&E staining), and hydrogen bonds between cellulose chains act as physical crosslinks, enhancing network cohesion and mechanical stability (fracture strength of ~26.9 kPa).

Zhang et al. [139] developed an injectable self-healing hydrogel that exploits a synergistic dynamic crosslinking mechanism—imine bonds, acylhydrazone bonds, disulfide bonds, and hydrogen bonds—forming a single-network multi-crosslinking architecture. In this quaternized carboxymethyl chitosan/oxidized hyaluronic acid (QCMCS/OHA) hydrogel with DTP crosslinker, imine and acylhydrazone dynamic covalent bonds serve as reversible pH-responsive crosslinks, while disulfide bonds act as redox-responsive crosslinks, collectively imparting rapid self-healing capability (96% healing efficiency within 30 min), and hydrogen bonds act as physical crosslinks that enhance network cohesion and structural stability, resulting in good mechanical properties (compressive stress of 423 kPa), excellent pH responsiveness, and injectability (sol–gel transition within 32 s, enabling minimally invasive delivery).

The aforementioned strategies—whether double-network interpenetration, single-network dual-crosslinking, or triple composite crosslinking—all rely on the homogeneous distribution of hydrogen bonds and dynamic covalent bonds throughout the network. However, this homogeneous distribution inherently couples the dynamics of the two bond types: the rupture of hydrogen bonds and the exchange of covalent bonds occur within the same spatial domains, potentially leading to mutual interference. To achieve true spatial decoupling of mechanical reinforcement and dynamic reversibility, the polyurethane hard-segment strategy offers a fundamentally different approach. By exploiting the microphase-separated structure inherent to polyurethane systems, this strategy concentrates high-density hydrogen bond arrays within hard-segment microdomains, which act as physical crosslinking regions analogous to nanofillers, providing high-strength sacrificial bonds and energy dissipation. Meanwhile, dynamic covalent crosslinks are introduced into the soft-segment domains, providing elastic recovery and redox responsiveness. The spatial separation of the hydrogen bond domains and covalent crosslinking domains ensures that their dynamic behaviors do not interfere with each other: hydrogen bond rupture does not compromise the integrity of the covalent framework, and covalent exchange does not disrupt the structure of the hydrogen bond domains. This spatial decoupling strategy not only achieves superior mechanical reinforcement and self-healing efficiency but also represents a distinct design philosophy that complements the homogeneous approaches discussed above.

The polyurethane hard-segment strategy exploits the ability of urethane groups in the polyurethane backbone to form high-density multiple hydrogen bond arrays within hard-segment microdomains, while simultaneously introducing dynamic covalent crosslinking points through chain extenders or post-crosslinking reactions. The synergy with dynamic covalent bonds relies on the microphase-separated structure—the densely packed hydrogen bonds in the hard-segment domains form physical crosslinking regions analogous to nanofillers, providing high-strength sacrificial bonds and energy dissipation, while the dynamic covalent crosslinks in the soft-segment domains provide elastic recovery and redox responsiveness. The spatial separation of the hydrogen bond domains and covalent crosslinking domains ensures that their dynamic behaviors do not interfere with each other: hydrogen bond rupture does not compromise the integrity of the covalent framework, and covalent exchange does not disrupt the structure of the hydrogen bond domain, thereby achieving spatial decoupling of mechanical reinforcement and dynamic reversibility [140].

**Figure 4:**
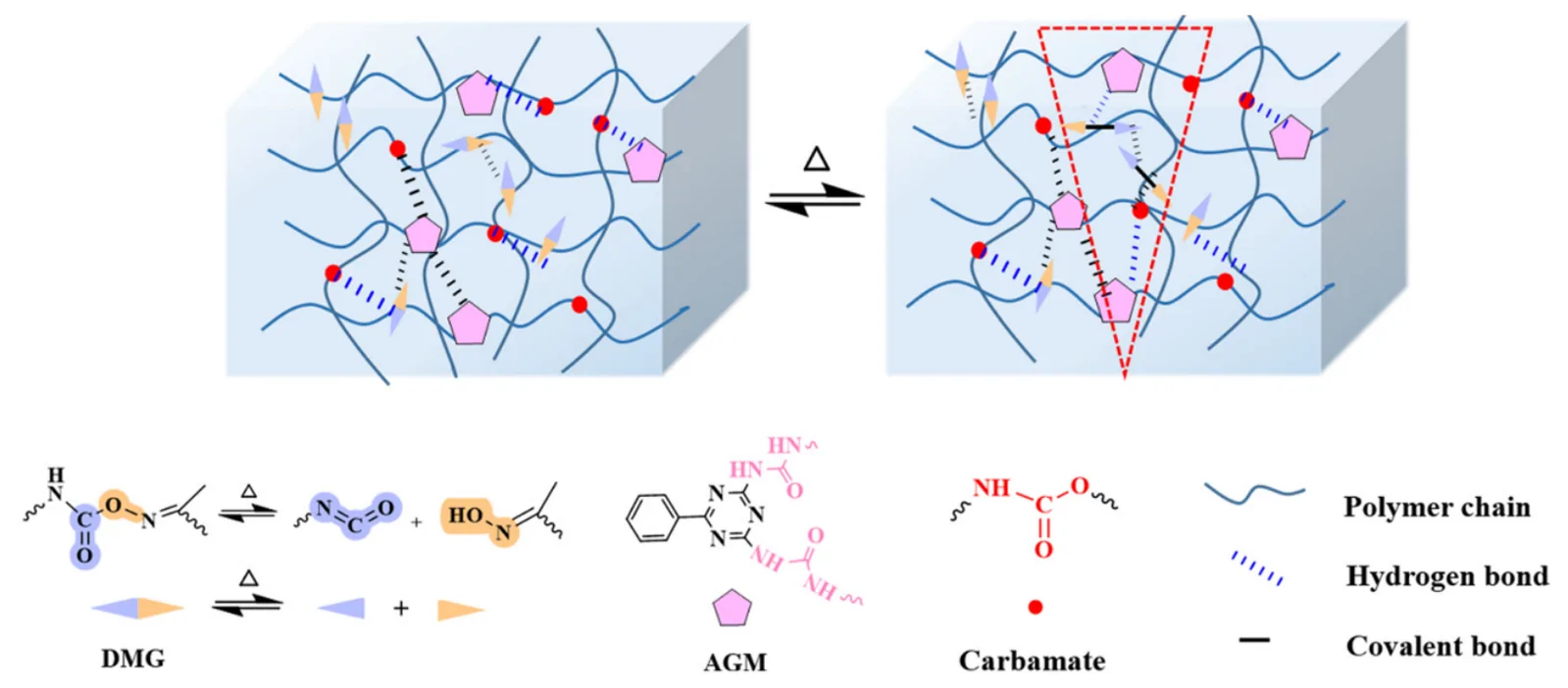
Schematic diagram of the self-healing mechanism of D_x_A_y_WPU films. The region highlighted by the red dotted triangle magnifies the orthogonal multi-dynamic synergistic interactions. Visible graphic elements are matched to orthogonal, spatially decoupled multi-dynamic synergistic behavior: purple triazine moieties alongside urea-urethane linkages construct compact hydrogen-bond hard microdomains that function as fast sacrificial physical crosslinks for energy dissipation under tension; thermo-responsive oxime–carbamate dynamic covalent bonds spread over soft polymer chains act as recoverable elastic skeletons. The spatially isolated two dynamic domains exhibit non-interfering kinetic cascades: hydrogen bonds break preferentially to absorb strain energy without destroying the covalent network, while thermal stimulation drives reversible cleavage and reformation of oxime–carbamate bonds to reconstruct fractured polymer frameworks, directly visualizing the hard-segment design strategy that spatially separates mechanical reinforcement and dynamic reversibility. Adapted with permission from Ref. [141].

For example, Zhang et al. [141] developed a tough and self-healing waterborne polyurethane elastomer (D_x_A_y_WPU) that exploits a multi-dynamic synergistic mechanism—oxime-carbamate dynamic covalent bonds and high-density multiple hydrogen bonds (from urea-urethane linkages and triazine rings)—forming a microphase-separated soft–hard segment architecture. In this PU elastomer based on isophorone diisocyanate (IPDI), dimethylglyoxime (DMG), 6-methyl-1,3,5-triazine-2,4-diamine (AGM), and polytetramethylene ether glycol (PTMEG), oxime-carbamate dynamic covalent bonds serve as reversible chemical crosslinks that enable thermally driven self-healing, while the densely packed hydrogen bond arrays in the hard-segment microdomains (facilitated by AGM and urethane groups) act as physical crosslinks that enhance network cohesion and energy dissipation. This synergy achieves outstanding mechanical performance—tensile strength of 33.04 MPa, elongation at break of 954.79%, toughness of 90.66 MJ/m^3^—and excellent self-healing efficiency of 90.1% after 24 h at 80 °C, with the elastomer also demonstrating stable electrical response and repeatable sensing capability when integrated with carbon nanotubes (CNTs) for wearable flexible strain sensors (Figure 4). The combination of high tensile strength (33.04 MPa) and healing efficiency (90.1%) positions this elastomer for durable wearable strain sensors that can withstand repeated mechanical loading. However, the thermal requirement for healing (80 °C) may limit its application in temperature-sensitive settings such as on-skin electronics.

Fang et al. [142] developed a self-healable and recyclable polyurethane-polyaniline hydrogel that exploits a dual dynamic synergistic mechanism—hydrogen bonds, ionic interactions, and Diels–Alder dynamic covalent bonds—that form an interpenetrating, synergistic double network. In this PU-DA-1/1-PANI hydrogel based on furan-functionalized polyurethane and bismaleimide crosslinker with in-situ polymerized polyaniline, DA covalent bonds serve as reversible chemical crosslinks, imparting thermal-triggered self-healing capability (77.6% tensile strength and 89.6% elongation recovery upon thermal healing at 125 °C, and 49.4% strength and 65.4% elongation recovery at room temperature within 2 h), while hydrogen bonds within polyurethane hard-segment microdomains and ionic interactions between PANI and phytic acid act as physical crosslinks that enhance network cohesion and energy dissipation, achieving excellent stretchability (elongation at break of 500%, tensile strength of 1.1 MPa) and satisfactory recyclability through either hot-pressing or solution casting enabled by the reversible DA crosslinking structure.

Xiang et al. [143] developed visible-light-driven polyurethane hydrogels that exploit a dual-network synergistic mechanism: HABI-based dynamic covalent bonds form reversible crosslinks, pentaerythritol-based permanent crosslinks form a stable framework, and hydrogen bonds in the urethane hard segments provide additional physical reinforcement. In this dual-covalently crosslinked PU gel, dynamic C–N bonds in HABI units serve as reversible photoresponsive crosslinks, enabling rapid underwater actuation within tens of seconds, while pentaerythritol permanent crosslinks maintain fundamental network structures and endow the material with excellent mechanical stability (ultimate stress of 5.18 MPa, strain of 579%, toughness of 12.58 MJ/m^3^ for 0.4 mm thick xerogel) and outstanding fatigue resistance (enduring 500 continuous fatigue cycles from 50% to 100% strain without failure), with the reversible photochromism demonstrating excellent reusability over multiple cycles.

In summary, the synergistic combination of hydrogen bonds and dynamic covalent bonds, through the three construction strategies of double-network interpenetration, single-network multiple crosslinking, and polyurethane hard-segment hydrogen bonding, realizes the synergistic mechanism of “fast bonds for energy dissipation, slow bonds for framework stabilization” across different spatial scales. Hydrogen bonds, owing to their ubiquity, directionality, and rapid reversibility, represent the most universal non-covalent module for combination with various types of dynamic covalent bonds. Their combinations with Schiff base bonds, disulfide bonds, boronate ester bonds, and other dynamic covalent bonds have already demonstrated the most mature and extensive application foundations in fields such as self-healing sensors, biolubrication coatings, and intelligent drug delivery.

### 3.2. Synergistic Integration of DCBs and Host–Guest Interaction

By covalently attaching host and guest molecules to different polymer chains while simultaneously incorporating reactive groups capable of forming dynamic covalent bonds along these chains, a dual crosslinking system that combines physical host–guest crosslinking with chemical dynamic covalent crosslinking can be obtained. The core synergistic logic lies in the following: host–guest inclusion complexes provide highly selective, reversible physical crosslinking points and orthogonal recognition capability, while dynamic covalent bonds supply the mechanical framework and chemical responsiveness, with the two independently or cooperatively regulating the network state under different stimulus conditions to achieve multi-modal orthogonal control. In hydrogel fabrication, different host–guest pairs are employed in different scenarios according to their dominant stimulus-response modes: the β-cyclodextrin/adamantane pair is primarily used for physiologically stable injectable hydrogels, the α-cyclodextrin/azobenzene pair for photo-controlled responsive hydrogels, the cucurbit[8]uril bis-guest system for high-strength and high-toughness hydrogels, and the β-cyclodextrin/ferrocene pair for electrochemically responsive conductive hydrogels [125].

β-Cyclodextrin and adamantane (β-CD/Ada) constitute the most widely applied host–guest pair. Their synergistic interplay through dynamic covalent bonds manifests as high physiological stability and injectability. The β-CD/Ada inclusion complex exhibits a binding constant of 104–105 M^−1^ at the physiological pH and temperature, ensuring that the hydrogel maintains network integrity in the in vivo environment. The role of dynamic covalent bonds in this synergistic system is reflected at three levels. First, they provide long-term structural stability and a controllable degradation rate, compensating for the potential long-term creep and network relaxation that may occur when host–guest crosslinking is used alone. Second, they endow the hydrogel with a chemically responsive dimension, enabling the mechanical properties and degradation behavior of the hydrogel to be regulated by pathological microenvironmental signals such as pH or redox conditions, forming orthogonal complementarity with the response of host–guest recognition to competitive guest molecules. Third, during self-healing, host–guest recognition drives rapid anchoring and alignment of the fractured surfaces at the molecular level, after which dynamic covalent bonds complete permanent covalent connections across the interface through exchange reactions. The two form a “fast recognition-slow repair” cascade, enabling the repaired hydrogel to recover higher mechanical strength. In drug delivery, the dissociation–reassociation equilibrium of host–guest inclusion complexes synergizes with the environmentally responsive cleavage of dynamic covalent bonds to achieve differential release behavior under multiple stimuli.

**Figure 5:**
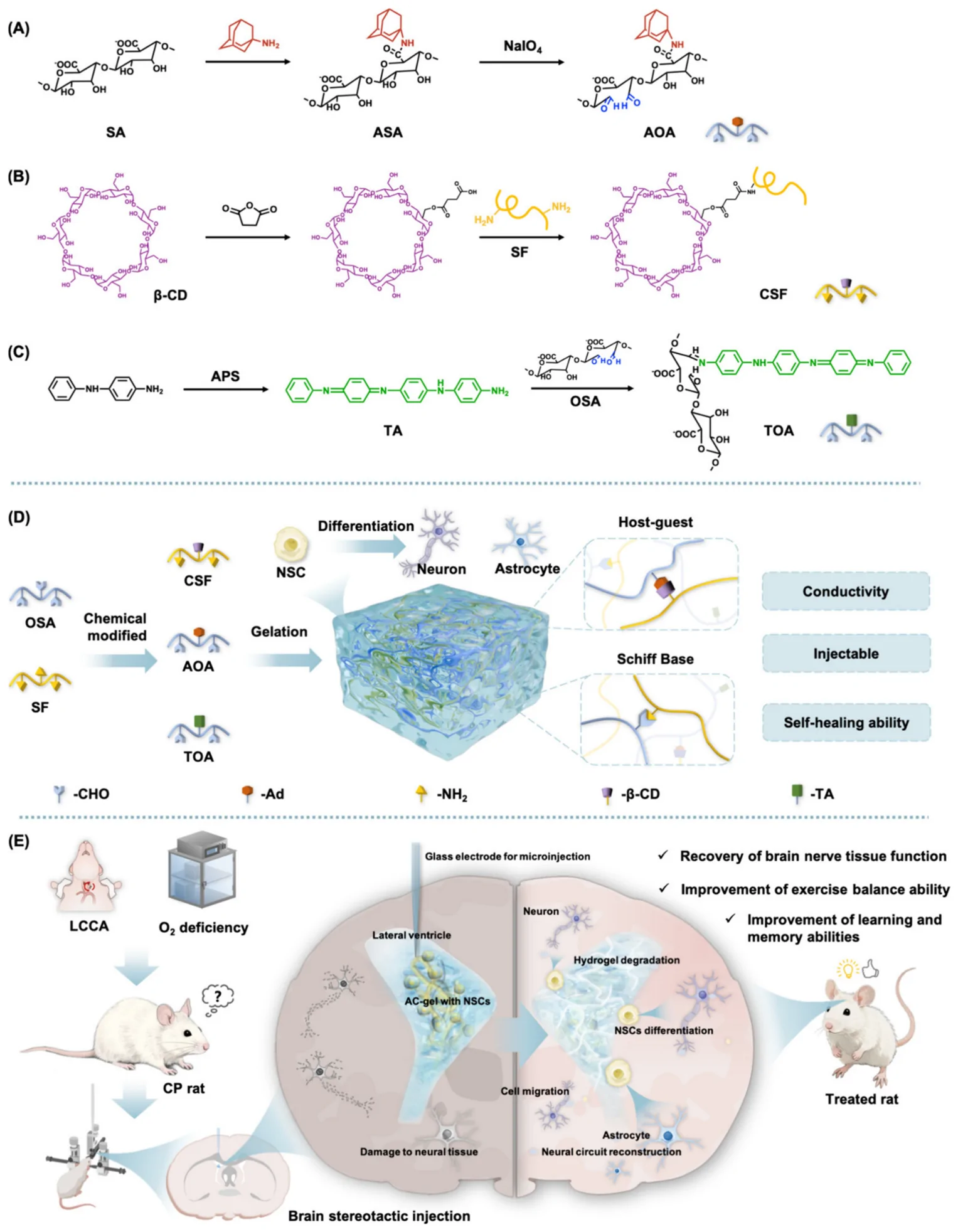
Synthesis, preparation and application of AC-gel, with all graphical components corresponding to the orthogonal, kinetically cascaded synergistic mechanism of host–guest and Schiff base dual dynamic crosslinks. (**A**–**C**) illustrate the synthetic routes of three functional macromolecular precursors: adamantane-modified oxidized alginate (AOA), β-cyclodextrin-grafted silk fibroin (CSF), and electroconductive tetrapolyaniline-functionalized alginate (TOA), which separately supply the guest moiety, host cavity, and conductive segments for network construction. (**D**) visualizes the formation of integrated hydrogel network: reversible pH-sensitive Schiff base dynamic covalent linkages form between oxidized alginate aldehyde and silk fibroin amine groups, while β-cyclodextrin/adamantane host–guest inclusion complexes act as independent physical crosslinks. The two types of dynamic interactions display orthogonal stimulus responsiveness and stepwise kinetic coordination: host–guest pairs enable rapid interfacial molecular recognition for instant surface anchoring upon damage, whereas Schiff bonds realize permanent network reconstruction via dynamic exchange, jointly supporting injectability and self-recovery. (**E**) depicts stereotactic intracranial implantation of NSC-laden AC-gel into cerebral palsy rat ventricles; the dual crosslinked network gradually degrades in vivo, and the coordinated host–guest/Schiff synergies sustain stable scaffold microenvironment to support NSC proliferation, differentiation and neural circuit reconstruction for brain functional restoration. Reprinted with permission from Ref. [144]. Copyright 2025, American Chemical Society.

For instance, Yu et al. [144] developed an injectable conductive hydrogel via a dual cross-linking synergistic mechanism—Schiff base formation between oxidized alginate and silk fibroin (SF), and host–guest interactions between β-cyclodextrin (β-CD) and adamantane—forming a dual-crosslinked network. In this alginate/SF-based hydrogel, Schiff base dynamic covalent bonds serve as pH-responsive, reversible crosslinks, imparting excellent self-healing capability and structural integrity during injection, while β-CD/Ada host–guest interactions resulting in high water content, a suitable degradation profile, and excellent biocompatibility for neural stem cell (NSC) encapsulation and delivery. Notably, the introduction of tetrapolyaniline-modified oxidized sodium alginate (TOA) as a conductive dopant significantly enhanced electrical conductivity (up to 0.16 ± 0.008 S/m) while maintaining high cytocompatibility, with NSC viability consistently exceeding 90% and robust cell adhesion (96.0 ± 2.5% retention). In a rat model of cerebral palsy, NSC-laden hydrogel transplanted via intracranial injection promoted significant structural repair and functional recovery, as confirmed by behavioral tests (1.70-fold increase in rotarod fall latency, *p* < 0.0001; 66.7% reduction in balance beam skid number), histology, and proteomics (upregulation of Bdnf by 1.6-fold, Mbp by 2.2-fold, and Cdc42 by 2.4-fold), indicating its potential therapeutic effect on the repair of cerebral tissue in CP (Figure 5). The injectable and conductive nature of this host–guest/DCB hydrogel makes it a promising platform for neural tissue engineering, particularly in the minimally invasive treatment of cerebral palsy and other neurodegenerative conditions. The key advantage lies in the synergistic combination of β-CD/Ada physical crosslinks for rapid self-healing post-injection and Schiff base covalent bonds for long-term structural stability in the brain microenvironment.

The β-CD/Ada host–guest pair provides excellent physiological stability and injectability, making it ideal for in vivo applications. However, its high binding constant (10^4^–10^5^ M^−1^) and insensitivity to external stimuli limit its utility in applications requiring spatiotemporal control over crosslinking density. To introduce an orthogonal stimulus-responsive dimension, the α-cyclodextrin and azobenzene (α-CD/Azo) pair offers a distinct advantage: the reversible trans-cis photoisomerization of azobenzene enables remote, non-invasive regulation of host–guest inclusion and dissociation. While the β-CD/Ada system responds to chemical signals through the competitive binding of guest molecules, the α-CD/Azo system responds to light, providing a completely orthogonal control mechanism. This photo-responsiveness allows the hydrogel to be modulated spatially and temporally with high precision, a capability that the chemically responsive β-CD/Ada system cannot achieve. The orthogonal synergy of the two pairs—one chemically responsive, the other photo-responsive—can be integrated into more sophisticated multi-responsive systems, as will be demonstrated in the following sections.

The synergistic core of the α-CD/Azo host–guest pair with dynamic covalent bonds lies in photo-controlled orthogonal responsiveness. Azobenzene undergoes reversible isomerization under UV/visible light irradiation, modulating its inclusion with and dissociation from cyclodextrin, thereby enabling remote optical control over the physical crosslinking density. Previous studies have validated the orthogonal synergistic principle of photo-controlled host–guest crosslinking and dynamic covalent bonds in vesicle systems—UV/visible light regulates the isomerization and inclusion state of azobenzene, while dynamic covalent bonds maintain structural integrity and provide a chemically responsive dimension, the two operating without mutual interference. This orthogonal regulatory mechanism is expected to be extended to hydrogel systems: in this synergistic system, dynamic covalent bonds first provide a light-independent mechanical framework. When optical signals trigger the dissociation of host–guest crosslinks, the dynamic covalent crosslinking maintains the overall structure of the hydrogel without complete disintegration, ensuring the reversibility and repeatability of the photo-controlled process. Second, the response of dynamic covalent bonds to chemical signals such as pH or temperature is completely orthogonal to the photo-responsiveness of the host–guest pair; the two types of signals independently manipulate the states of physical and chemical crosslinking without mutual interference, enabling the hydrogel to achieve stepwise regulation of mechanical properties under the dual stimuli of light and pH. Hydrogels based on the synergy of photo-controlled host–guest recognition and dynamic covalent bonds are still at an exploratory stage; however, the orthogonal response principle, already validated in vesicle systems, provides an important design basis for expanding this direction.

**Figure 6:**
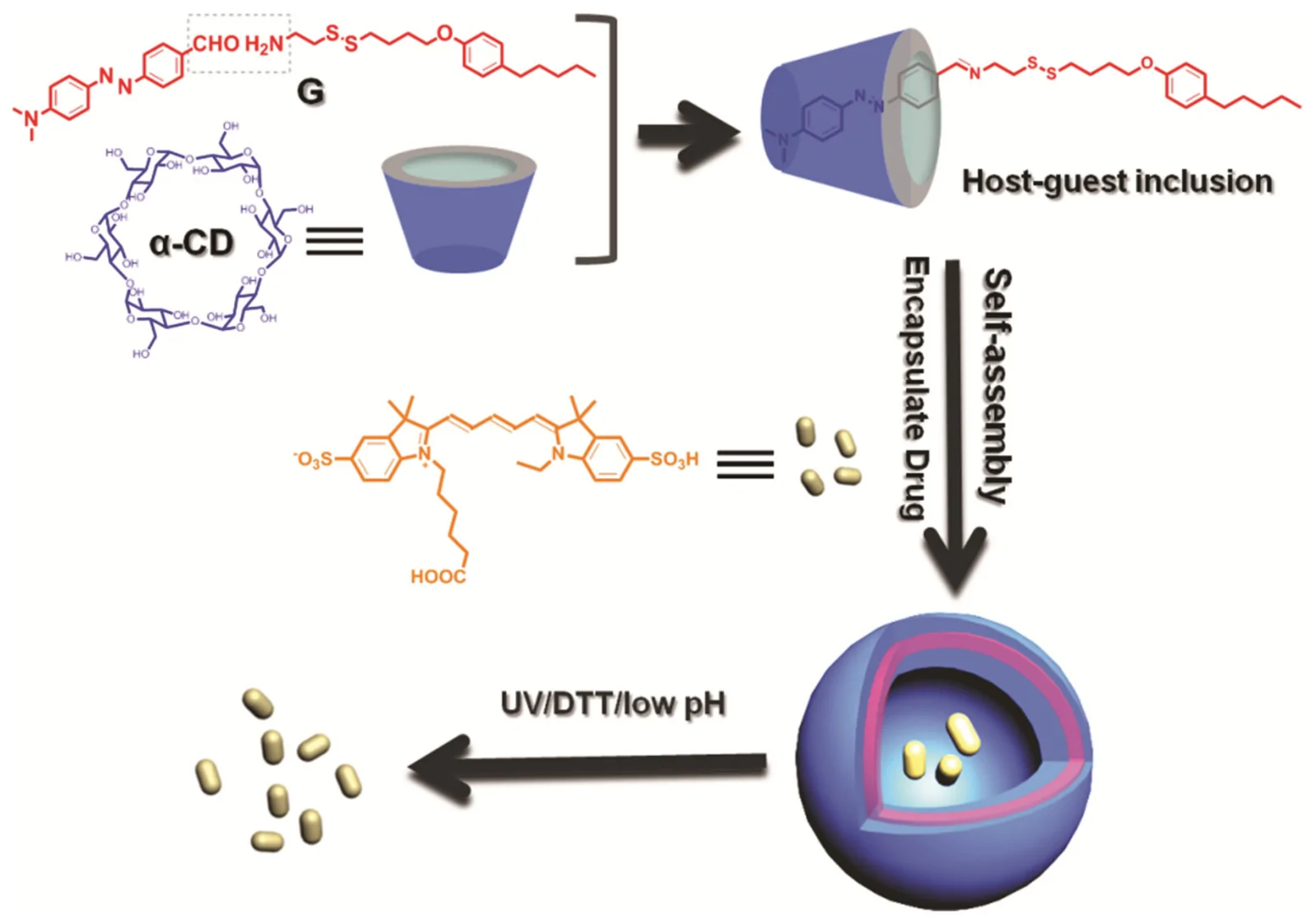
Schematic illustration of the construction and triple-stimuli-triggered disassembly process of orthogonal multi-dynamic supramolecular vesicles, with each graphical structural unit corresponding to the orthogonally responsive synergistic mechanism of host–guest inclusion and dual dynamic covalent bonds. The upper graphical segments visualize the molecular building blocks: α-cyclodextrin (α-CD) forms photo-switchable host–guest complexes with azobenzene (Azo) moieties on guest molecule G, while the same guest chain integrates pH-sensitive benzoic imine bonds and redox-responsive disulfide bonds as two independent dynamic covalent crosslinks. The three types of reversible interactions exhibit non-interfering, orthogonal stimulus responses, as shown in the diagram: α-CD/Azo host–guest pairs disassemble under UV light via azobenzene trans–cis isomerization; imine bonds cleave at acidic pH; disulfide bonds break upon exposure to the reductant DTT. These spatially coexisting yet independently responsive dynamic interactions jointly drive self-assembly into hollow vesicular carriers for cargo encapsulation, and separate stimulus pathways sequentially trigger vesicle dissociation and payload release, visually demonstrating the orthogonal, synergistic regulatory logic of photo-sensitive physical crosslinks and chemically responsive dynamic covalent bonds. Reprinted with permission from Ref. [126]. Copyright 2018, Royal Society of Chemistry.

For example, Li et al. [126] developed a triply responsive supramolecular vesicle that exploits a multi-dynamic synergistic mechanism—α-CD/Azo host–guest recognition, imine bonds, and disulfide bonds—forming a multi-responsive supramolecular assembly. In this α-CD/G vesicle system based on a guest molecule containing azobenzene, imine, and disulfide functionalities, imine and disulfide dynamic covalent bonds serve as pH- and redox-responsive chemical linkages respectively, imparting controlled release (up to 60% dye release within 12 h upon acidification or DTT addition), while α-CD/Azo host–guest inclusion (association constant of 2.73 × 10^4^ M^−1^) acts as a photo-responsive physical crosslink, enabling UV-triggered vesicle disassembly through trans-cis isomerization of azobenzene, thereby achieving triple responsiveness to UV irradiation, pH change (7.5), and redox reagent (DTT) for the controlled release of encapsulated payloads (Figure 6).

While the α-CD/Azo pair successfully introduced photo-responsiveness, it relies on a 1:1 host–guest stoichiometry, which limits the energy dissipation capacity of a single crosslinking point to a single dissociation event. The cucurbit[8]uril (CB[8]) ternary inclusion complex offers a fundamentally different mechanism: its large cavity simultaneously accommodates two guest molecules, forming a charge-transfer-stabilized 1:2 ternary complex in which two independently tunable dynamic interfaces coexist at a single crosslinking point. This unique feature enables hierarchical energy dissipation—under external force, the two guests can dissociate sequentially, providing two-tiered sacrificial bond behavior within a single crosslinking junction. Dynamic covalent bonds further complement this system by serving as the ultimate framework that maintains network integrity. The introduction of CB[8] thus elevates the host–guest system from a binary “on-off” switch to a multi-level energy management platform, bringing the mechanical behavior of the hydrogel closer to the hierarchical energy management mechanisms found in natural biomaterials such as bone and spider silk.

The synergistic interplay between cucurbit[8]uril (CB[8]) 1:2 ternary inclusion complexes with bis-guests and dynamic covalent bonds lies in hierarchical energy dissipation. The large cavity of CB[8] simultaneously accommodates one electron-rich and one electron-deficient guest molecule, forming a charge-transfer-stabilized ternary inclusion complex in which two independently tunable dynamic interfaces coexist at a single crosslinking point. The role of dynamic covalent bonds in this system is to serve as the ultimate framework that maintains network integrity, constituting a three-level energy dissipation system together with the two kinetic modes of ternary complex dissociation. Under external force, the ternary inclusion complex is the first to dissociate, acting as the first-tier sacrificial bond; the sequential dissociation of the two guests provides second-tier intermediate-rate energy dissipation; dynamic covalent bonds consistently maintain the macroscopic shape of the network and ultimately repair residual damage through slow exchange. The introduction of dynamic covalent bonds elevates the host–guest system from purely “physical crosslinking” to “physical-chemical hierarchical crosslinking,” bringing the mechanical behavior of the hydrogel closer to the hierarchical energy management mechanisms of natural biomaterials.

**Figure 7:**
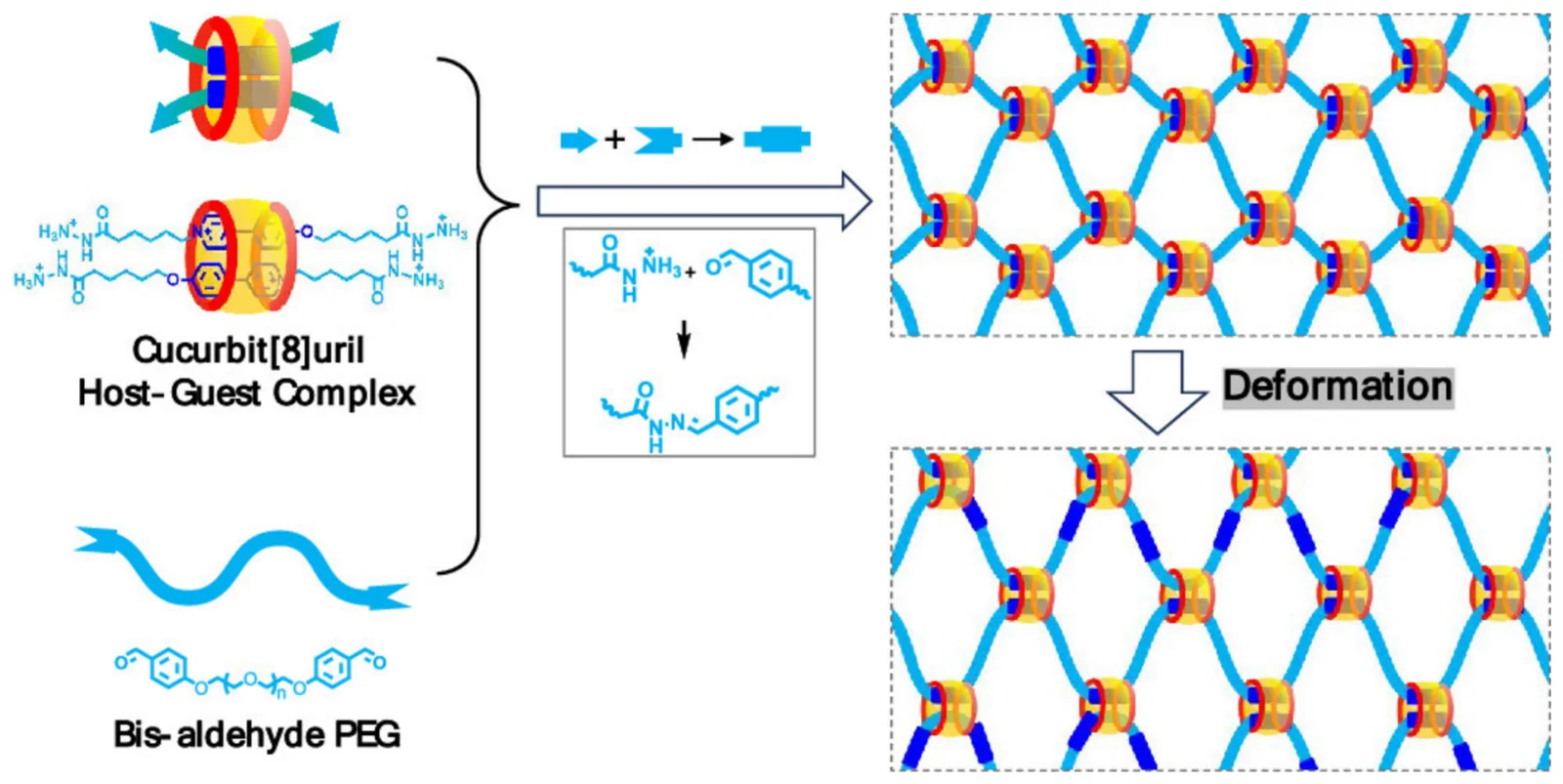
Schematic graphical abstract illustrating the fabrication and deformation-dependent hierarchical energy dissipation mechanism of dual-crosslinked PEG hydrogels with movable CB[8] supramolecular cores. The left graphical segments display two building units: CB[8] 1:2 ternary host–guest homoternary complexes serving movable tetravalent supramolecular crosslink cores, and linear bis-aldehyde PEG chains that connect the cores via pH-sensitive acylhydrazone dynamic covalent bonds. The middle reaction schematic visualizes the co-assembly of the two dynamic interactions into an analog four-arm PEG network, realizing hierarchical physical–chemical dual crosslinking. The upper undeformed network diagram and lower stretched structure visually present the kinetically cascaded synergistic behavior: movable CB[8] ternary complexes act as fast sacrificial sites for primary energy dissipation and slide along polymer chains under tension to homogenize the network, while acylhydrazone dynamic covalent linkages serve as permanent structural skeletons, preserving overall network topology and enabling slow post-strain repair via bond exchange. This graphic directly maps the multi-tier energy dissipation cascade of host–guest ternary complexes and dynamic covalent bonds described in the synergistic mechanism. Reprinted with permission from Ref. [127]. Copyright 2025, American Chemical Society.

For example, Zou et al. [127] developed dynamic hydrogels via a hierarchical dual-crosslinking synergistic mechanism—movable CB[8] host–guest ternary inclusion complexes as supramolecular cores and acylhydrazone dynamic covalent bonds forming a 4-arm PEG analog network. In this PEG-based hydrogel with CB[8]–(BPG)_2_ homoternary complexes (K_eq_ ≈ 10^13^ M^−2^) as movable tetravalent cores and bis-aldehyde PEG crosslinkers, acylhydrazone dynamic covalent bonds serve as pH-responsive structural crosslinks that maintain network integrity, while CB[8]–guest ternary complexes act as movable supramolecular cores that can slide along polymer chains under deformation. The hydrogel exhibited exceptional mechanical properties, with the BPG20 formulation achieving unprecedented stretchability of up to 7000% elongation at break—far exceeding conventional 4-arm PEG hydrogels—and rate-dependent mechanical behavior: slow deformation rates (250 mm/min) enabled 7000% elongation, whereas fast rates (1000 mm/min) yielded increased Young’s modulus (from ~20 kPa to ~50 kPa) and decreased toughness, demonstrating that supramolecular core dynamics govern the macroscopic mechanical response. Rheological characterization revealed the frequency-dependent Maxwell behavior of dynamic viscoelastic hydrogels, with the characteristic bulk relaxation time (*τ*_R_) of BPG10 calculated as 6.6 s, compared to 19.6 s for the fixed-core CTR10 hydrogel and 0.3 s for the more dynamic AZG10 system, indicating that the association–dissociation kinetics of CB[8]–guest complexes directly modulate network dynamics. The hydrogels also exhibited exceptional shear-strain tolerance with linear viscoelastic regimes up to 500% strain and critical yield strains exceeding 2000%, far surpassing conventional dynamic networks crosslinked exclusively by either supramolecular host–guest interactions or acylhydrazone bonds. The movable CB[8] cores enable network homogenization under slow deformation, engaging more load-bearing polymer chains and leading to enhanced macroscopic modulus and toughness, representing a significant advancement in hydrogel engineering (Figure 7). The unprecedented extensibility (~7000%) and rate-dependent mechanics of this CB[8]-based system open new avenues for soft robotics and tough hydrogel actuators. However, the reliance on competitive displacement for network modulation may limit its responsiveness in complex biological environments where competing guest molecules are present.

The CB[8] system achieves remarkable mechanical reinforcement through its ternary inclusion geometry and hierarchical energy dissipation, yet its responsiveness relies primarily on competitive displacement by chemical agents, which may be slow or irreversible in practice. For applications requiring rapid, reversible, and cyclable modulation of crosslinking density, the β-cyclodextrin and ferrocene (β-CD/Fc) pair offers an electrochemically controlled alternative. The reversible redox reaction of ferrocene—oxidation to ferrocenium and reduction back to ferrocene—enables electrode-potential-controlled inclusion and exclusion from the β-CD cavity within seconds, with excellent cyclability. This electrochemical responsiveness is fundamentally different from the chemical or photo-responsiveness of the preceding systems, offering a distinct control modality that can be integrated with electronic devices. Furthermore, the intrinsic charge-transfer capability of the ferrocene/ferrocenium redox couple provides additional functionality beyond crosslinking regulation, including electrical conductivity and electrochemical sensing, making the β-CD/Fc system particularly suitable for applications in flexible bioelectronics and electro-controlled drug delivery systems.

The synergistic core of the β-CD/Fc host–guest pair with dynamic covalent bonds lies in the integration of electrochemically controllable crosslinking and conductive functionality. Reduced ferrocene is encapsulated within cyclodextrin; upon electrochemical oxidation, it dissociates from the cavity, and upon reduction, it is re-encapsulated, thereby achieving reversible control of the physical crosslinking state through electrode potential. The role of dynamic covalent bonds in this system is to maintain the structural integrity of the network during electrochemical cycling—repeated redox cycles may cause transient dissociation and reassociation of host–guest crosslinks—and the dynamic covalent framework ensures that the hydrogel does not undergo macroscopic disintegration during this process. Second, dynamic covalent bonds provide an independent response dimension beyond electrochemical signals, enabling the hydrogel to simultaneously respond to both electrical and chemical signals (e.g., pH or redox conditions). Electrical signals remotely regulate the host–guest crosslinking density and ion transport rate, while chemical signals trigger structural changes in the dynamic covalent network. The synergy of the two is well-suited to scenarios requiring dual-mode electrochemical regulation, such as electrically controlled drug release and flexible bioelectrodes.

**Figure 8:**
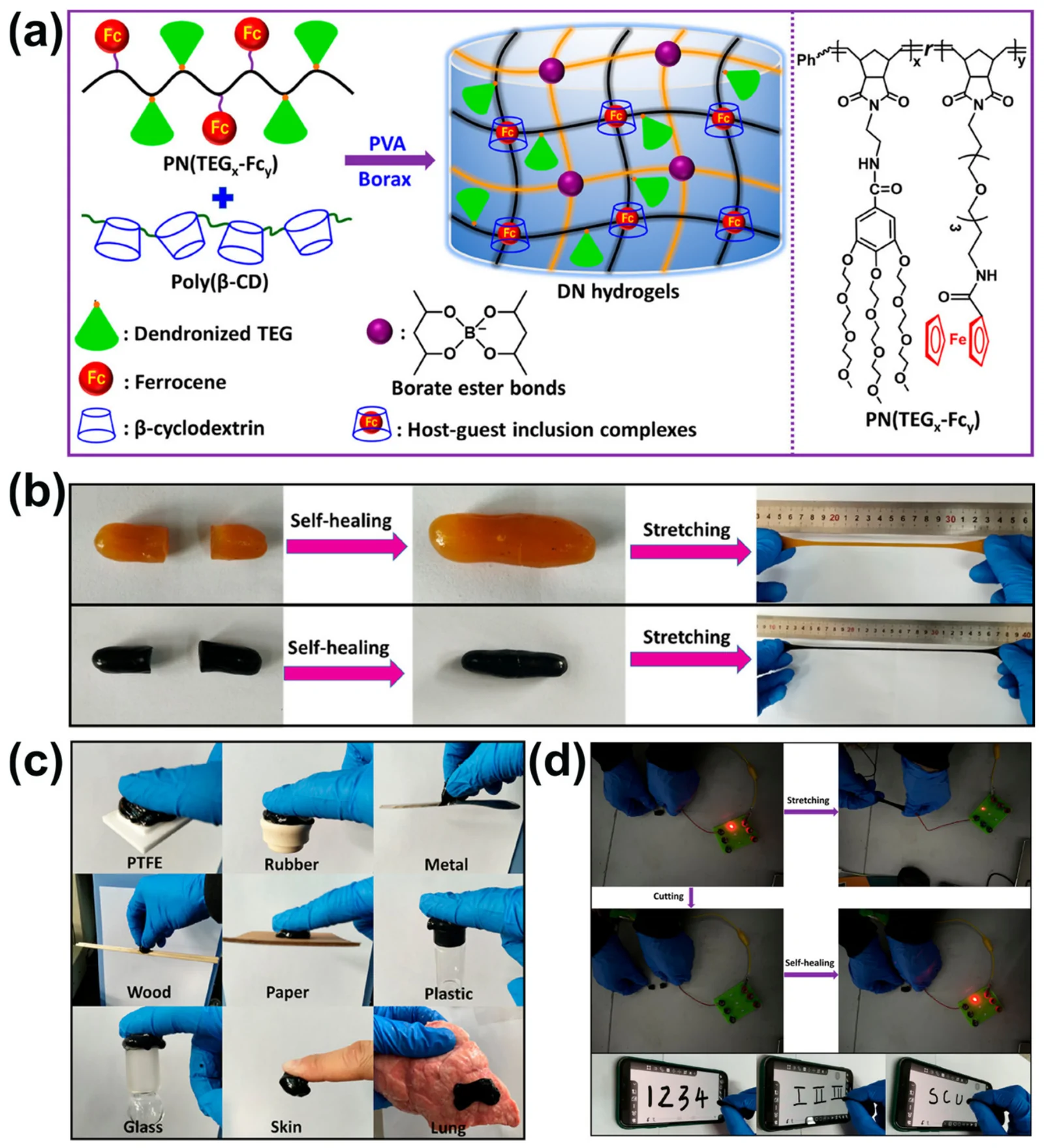
Composite schematic and characterizations of dual-dynamic interpenetrating DN hydrogel built from orthogonal β-cyclodextrin/ferrocene (β-CD/Fc) host–guest physical crosslinks and borate ester dynamic covalent bonds, with each panel visually corresponding to the electrochemically and chemically dual-responsive synergistic mechanism. (**a**) Molecular assembly schematic illustrating two kinetically distinct crosslinking motifs coexisting in one interpenetrating network: reversible borate ester dynamic covalent bonds act as permanent structural skeletons to sustain macroscopic integrity during redox cycling, while β-CD/Fc host–guest inclusion complexes serve as electro-switchable physical crosslinks whose association/dissociation can be remotely tuned by electrochemical signals, realizing orthogonal electro-chemical dual regulation as described in the mechanism. (**b**) Photographs of cut-and-recovered DN hydrogel showing the kinetically cascaded self-healing behavior enabled by the two dynamic interactions: fast re-association of host–guest pairs rapidly bonds fracture surfaces, followed by gradual reformation of borate ester bonds to recover full mechanical performance. (**c**) Optical images demonstrating broad substrate adhesion derived from the integrated dual crosslink network, where the combined host–guest and borate ester interactions collectively strengthen interfacial binding with diverse materials including biological pig tissue. (**d**) Demonstrations of conductive functional applications originating from the orthogonal synergistic design: the intact dual crosslink framework maintains stable conductive pathways after damage, allowing the healed hydrogel to form complete circuits and serve as touch-sensitive wearable writing electrodes. Adapted with permission from Ref. [145]. Copyright 2021, American Chemical Society.

For instance, Liu et al. [145] developed a multifunctional self-healing dual network hydrogel that exploits a dual dynamic synergistic mechanism—β-CD/Fc host–guest interactions and dynamic borate ester bonds forming an interpenetrating double network. In this PVA/PN(TEG-Fc)/Poly(β-CD) DN composite hydrogel with carbon nanotubes, borate ester dynamic covalent bonds serve as reversible crosslinks, imparting rapid room-temperature self-healing capability (healing efficiency of 95.3% within 5 min, with healed fracture stress of 39.1 kPa and fracture strain of 408%), while β-CD/Fc host–guest interactions act as physical crosslinks that enhance network cohesion and structural stability, achieving good stretchability (fracture strain of 436%), high fracture strength (41.0 kPa), excellent adhesion strength (11.1, 11.4, 7.6, and 5.6 kPa toward nitrile glove, plastic, rubber and pig skin), high tensile strain sensitivity (gauge factor of 5.9) and good electrical conductivity for monitoring various human motions and organ movements (Figure 8). The electrochemical responsiveness of the β-CD/Fc pair, combined with the mechanical robustness of boronate ester crosslinks, makes this hydrogel well-suited for self-healing wearable electronics and human–machine interfaces. The rapid healing efficiency (95.3% within 5 min) ensures device durability, while the strain sensitivity (GF = 5.9) enables precise motion detection.

To provide a quantitative perspective on the distinct characteristics of the four host–guest pairs discussed above, we summarize their binding affinities, stimulus triggers, and response timescales in Table 2. This comparison highlights the trade-offs between binding strength and responsiveness: high-affinity pairs such as β-CD/Ada and CB[8]/bis-guest offer thermodynamic stability but slower or less reversible responses, whereas lower-affinity pairs such as α-CD/Azo and β-CD/Fc enable rapid and cyclable modulation through external stimuli.

In summary, the synergistic combination of host–guest recognition with dynamic covalent bonds, through the selection of different host–guest pairs—from the physiologically stable β-CD/Ada, the photo-controlled α-CD/Azo, and the high-strength and high-toughness CB[8] bis-guest system, to the electrochemically responsive β-CD/Fc—coupled with appropriate dynamic covalent crosslinking chemistry, enables the modular customization of crosslinking kinetics, stimuli-response modes, and mechanical properties. Host–guest recognition endows the hydrogel with molecular-level precise recognition and orthogonal regulatory capability, while dynamic covalent bonds provide the mechanical framework and multidimensional chemical responsiveness. The synergistic integration of the two forms a hydrogel design platform that currently comes closest to the concept of “programmable soft materials”.

### 3.3. Synergistic Integration of DCBs and Metal–Ligand Interaction

Integrating metal–ligand physical crosslinking with dynamic covalent bonds follows a core synergistic logic: metal–ligand bonds serve as tunable sacrificial bonds that undertake energy dissipation or stimuli-responsive functions, while dynamic covalent bonds maintain the structural integrity and elastic recovery of the network, the two complementing each other in the dimensions of mechanical reinforcement, stimuli-responsiveness, and biological functionality. In hydrogel fabrication, according to the desired functional orientation, the systems can be classified into three categories: high-valence ions such as Ca^2+^, Al^3+^, and Fe^3+^ are employed for mechanically reinforced hydrogels; Ca^2+^-carboxyl, Fe^3+^-catechol, and Zn^2+^-carboxyl coordination are utilized for stimuli-responsive hydrogels; and bioactive ions such as Zn^2+^, Sr^2+^, and Na^+^ are used for bioactive hydrogels.

Mechanically reinforced metal coordination represents one of the most intensively investigated strategies within this direction. Metal ions such as Fe^3+^, Ag^+^, Cu^2+^, Zn^2+^, and Ni^2+^ form reversible crosslinking points through coordination with catechol, terpyridine, or carboxylate ligands, and are frequently combined with dynamic covalent networks such as Schiff base bonds, disulfide bonds, or transient anhydride bonds. The synergistic core of the two lies in kinetic complementarity—metal–ligand bonds possess relatively fast exchange rates with tunable dynamics depending on the metal ion species, capable of completing cleavage and reformation on the timescale of seconds to minutes, whereas dynamic covalent bonds exhibit comparatively slow exchange rates, requiring extended periods to complete bond reorganization. This fast–slow kinetic disparity manifests as orthogonal contributions during self-healing: after external force-induced damage, the dynamic metal coordination bonds, by virtue of their rapid re-association capability, are the first to re-form at the fractured surfaces, providing immediate structural recovery and re-establishing network integrity; subsequently, the slower dynamic covalent bonds complete exchange and network rearrangement, providing long-term stabilization of the three-dimensional framework. The orthogonal synergy between metal coordination and dynamic covalent bonds also endows the hydrogel with precise viscoelastic and mechanical tunability—the exchange rate and lifetime of coordination bonds can be modulated by varying the metal ion species (e.g., Zn^2+^ forming moderately dynamic gels, Cu^2+^ yielding highly dynamic soft materials, and Ni^2+^ forming robust stable networks), while the crosslinking density and stability of the covalent network can be regulated by adjusting the content of covalent crosslinkers or the type of dynamic covalent chemistry, the two being independently adjustable, thereby offering a highly flexible design space for the on-demand customization of mechanical properties, self-healing performance, and functional integration.

For example, Rajawasam et al. [124] developed a programmable hydrogel system based on a dual dynamic network combining persistent metal–ligand coordination and transient anhydride bonds. In this poly(DMAm-AA-tPy) hydrogel system, divalent metal ions (Ni^2+^, Zn^2+^, Cu^2+^) form reversible crosslinking points through coordination with terpyridine ligands, while the polymer backbone contains carboxylic acid groups that can be transiently crosslinked via EDC-fueled anhydride formation. The metal–ligand coordination bonds exhibit tunable exchange rates depending on the metal ion species: Cu^2+^ undergoes rapid ligand exchange on the timescale of ~0.1 s, yielding highly dynamic, soft materials; Zn^2+^ exchanges on the timescale of ~10 s, forming moderately dynamic gels; and Ni^2+^ exhibits exchange times exceeding 100 s, creating robust, stable networks. This metal-dependent kinetic tunability enables precise control over network dynamics and mechanical properties at the resting state. Upon EDC fueling, transient anhydride bonds are formed, temporarily increasing crosslink density and boosting the storage modulus (G′) significantly—with ΔG′ reaching 17 kPa for Ni^2+^-crosslinked materials and 11 kPa for Zn^2+^ and Cu^2+^ systems, representing increases of approximately 3- to 5-fold over resting values. The transient reinforcement lasts up to 280 min depending on the metal ion, after which the system relaxes back to its resting state through anhydride hydrolysis. The persistent metal–ligand coordination and transient anhydride bonds operate orthogonally and synergistically: metal coordination governs long-term self-healing and sustained network integrity, while transient anhydrides provide temporary mechanical reinforcement that can be activated on demand. Self-healing experiments demonstrated independent contributions from both dynamic mechanisms—EDC fueling temporarily boosted peak stress recovery by 300% (Zn^2+^) and 400% (Ni^2+^) at 1 h, while metal coordination ensured sustained recovery over time, with Ni^2+^-based materials achieving complete (100%) recovery of both stress and strain after 24 h. Tensile testing confirmed that EDC fueling substantially enhances mechanical performance, with peak stress increasing 6-fold (from 60 ± 15 kPa to 420 ± 30 kPa) for Zn^2+^ hydrogels and 9-fold (from 14 ± 2 kPa to 120 ± 10 kPa) for Ni^2+^ hydrogels compared to the unfueled state. The orthogonal synergy between metal–ligand coordination and transient anhydride bonds also endows the hydrogel with precise viscoelastic and mechanical tunability—the exchange rate and lifetime of coordination bonds can be modulated by varying the metal ion species (Cu^2+^ yielding highly dynamic soft materials, Zn^2+^ forming moderately dynamic gels, and Ni^2+^ creating robust stable networks), while transient crosslink density can be independently regulated through EDC fueling, offering a highly flexible design space for on-demand customization of mechanical properties and self-healing performance.

**Figure 9:**
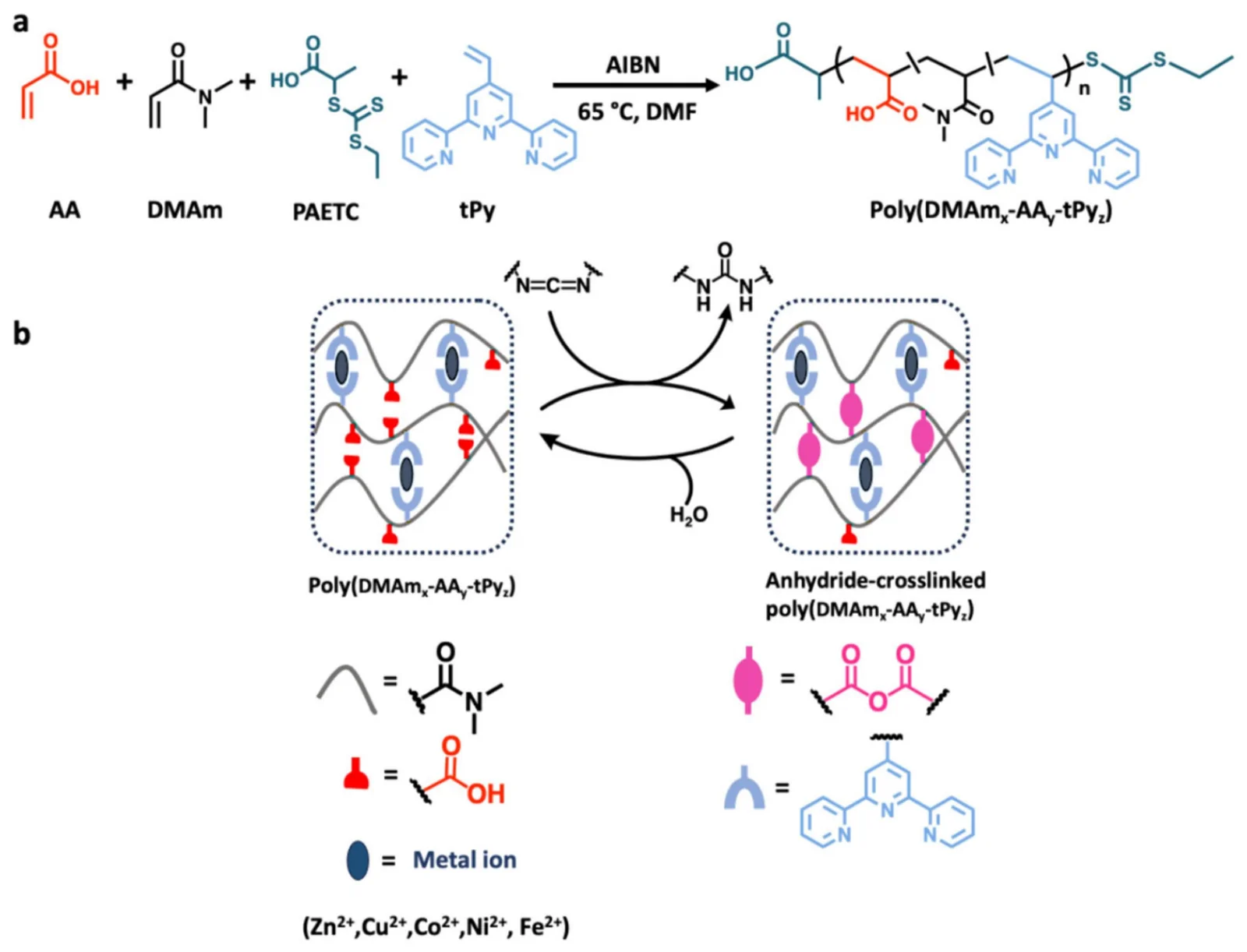
Synthetic and crosslinking schematic of dual-dynamic poly(DMAm-AA-tPy) hydrogel with orthogonally operating persistent metal-terpyridine coordination bonds and transient anhydride dynamic covalent bonds, with each graphical module corresponding to the kinetically differentiated synergistic mechanism of two independent dynamic crosslinks. (**a**) Synthetic schematic of terpyridine-functionalized poly(DMAm_x_-AA_y_-tPy_z_) precursor polymer, which contains terpyridine ligand side chains for persistent metal–ligand coordination and pendant carboxylic acid groups reserved for fuel-triggered transient crosslinking, laying the structural foundation for orthogonal dual dynamic networks. (**b**) Schematic overview of the dual crosslinking synergistic system: divalent metal ions coordinate with terpyridine motifs to form persistent dynamic crosslinks with metal-tunable ligand exchange kinetics, while EDC chemical fuel reacts with backbone carboxyl groups to generate temporary anhydride dynamic bonds. The diagram visually distinguishes two kinetically independent crosslinking modes with orthogonal functional roles: metal-terpyridine coordination acts as a long-lived persistent framework to sustain baseline network integrity and long-term self-repair, whereas fuel-induced anhydride bonds serve as transient dynamic crosslinks that temporarily elevate crosslink density for on-demand mechanical reinforcement, fully demonstrating the fast–slow kinetic complementary and non-interfering orthogonal synergistic logic. Reprinted with permission from Ref. [124].

Furthermore, taking advantage of the programmable and reversible nature of this dual dynamic system, spatially controlled EDC treatment of Zn^2+^ and Cu^2+^ hydrogel films enabled the generation of complex, rewritable 2D stiffness patterns with three distinct modulus levels (<10 kPa for soft Cu^2+^ regions, 20–60 kPa for medium Zn^2+^ regions, and 60–90 kPa for EDC-treated high-modulus regions), which could be erased and rewritten by allowing the system to relax and reapplying the fuel, demonstrating exceptional programmability and reusability. This hydrogel system, featuring independent tunability of persistent and transient dynamic bonds, combines on-demand mechanical reinforcement, robust self-healing, and spatiotemporal programmability, with broad application prospects in soft robotics, tissue scaffolding, adaptive materials, and reusable mechanical patterning (Figure 9). The programmable stiffness patterns and orthogonal dynamic bond tunability of this system are particularly valuable for adaptive materials and reusable mechanical patterning. However, the use of EDC as a chemical fuel for transient reinforcement raises concerns about biocompatibility and in vivo applicability, as reaction byproducts may induce local inflammatory responses.

Du et al. [146] developed a mechanically reinforced hydrogel with a dual-network structure constructed from Al^3+^-polyphenol metal–ligand interactions and boronate ester dynamic covalent bonds. In this Al^3+^-TA/GG-PAM hydrogel system, Al^3+^, as a typical high-valence metal ion, forms high-density crosslinking points through multidentate coordination. The metal–ligand bonds exhibit fast exchange rates, capable of cleaving and reforming within seconds, not only significantly enhancing the overall strength and toughness of the gel—achieving an ultra-high tensile strain of 839%—but also enabling the first reconstruction upon damage, rapidly anchoring and apposing the fractured interfaces. Boronate esters, as dynamic covalent bonds, possess relatively slow bond exchange rates, gradually undergoing exchange reactions after metal coordination has accomplished interfacial apposition, thereby providing long-term stabilization of the three-dimensional network framework. The two form a typical fast–slow kinetic cascade self-healing mechanism. The metal–ligand coordination and boronate ester bonds exhibit orthogonal synergistic characteristics: the lifetime of coordination bonds and the stability of the covalent network can be separately regulated by changing the metal ion species and switching the type of dynamic covalent bond, enabling on-demand customization of the viscoelasticity, mechanical properties, and self-healing capability of the hydrogel. Cyclic tensile tests demonstrated that the material possesses excellent fatigue resistance and deformation recovery capability. Furthermore, the hydrogel relies on free aluminum ions to form ion-conductive pathways, and combined with the broad-spectrum adhesion imparted by polyphenol groups, achieves a gauge factor of 1.87, enabling stable monitoring of various motion signals including human joint activities and facial micro-expressions, providing an excellent design concept and application solution for high-performance mechanically reinforced flexible sensing hydrogels.

Li et al. [147] developed a medical adhesive hydrogel based on Fe^3+^-catechol metal–ligand interactions and a dynamic covalent Schiff base bonds, forming a dual-dynamic network. In the PL-Cat/Fe/ODex hydrogel system, Fe^3+^, as a typical high-valence metal ion, forms a multidentate coordination structure with catechol groups, thereby forming a high-density network of crosslinking points. The metal–ligand bonds exhibit fast exchange rates, capable of cleaving and reforming within seconds, effectively enhancing the rigidity, compressive strength, and structural stability of the gel framework, while also being the first to re-associate at the fractured surfaces upon damage, rapidly apposing the fractured interfaces. Schiff base bonds, as dynamic covalent bonds, exchanging after metal coordination establishes interfacial anchoring, thereby providing long-term stabilization of the three-dimensional network. The two form a typical fast–slow kinetic cascade self-healing mechanism. The metal coordination and Schiff base bonds exhibit orthogonal synergistic characteristics, allowing separate regulation of coordination bond lifetime and covalent network stability, enabling on-demand control of the mechanical properties and self-healing capability of the hydrogel. The compressive strength of the hydrogel reached 80 kPa, with a swelling ratio significantly lower than that of single-bond crosslinked systems. Water-assisted self-healing was achieved via dual-dynamic bonds, with the compressive strength recovering to 91% of the initial value after healing. Moreover, the hydrogel possesses injectability and pH/chelating agent-responsive dissolution characteristics. Porcine skin lap shear tests demonstrated an adhesive strength of up to 15 kPa, with high adhesion force retained after repeated bonding. Cell and in vivo tissue experiments confirmed the excellent biocompatibility of the material, which can be applied to skin incision closure and post-operative wound care, providing an excellent design strategy for wound repair-oriented mechanically reinforced hydrogels.

Despite these advances, the metal coordination in mechanically reinforced systems is primarily exploited for its mechanical contribution, with limited attention paid to its intrinsic sensitivity to environmental signals. Metal–ligand bonds are inherently responsive to changes in pH, redox conditions, temperature, and the presence of competing ligands or metal ions—a sensitivity that can be harnessed to impart stimuli-responsive functionality to the hydrogel. The transition from mechanically reinforced to stimuli-responsive systems thus represents a shift in design philosophy: rather than simply using metal–ligand bonds as “strong but reversible” crosslinks, the latter approach exploits their environmental sensitivity as a functional output. This paradigm shift extends the kinetic complementarity principle from the domain of self-healing to the domain of environmental responsiveness.

Stimuli-responsive metal coordination exploits the sensitivity of metal–ligand bonds to environmental signals, forming orthogonal complementarity with the chemical responsiveness of dynamic covalent bonds. While metal coordination offers rapid, reversible responses to physical/chemical stimuli (e.g., pH, redox, or competing ions), dynamic covalent bonds provide an independent chemically responsive channel with distinct kinetics. The functional complementarity between the two bond types manifests across multiple levels. (i) In mechanical regulation, when a given stimulus simultaneously triggers cleavage of dynamic covalent bonds and dissociation of metal coordination, the superimposed response can produce order-of-magnitude changes in gel modulus—far exceeding the modulation range of either bond type alone. (ii) In sol–gel transitions, pH changes can synchronously trigger dynamic covalent bond hydrolysis and protonation-induced weakening of coordinating groups. This synergistically achieves reversible gel–sol transitions while endowing the hydrogel with injectability and self-healing capability. (iii) In photo-controlled systems, visible light can selectively drive dynamic covalent bond exchange, while metal coordination maintains network structural integrity during the process. This avoids photothermal damage to surrounding tissues. The rapid reversibility of metal–ligand bonds endows the hydrogel with injectability and rapid self-healing, while the chemical responsiveness of dynamic covalent bonds imparts environmental sensing and adaptive degradation functions. The combination of the two enables such hydrogels to exhibit multifunctional integrated application potential in biomedical fields including in vivo drug delivery, inflamed/infected wound repair, and wearable materials.

**Figure 10:**
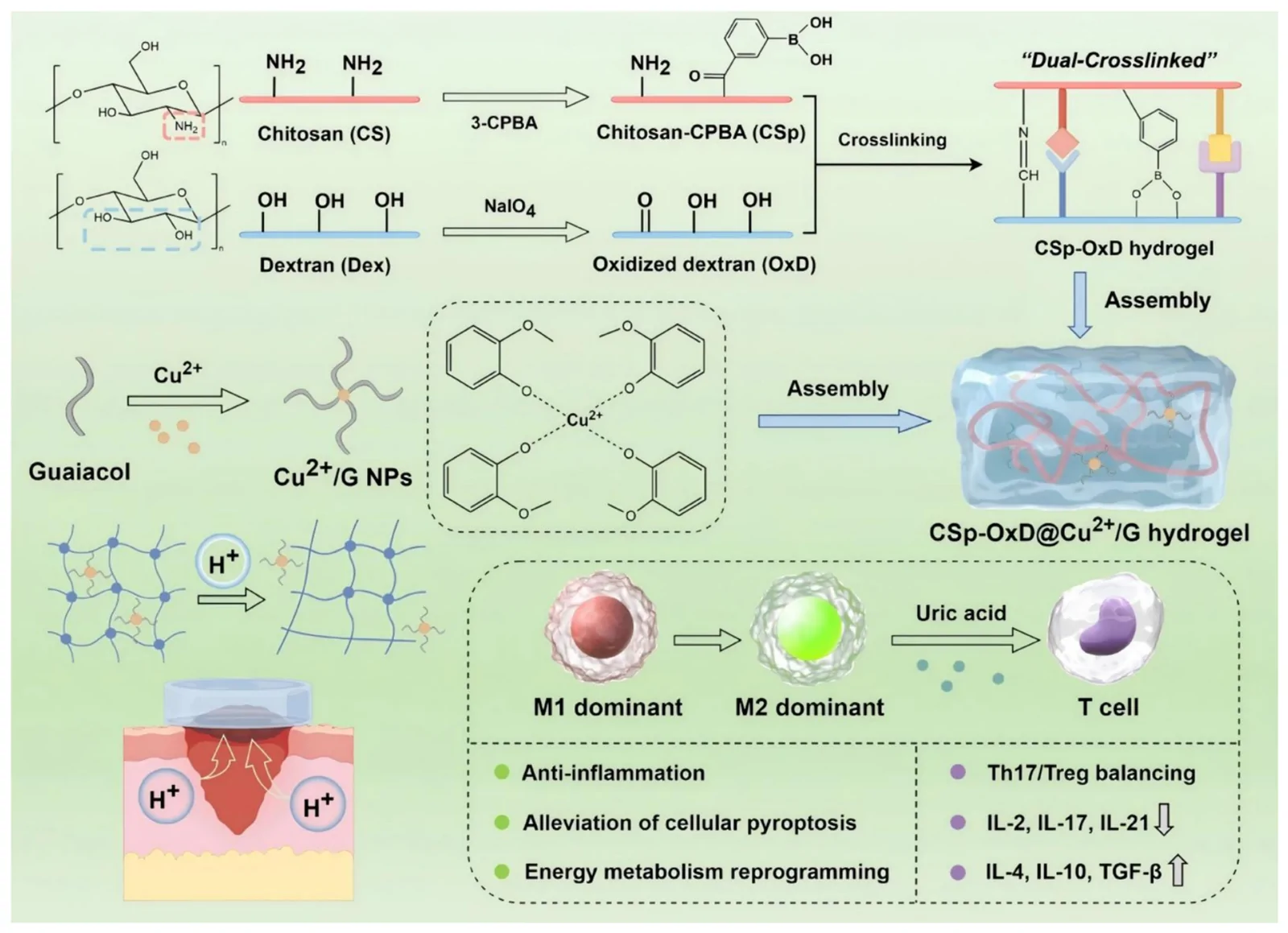
Schematic diagram of CSp-OxD@Cu^2+^/G hydrogel fabrication, pH-responsive degradation and diabetic wound therapeutic workflow, in which visualized structural units (circles, dots and arrows) correspond to the orthogonally responsive dual dynamic synergistic mechanism described in the text. The upper synthesis panel graphically distinguishes two kinetically differentiated dynamic covalent crosslinks within one unified network: Schiff base bonds formed between chitosan amino groups and oxidized dextran aldehydes, and borate ester linkages generated from boronic acid and polyol segments. Blue dots in the network denote cross-linking nodes, while orange dots represent embedded Cu^2+^/guaiacol metal-coordination nanoparticles acting as a third reversible supramolecular component. The diagram explicitly illustrates the orthogonal functional division of these dynamic interactions: pH-labile borate ester bonds undergo preferential dissociation in the acidic diabetic wound microenvironment (where light-blue circled H^+^ symbols visualize local proton enrichment) to trigger sustained release of Cu^2+^/G nanoparticles; curved arrows depict substance diffusion and release, while stable Schiff base dynamic covalent skeletons remain intact to preserve the overall network architecture, compensating for temporary crosslink loss. The middle microenvironment-responsive schematic visualizes the kinetically complementary cascade: fast-reversible Cu^2+^-phenolic metal coordination imparts instant injectability and self-repair upon recontact, whereas slower pH-triggered borate ester hydrolysis realizes long-term tunable payload delivery. The bottom in vivo therapeutic panel further presents the downstream immunomodulatory outcomes enabled by this orthogonal bond synergy, including M1-to-M2 macrophage reprogramming and balanced Th17/Treg adaptive immunity for accelerated diabetic wound regeneration. Up-pointing arrows mark up-regulated biological factors and down-pointing arrows mark down-regulated ones; different color blocks across panels distinguish hydrogel network domains and stratified skin-tissue layers, directly mapping the multi-level functional complementarity of metal coordination and dual dynamic covalent bonds. Reprinted with permission from Ref. [148].

For example, Liao et al. [148] developed a stimuli-responsive multifunctional hydrogel based on the synergistic construction of Schiff base and borate ester dynamic covalent bonds, combined with Cu^2+^-phenolic metal coordination interactions. In this CSp-OxD@Cu^2+^/G hydrogel system, the dynamic covalent bonds—Schiff base linkages formed between amino groups of carboxyphenylboronic acid-grafted chitosan (CSp) and aldehyde groups of oxidized dextran (OxD), together with borate ester bonds formed between boronic acid groups and hydroxyl groups—constitute a dual-crosslinked network that provides structural stability, injectability, and self-healing capability. The metal coordination, realized through Cu^2+^/G nanoparticles generated via chelation of guaiacol with copper ions, serves as a reservoir for therapeutic Cu^2+^ and guaiacol, while the borate ester bonds exhibit high sensitivity to pH changes, responding to the mildly acidic microenvironment characteristic of diabetic wounds. The synergy between the two bond types manifests as orthogonal complementarity in controlled drug release: the borate ester bonds gradually dissociate in response to H^+^ in the local environment (pH 5.5), triggering sustained release of Cu^2+^/G nanoparticles, while the Schiff base network maintains the fundamental structural integrity of the hydrogel system, compensating for the loss of physical support upon borate bond dissociation. This pH-responsive degradation behavior is quantitatively demonstrated by rapid degradation rates reaching 68% and 85% on days 3 and 6, respectively, at pH 5, whereas degradation is markedly slower in neutral and mildly alkaline environments (~67% by day 6). The Cu^2+^/G nanoparticle release exhibits a pH-dependent profile, with significantly higher Cu^2+^ concentrations at pH 5 than at neutral conditions, confirming the pH-triggered dissociation mechanism. The rapid reversibility of metal coordination endows the hydrogel with excellent injectability and self-healing capability (G′ > G″ consistently across all concentrations, with step-strain tests confirming rapid structural recovery), while the chemical responsiveness of dynamic covalent bonds imparts environmental sensing and pH-dependent degradation functions. By virtue of this dual-crosslinked architecture, the hydrogel exhibits outstanding comprehensive performance: superior compressive strength (withstanding pressures nearing 150 kPa and enduring 80% deformation without structural failure), good biocompatibility (live/dead staining showing no significant cytotoxicity against HUVECs and RAW264.7 cells, with CCK8 assay confirming excellent cytocompatibility), and effective hemostatic capability comparable to positive controls in a mouse liver hemorrhage model. Notably, the hydrogel system demonstrates potent immunomodulatory effects: it effectively reprograms macrophages towards the M2 anti-inflammatory phenotype, inhibits LPS-induced pyroptosis (as evidenced by substantial reductions in NLRP3, cleaved-caspase-1, cleaved-IL-1β, and cleaved-GSDMD expression), restores glycolytic energy metabolism homeostasis, and indirectly regulates Th17/Treg balance in CD4^+^ T cells via macrophage-mediated signaling. Metabolomics analysis further reveals that uric acid may serve as a key metabolite in macrophage-CD4^+^ T cell crosstalk. In vivo studies in a diabetic mouse model demonstrate significantly accelerated wound healing (markedly reduced wound remaining rate by day 14 compared to control groups), enhanced epithelial regeneration (substantially greater epithelial thickness), and increased collagen deposition, while effectively restoring local immune homeostasis through coordinated regulation of both innate (macrophage polarization) and adaptive (Th17/Treg balance) immunity. This composite system integrates pH-responsive drug release, injectability, self-healing, mechanical robustness, hemostatic capability, broad-spectrum immunomodulation, and wound healing promotion, possessing exceptionally high application value in the fields of chronic diabetic wound management, immune microenvironment remodeling, and multifunctional smart wound dressings (Figure 10). This hydrogel is specifically engineered for the complex pathological microenvironment of diabetic wounds, where pH-responsive drug release, broad-spectrum immunomodulation, and hemostatic properties are required. The Cu^2+^ coordination serves a dual role—providing both mechanical crosslinking and therapeutic ion release—exemplifying the principle of structure–function integration. A critical consideration for clinical translation is the potential cytotoxicity of copper ions at high local concentrations, which necessitates precise control over release kinetics.

Liang et al. [149] developed a stimuli-responsive multifunctional hydrogel based on the synergistic construction of Fe^3+^-catechol metal–ligand interactions and dynamic Schiff base covalent bonds. In this system, Fe^3+^-catechol coordination bonds exhibit high sensitivity to environmental signals such as pH, redox conditions, and competing ions (e.g., deferoxamine mesylate, DFO), constructing the first rapid response dimension, while dynamic Schiff base bonds formed between aldehyde groups of protocatechualdehyde (PA) and amino groups of quaternized chitosan (QCS) provide an independent chemically responsive channel with distinct kinetics. The two types of bonds can be triggered individually or act synergistically in response to external stimuli. At the mechanical regulation level, pH changes or DFO intervention simultaneously induce Fe^3+^ coordination dissociation and Schiff base hydrolysis, with the dual action superimposed to achieve substantial modulus reduction—the adhesive strength dramatically decreases from 10.4 kPa to 1.3 kPa upon DFO treatment, enabling on-demand removal, while the gelation time can be tuned from 50 min to 10 min with increasing crosslinking density. The storage modulus (G′) increases from 270 Pa to 1980 Pa, demonstrating the wide mechanical tunability far exceeding that of single dynamic bond systems. In terms of sol–gel transition, pH changes can synchronously trigger Schiff base hydrolysis and protonation-induced weakening of catechol-Fe coordination, synergistically achieving controlled dissolution and endowing the material with excellent injectability and shear-thinning behavior (continuous injection through a syringe to draw “XJTU” patterns without clogging). In photo-controlled systems, the catechol-Fe coordination complex serves as a photothermal agent, enabling NIR-responsive photothermal antibacterial therapy, while the Schiff base network maintains the structural integrity of the hydrogel, preventing photothermal damage to surrounding normal tissues. The rapid reversibility of Fe^3+^-catechol coordination bonds endows the hydrogel with fast autonomous self-healing capability (complete healing within 1–2 h without any external stimuli, crack-free and capable of bearing its own weight, with rheological G′ and G″ recovering to near-original values even after five alternating high–low-strain cycles), while the chemical responsiveness of Schiff base bonds imparts pH-dependent degradation and environmentally adaptive dissolution functions, the two dynamic bonds forming favorable complementarity. By virtue of the dual-dynamic-bond crosslinked network, the hydrogel exhibits outstanding multifunctional performance: remarkable antibacterial activity against *E. coli* (>90% inactivation within 2 h), *S. aureus* (~80%), and MRSA (up to 98% inactivation, with NIR-assisted treatment enabling complete pathogen elimination), excellent antioxidant capacity (~90% DPPH radical scavenging), strong tissue adhesion (adhesive strength up to 40 kPa on porcine skin in a PA@Fe content-dependent manner, with the optimized formulation achieving 10.4 kPa and repeated wound closure capability with >60% recovery after healing for 1 h), robust hemostatic performance (significantly reduced blood loss from 485 mg to 279 mg in tail amputation model, and from 426 mg to 102 mg in liver hemorrhage model), good cytocompatibility (L929 cell viability exceeding 100% after 3 days of incubation, live/dead staining showing predominantly live cells), and excellent in vivo biocompatibility (mild inflammatory response that gradually subsided as the hydrogel degraded over 4 weeks). In vivo evaluation in a rat skin incision model demonstrated superior wound closure effectiveness compared to surgical sutures and commercial biomedical glue, with higher tensile strength of healed skin and scarless healing. In an MRSA-infected full-thickness skin wound model, the NIR-assisted hydrogel achieved nearly complete wound contraction. It significantly accelerated healing (complete re-epithelialization by day 21) with increased collagen deposition, enhanced neovascularization (higher CD31 expression), and reduced inflammation (lower CD68 expression) compared to control groups. This composite system integrates pH/NIR stimulus-responsiveness, on-demand removability, injectability, autonomous self-healing, mechanical robustness, strong tissue adhesion, antibacterial activity, antioxidant capacity, hemostatic capability, and wound-healing promotion, offering exceptionally high application value in sutureless wound closure, infected wound dressings, and post-wound-closure care.

Xu et al. [150] developed a stimuli-responsive soybean protein-based double dynamic crosslinked hydrogel based on Fe^3+^-galloyl metal–ligand coordination and Schiff base dynamic covalent bonds for the controlled delivery of curcumin. In this system, Fe^3+^-galloyl coordination bonds exhibit high sensitivity to pH changes, constructing the first chemically responsive dimension, while Schiff base bonds formed between aldehyde groups of oxidized dextran (ODeX) and amino groups of gallic acid-grafted soy protein isolate (SPA) provide an independent pH-responsive channel with distinct kinetics. The two types of bonds can be triggered individually or act synergistically in response to external pH stimulation. At the mechanical regulation level, the dual crosslinking architecture enables the hydrogel to withstand 50% compressive strain with significantly enhanced mechanical strength—SPD8@Fe1 exhibits a compression stress of 26.86 kPa, hardness of 213.44 g, cohesiveness of 0.953, and chewiness of 172.11 mJ, representing 4.3-, 4.3-, 1.8-, and 5.2-fold improvements over the single-crosslinked SPA hydrogel, demonstrating that the superimposed reinforcement of Schiff base and metal coordination far exceeds the modulation range of either bond type alone. In terms of sol–gel transition and injectability, pH changes synchronously trigger Schiff base hydrolysis and protonation-induced weakening of Fe^3+^-galloyl coordination, synergistically achieving pH-responsive gel degradation and drug release—the SPD8@Fe1 hydrogel exhibits only 26.22% curcumin release at pH 2.0 versus 33.44% at pH 7.0 after 28 h, with the higher release in acidic medium attributed to imine protonation and coordination bond dissociation. The rapid reversibility of Fe^3+^-galloyl coordination bonds endows the hydrogel with excellent self-healing capability (G′ and G″ recovering to near-original values under alternating high–low-strain cycles), while the chemical responsiveness of Schiff base bonds imparts pH-dependent degradation and controlled release functions, the two dynamic bonds forming favorable complementarity. By virtue of the double crosslinked network, the hydrogel exhibits outstanding comprehensive performance: optimal thermal stability (30.69% residual mass at 600 °C), excellent water retention (highest proportion of bound and immobilized water), low swelling ratio (45.44 g/g), and superior bile salt adsorption capacity, confirming that the synergistic effect of coordination bonds and Schiff bases significantly enhances the gel network. In vitro gastrointestinal digestion simulations demonstrate that the hydrogels can withstand gastric environment-induced damage and sustain curcumin release under intestinal conditions, with drug release following Fick’s law (R^2^ > 0.97) and the release kinetics regulated by adjusting the dynamic bond content. This composite system integrates pH-responsive controlled release, self-healing capability, mechanical robustness, thermal stability, water retention, and bile salt adsorption, offering high application value in functional food delivery systems and oral bioactive ingredient protection.

The stimuli-responsive systems discussed above exploit metal–ligand bonds primarily as sensors that translate environmental signals into mechanical or structural changes. However, metal ions are not merely structural or signaling elements—many of them possess intrinsic biological activities. Zn^2+^, Sr^2+^, Mg^2+^, and Cu^2+^, among others, play essential roles in enzymatic catalysis, cell signaling, tissue regeneration, and antibacterial defense. The integration of these bioactive metal ions with dynamic covalent networks represents a further evolution of the design philosophy: metal–ligand bonds now serve simultaneously as mechanical nodes, responsive crosslinks, and bioactive ion reservoirs. In this configuration, the degradation or exchange rate of dynamic covalent bonds directly regulates the dissociation rate of the coordination network, thereby controlling the release profile of bioactive ions. In pathological microenvironments such as infected or inflamed tissues, the responsive cleavage of dynamic covalent bonds accelerates network disintegration, prompting the synchronous dissociation of metal coordination bonds and the release of bioactive ions—a stimulus-response–ion-release cascade amplification effect. This synergy transforms the hydrogel from a passive structural material into an active therapeutic platform that senses pathological signals, responds accordingly, and delivers therapeutic ions in a spatiotemporally controlled manner. The bioactive systems represent the highest level of functional integration among the metal-coordination strategies, combining mechanical reinforcement, stimuli-responsiveness, and therapeutic functionality within a single network.

Bioactive metal coordination integrates the biological functions of metal ions with dynamic covalent networks, constructing hydrogels that combine pro-healing and catalytic therapeutic functions in an integrated manner. The coordination crosslinking of bioactive ions such as Zn^2+^, Sr^2+^, and Na^+^ serves simultaneously as mechanical nodes and ion reservoirs. The exchange or degradation rate of dynamic covalent bonds directly regulates the dissociation rate of the coordination network, thereby controlling the release profile of metal ions. In pathological microenvironments, the responsive cleavage of dynamic covalent bonds accelerates network disintegration, with coordination bonds subsequently dissociating and releasing bioactive ions, thereby amplifying a stimuli-response–ion-release cascade. This synergy transforms the hydrogel from a passive carrier into an intelligent drug release platform capable of sensing and responding to pathological signals.

**Figure 11:**
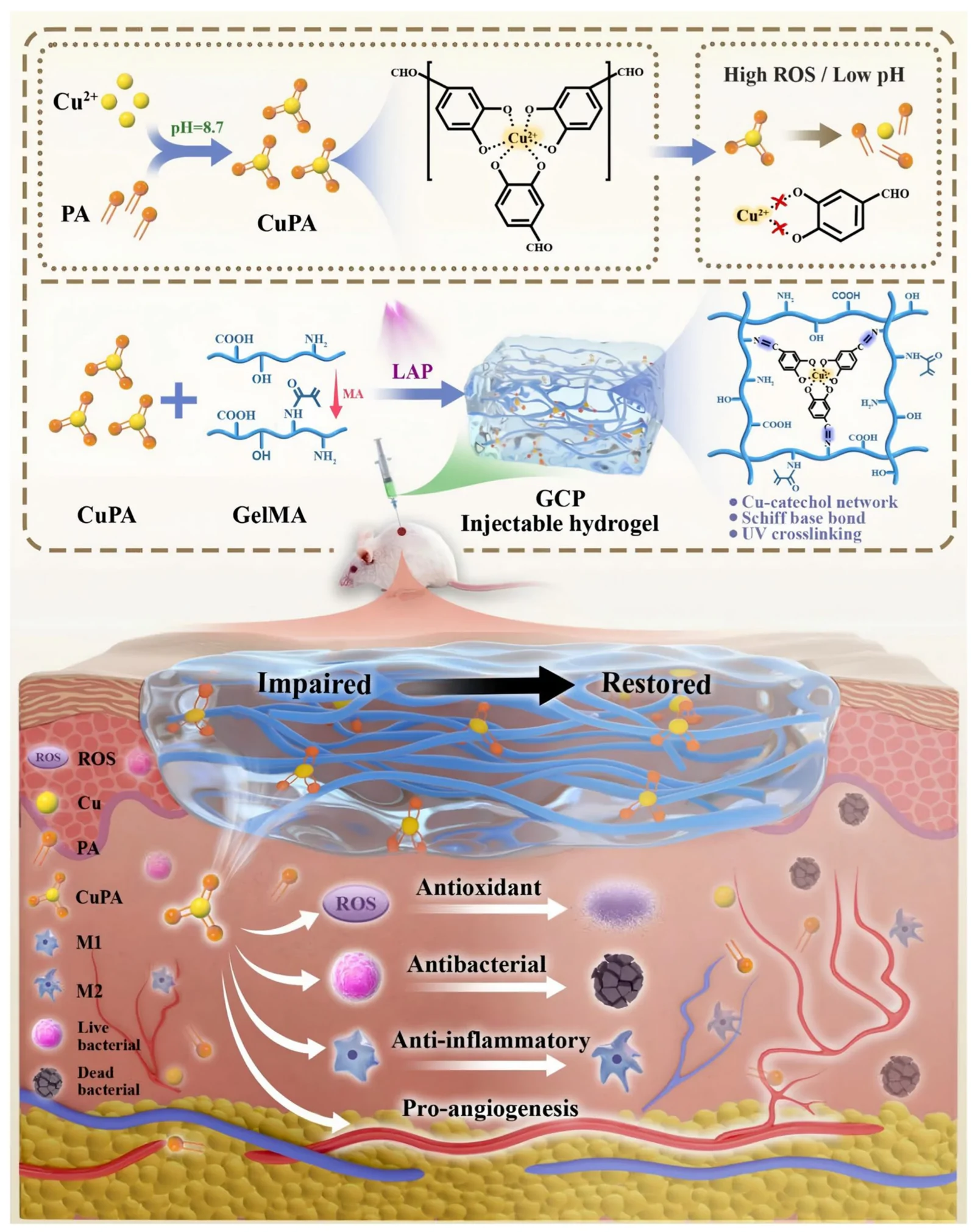
Schematic overview illustrating the fabrication of multi-crosslinked GCP hydrogel and its cascade-responsive therapeutic mechanism, with each visualized structural unit directly mapped to the synergistic cascade coordination of metal coordination and Schiff base dynamic bonds. The top molecular assembly panel graphically distinguishes two core reversible crosslink motifs coexisting within the photo-crosslinked GelMA backbone: Cu^2+^-catechol metal–polyphenol coordination complexes act as dual-functional crosslink nodes that simultaneously sustain network mechanics and store bioactive Cu^2+^/PA payloads, while pH-sensitive Schiff base dynamic covalent linkages form orthogonal responsive skeletons that govern the overall degradation kinetics. Red cross marks in the molecular sketches denote cleavage sites for reversible coordination and covalent bonds. The middle network schematic visualizes the kinetically cascaded stimuli-response mechanism captured in the illustration: under the acidic pathological microenvironment of diabetic wounds, proton-triggered cleavage of Schiff bonds occurs first to loosen the integral hydrogel framework, which sequentially accelerates dissociation of Cu–PA coordination aggregates and triggers synchronous release of bioactive components, realizing the stimuli–ion-release cascade amplification effect shown in the graphic. The bottom in vivo wound panel further depicts the downstream tissue repair outcomes enabled by this dual-bond synergistic cascade, including antibacterial, antioxidative and M2 macrophage polarization effects driven by the sequentially released Cu^2+^ and PA molecules, fully demonstrating the spatiotemporal coordinated responsiveness of metal coordination and dynamic covalent bonds. Reprinted with permission from Ref. [151].

For example, Zhang et al. [151] developed a multiple-crosslinked injectable bioactive hydrogel integrating Cu^2+^-polyphenol metal coordination with Schiff base dynamic covalent bonds, achieving integrated antibacterial, antioxidant, angiogenic, and anti-inflammatory functions for infected diabetic wound repair. In this GelMA/CuPA composite hydrogel, the coordination structure formed between Cu^2+^ and catechol groups of protocatechualdehyde (PA) serves simultaneously as physical crosslinking nodes constructing the gel framework to maintain basic mechanical properties and as a reservoir of bioactive ions. Schiff base bonds, formed between the aldehyde groups of CuPA and the amino groups of GelMA, serve as dynamic covalent bonds whose pH-responsive cleavage directly regulates the dissociation rate of the Cu-catechol coordination network, thereby achieving controlled release of Cu^2+^ and PA in the acidic diabetic wound microenvironment. In the pathological wound microenvironment characterized by bacterial infection, elevated oxidative stress, and reduced pH, the Schiff base bonds undergo responsive cleavage upon protonation, accelerating overall network disintegration and prompting the synchronous dissociation of Cu-catechol coordination bonds with the release of Cu^2+^ and PA, forming a stimuli–response–ion-release cascade amplification effect. The hydrogel exhibits outstanding antibacterial activity against both *S. aureus* and *E. coli* (inhibition rate ~98%), with 16S rRNA sequencing revealing that CuPA complexes downregulate ABC transporter genes (modA, B4602_RS12435, B4602_RS03205, etc.) and aminoacyl-tRNA biosynthesis genes (aspS, trpS, serS) while upregulating oxidoreductase activity to hinder bacterial protein synthesis. The GCP hydrogel demonstrates exceptional antioxidant properties (significant O_2_^−^ and DPPH radical scavenging, catalase-like activity for H_2_O_2_ decomposition), combined with excellent injectability, self-healing (reversible Schiff base and Cu-catechol coordination bonds), tissue adhesion (effective adhesion to heart, liver, spleen, and kidney), hemostatic capability (bleeding reduced to 60.53 ± 10.60 mg in rat liver injury model), good biocompatibility (hemolysis rate 0.87%, cell viability >90%), and remarkable pro-angiogenic activity (upregulation of VEGF, CD31, and α-SMA expression, promoting macrophage polarization toward M2 phenotype). In vivo studies in a rat infected diabetic wound model demonstrate significantly accelerated wound healing with reduced epithelial gap, increased collagen density, and enhanced formation of hair follicles and sebaceous glands, outperforming both GelMA and GC (ceria nanoparticle-containing) hydrogel controls. This multiple-crosslinked hydrogel system, combining Cu^2+^ bioactive metal coordination with Schiff base dynamic covalent bonds and photo-crosslinked GelMA networks, provides an intelligent platform for sensing and responding to pathological signals, offering a promising strategy for infected diabetic wound management (Figure 11). The integration of Cu^2+^ bioactivity with Schiff base dynamic crosslinks makes this hydrogel particularly suited for infected wound beds, where antibacterial activity, angiogenesis promotion, and oxidative stress alleviation are simultaneously needed. The pH-responsive cleavage of Schiff base bonds ensures on-demand ion release in the acidic wound microenvironment. However, the long-term metabolic fate of the degradation products and the potential for systemic copper accumulation require careful evaluation.

Duan et al. [152] developed a bioactive integrated hydrogel constructed from Sr^2+^ metal–ligand interactions and Schiff base bonds. This system organically combines the biological functions of strontium ions with a dynamic covalent network to achieve integrated therapy encompassing antibacterial and anti-inflammatory effects, immunomodulation, promotion of angiogenesis, and bone repair. In the C/O/Sr/MA composite hydrogel system, the metal coordination structure formed between Sr^2+^ and polyphenol groups serves both as high-density physical crosslinking nodes constructing the three-dimensional gel framework to ensure basic mechanical properties (compressive strength of 19.88 ± 2.51 kPa, adhesion strength of 29.03 ± 0.62 kPa) and as a reservoir of bioactive ions, enabling functional ion loading and sustained release (cumulative Sr^2+^ release of 55.76% over 9 days). The exchange and degradation rates of Schiff base dynamic covalent bonds can precisely regulate the disintegration rate of the overall network, thereby achieving controllable modulation of Sr^2+^ release behavior, with appropriate degradation profiles (66.26% residual weight after 12 days) and favorable swelling capacity (130.28%). In the pathological microenvironment of infected, inflammation-rich bone defects, Schiff base bonds are triggered by pathological signals to undergo responsive cleavage, accelerating the gradual disintegration of the gel network and prompting the synchronous dissociation of Sr^2+^ coordination bonds with the release of bioactive strontium ions, forming a stimulus-response–ion-release cascade amplification effect. This system completely breaks free from the limitation of traditional dressings as merely passive carriers, actively sensing pathological environmental changes and responding accordingly. Relying on the sustained release of Sr^2+^ in synergy with components such as chlorogenic acid, it inhibits the proliferation of pathogenic bacteria such as MRSA (antibacterial rate of 99.98%), regulates macrophage polarization toward the M2 phenotype (56.83% M2 macrophage proportion vs. 0.19% in LPS group), alleviates local inflammation (significantly reduced TNF-α expression), and simultaneously effectively promotes angiogenesis (VEGF expression enhancement, tube formation branch count of 46.33 vs. 22.33 in control), osteoblast differentiation, and collagen deposition, comprehensively driving the repair of infected bone defects. Tests demonstrated that the hydrogel possesses excellent injectability (injectable through a 26G needle), tissue adhesion, shape adaptability, and self-healing capability. Blood and cell experiments confirmed its favorable biocompatibility (cell viability >85%, hemolysis rate <5%), and in vivo animal models also verified its outstanding bone repair efficacy (BV/TV of 57.94% at week 7). At present, research on hydrogels combining such bioactive ions with dynamic covalent bonds remains at an early stage overall. This study achieved multiple therapeutic functions with a single coordination crosslinking system, also providing important insights and expansion directions for the subsequent design of dual dynamic bond synergistic systems and the development of multifunctional intelligent materials for bone repair. This Sr^2+^-coordinated system is specifically designed for infected bone defect repair, where the combination of antibacterial activity (99.98% against MRSA), immunomodulation, and osteogenic promotion is essential. The sustained release of Sr^2+^ from the Schiff base-crosslinked network provides long-term therapeutic ion delivery. However, the relatively low mechanical strength (~20 kPa compressive) may limit its application in load-bearing bone sites.

Fu et al. [153] developed a bioactive, multifunctional hydrogel constructed from Na^+^-metal–ligand interactions and Schiff base dynamic covalent bonds. This system combines the biological characteristics of sodium ions with a dynamic covalent network, balancing multiple properties including low-temperature environmental adaptability, conductive sensing, and biocompatibility to achieve the integrated construction of flexible bio-devices. In this gelatin/cellulose-based composite hydrogel system, the metal coordination structure formed between Na^+^ and carboxyl groups on the polymer chains serves both as high-density physical crosslinking points constructing a robust three-dimensional network, significantly enhancing the tensile strength and toughness of the hydrogel, and as an ion reservoir ensuring the ionic conductivity of the system. Schiff base bonds, as dynamic covalent bonds, have their exchange and degradation rates capable of regulating the overall state of the network, the two forming a functionally complementary dual crosslinking structure. Meanwhile, under special external environments such as low temperatures, the Na^+^ coordination structure can inhibit ice crystal formation, cooperating with the Schiff base dynamic network to maintain the intact morphology and mechanical properties of the hydrogel. By leveraging the responsiveness of dynamic bonds, reversible adjustment of the network structure can be achieved, thereby, forming a synergistic effect of environmental adaptation and structural stability. The hydrogel, derived from biomass, possesses excellent biocompatibility and degradability. Na^+^ endows the system with stable ionic conductivity, enabling it to serve as a flexible strain sensor that precisely recognizes various human motion signals. Simultaneously, sodium ion coordination, in synergy with multiple dynamic bond interactions, allows the gel to retain more than half of its mechanical properties in low-temperature environments, breaking through the limitation of low-temperature failure inherent to traditional hydrogels. Tests demonstrated that the tensile strength of the hydrogel increased by 38-fold (reaching 6.4 MPa) and toughness increased by 39-fold (reaching 2.9 MJ/m^3^) through the triple dynamic bond strategy, with excellent fatigue resistance under cyclic deformation, outstanding water retention capability after encapsulation (only 4.8% water loss after 168 h), and stable operation even in sub-zero low-temperature environments (retaining 50% tensile strength and 68% toughness at −60 °C, with reliable sensing performance at −20 °C and −4 °C). At present, research on composite hydrogels combining bioactive ions with dynamic covalent bonds is still in the development stage. This work, employing Na^+^ coordination combined with Schiff base bonds to construct a triple dynamic bond network, provides a new feasible approach for the design and application of low-temperature flexible bio-devices and wearable sensing materials. This Na^+^-coordinated hydrogel is engineered for low-temperature wearable electronics, where the combination of ionic conductivity, anti-freezing properties, and mechanical robustness is critical for outdoor and extreme-environment applications. The high tensile strength (6.4 MPa) and low-temperature tolerance (−60 °C) make it a promising candidate for flexible sensors in cold climates. However, the long-term stability of the Na^+^ coordination network under dynamic mechanical loading remains to be fully characterized.

While metal–ligand interactions offer remarkable mechanical tunability and biofunctional versatility, the potential cytotoxicity and long-term biocompatibility of certain metal ions warrant careful consideration in biomedical applications. The biosafety of metal ions is highly dependent on their species, concentration, oxidation state, and coordination environment [55,56,57]. Essential trace elements such as Zn^2+^, Cu^2+^, and Fe^3+^ are generally well-tolerated at physiological concentrations and can even provide therapeutic benefits, including antibacterial activity and angiogenesis promotion [148,151]. However, excessive concentrations of these ions may induce oxidative stress, inflammation, or cellular apoptosis [58,59,60,61,62,63]. For instance, while Cu^2+^ exhibits potent antibacterial properties at concentrations of 10–100 µM, higher doses (>200 µM) have been reported to cause significant cytotoxicity to mammalian cells [148]. Similarly, although Sr^2+^ promotes osteogenesis at optimal concentrations (1–10 mM), excessive Sr^2+^ release may interfere with calcium homeostasis and lead to adverse effects [152]. Non-essential or heavy metal ions such as Ag^+^, Ni^2+^, and high-valence Al^3+^ generally exhibit higher toxicity profiles and require stricter dosage control and thorough biosafety evaluation prior to clinical translation [55,56,57]. Strategies to mitigate metal ion-associated cytotoxicity include precise control over ion release kinetics via tuning the dynamic covalent network degradation rate, encapsulation of metal ions within biocompatible carriers, and the use of chelating agents for on-demand ion sequestration [149]. Comprehensive in vitro cytocompatibility assessments (e.g., CCK-8, live/dead staining, and hemolysis tests) and in vivo histopathological evaluations over extended timeframes remain essential prerequisites for the clinical translation of metal-coordinated hydrogel systems [148,151,152].

In summary, the synergistic combination of metal–ligand interactions with dynamic covalent bonds, through the three functional orientations of mechanical reinforcement, stimuli-responsiveness, and bioactivity, covers a functional spectrum spanning from high-strength and high-toughness structural materials to intelligent responsiveness and further to integrated diagnosis and therapy. Metal–ligand bonds provide tunable bond energies, abundant stimuli-response modes, and metal-specific biological functions, while dynamic covalent bonds endow the network with a long-term framework and self-healing capability. The two form highly efficient synergistic enhancement mechanisms across multiple dimensions, including energy dissipation, orthogonal responsiveness, and controlled-release coupling.

### 3.4. Synergistic Integration of DCBs and Electrostatic Interaction

The core synergistic logic of combining electrostatic interactions with dynamic covalent bonds lies in the following: electrostatic crosslinking provides rapidly reversible, environmentally sensitive sacrificial bonds and intrinsic ionic conductivity, while dynamic covalent bonds furnish a stable elastic framework and chemically responsive dimension, the two achieving functional complementarity in dual-factor regulation and ion conduction.

In contrast to the sequential progression observed in the preceding strategies—where successive systems addressed the limitations of their predecessors—electrostatic interactions with dynamic covalent bonds have evolved along two distinct and parallel tracks, each addressing a different set of application requirements. These two tracks are complementary rather than competitive, and they cannot be arranged in a simple chronological or complexity-based progression.

Track 1: Polyelectrolyte Complex Systems—exploiting the mixing of oppositely charged polymers to achieve instantaneous gelation and shear-thinning behavior. This track focuses on processability and environmental responsiveness, and is particularly suitable for 3D printing, injectable delivery, and gastrointestinal drug delivery applications where rapid shape fixation and salt/pH responsiveness are paramount.

Track 2: Zwitterionic Polymer Systems—through intrachain and interchain electrostatic self-association of positively and negatively charged groups within the same polymer chain, these systems form uniform physical crosslinking networks with strongly bound hydration layers. This track focuses on antifouling properties and ionic conductivity, and is particularly suitable for implantable sensors, electronic skins, and flexible energy storage electrolytes where biocompatibility and signal stability are critical.

The following subsections will present these two tracks in parallel. Rather than one superseding the other, they offer complementary solutions to different sets of challenges, and their respective advantages and limitations will be summarized at the end of this section.

The synergistic interplay between polyelectrolyte complex systems and dynamic covalent bonds is centrally manifested in the temporal division of labor between instantaneous gelation and long-term stability. Oppositely charged polyelectrolytes rapidly associate under appropriate conditions to form an electrostatic physical network, imparting rapid shape fixation and shear-thinning capability to the system; dynamic covalent crosslinking, through subsequent chemical reorganization, provides permanent structural locking and mechanical reinforcement. During processing, electrostatic crosslinking is responsible for instantaneous shape fixation during extrusion, while dynamic covalent crosslinking is responsible for post-printing structural consolidation and long-term stability—this “physical shaping first, chemical locking later” time-sequential strategy constitutes the core of their synergy. In environments with spatial gradients in ionic strength and pH, such as the gastrointestinal tract, electrostatic crosslinking responds to changes in salt concentration and pH to provide the first layer of environmentally sensitive dissociation, while dynamic covalent bonds, responding to specific pH or enzymatic signals, provide a second layer of site-specific triggering. The cascade of these two levels of response enables programmed drug release.

For example, Yang et al. [154] developed an assembled granular hydrogel based on the synergistic construction of hetero-polyelectrolyte electrostatic interactions and boronate ester dynamic covalent bonds. In this system, oppositely charged polymer components (GelMA-DA/SFMA granular hydrogels and HA-PBA/EGCG assembly matrix) rapidly combine to form a polyelectrolyte complex electrostatic network, relying on electrostatic interactions to achieve instantaneous gelation, endowing the material with excellent rapid shape fixation and shear-thinning properties, allowing smooth extrusion through a syringe for molding and processing. The boronate ester dynamic covalent bonds, acting as long-term chemical crosslinking sites, gradually complete bond reorganization after the electrostatic network is formed, permanently locking the overall structure and further enhancing mechanical strength (storage modulus of assembled glycidyl-methacrylate-modified silk fibroin (GSD) significantly higher than GSD + HA-PBA and GelMA/SFMA + HA-PBA groups). To adapt to complex physiological environments, electrostatic interactions can dissociate upon changes in ionic concentration and pH, constituting the first level of environmental response, while boronate ester bonds can specifically respond to signals such as pH and enzymes to achieve secondary triggering. The two levels of response operate in cascade, enabling programmed and controllable release of substances. Rheological tests confirmed that the material exhibits typical shear-thinning behavior (viscosity decreasing with increasing shear rate), excellent self-healing properties (G′ and G″ values returning to original levels after three cycles of alternating strain from 1% to 300%), and good injectability (smooth extrusion through a syringe to form complex shapes such as “flowers”). Tissue adhesion tests demonstrated strong adhesion to porcine tissue, maintaining good fit even after bending and soaking in water. In vitro and in vivo experiments demonstrated that the hydrogel can respond to physiological microenvironmental signals in a cascade manner, achieving temporally controlled release of growth factors (bFGF and PDGF cumulative release of 51.8% and 43.5% over 35 days, respectively), holding promising application prospects in biomedical fields such as abdominal wall defect repair and minimally invasive drug delivery. The assembled granular architecture and cascade-responsive release profile of this hydrogel make it particularly suitable for tissue regeneration in dynamic mechanical environments, such as abdominal wall repair, where the material must withstand cyclic loading while delivering growth factors in a temporally controlled manner. The polyelectrolyte complex network ensures instantaneous gelation upon extrusion, whereas the boronate ester bonds provide secondary pH-responsive drug release.

Qian et al. [155] developed a composite hydrogel with combined conductive, electromagnetic shielding, and sensing functions, constructing a synergistic crosslinking network of hetero-polyelectrolyte electrostatic interactions, metal coordination, and disulfide dynamic covalent bonds. In this poly(PT)/PVA/p-MXene hydrogel based on poly(potassium thioctate), PVA, and positively charged Ti_3_C_2_T_X_ MXene, disulfide dynamic covalent bonds serve as reversible crosslinks, imparting instantaneous self-healing capability (resistance recovery within 2.2 s and stable LED illumination after three cutting-healing cycles), while hydrogen bonds, electrostatic interactions, and Al^3+^ coordination bonds act as physical crosslinks that enhance network cohesion and energy dissipation, achieving exceptional stretchability (gel-0 exceeding 4000% strain, gel-3 maintaining 881.64% strain with 126.70 kPa stress) and robust adhesion to various substrates (wood: 1.57 N/cm, plastic: 1.11 N/cm, metal: 0.99 N/cm). Notably, the introduction of p-MXene endows the hydrogel with high conductivity (1.24 S/m), enabling sensitive strain sensing (GF of 1.05 within 0–200% strain) and outstanding electromagnetic interference shielding effectiveness of 48.56 dB, with stable performance over 1000 stretching cycles. Significantly, the hydrogel exhibits environmentally responsive behavior: the electromagnetic shielding performance can be dynamically tuned through controlled deformation (thinning regions leading to increased electromagnetic wave (EMW) permeation), enabling the innovative application of an EMWs sensing system that monitors Wi-Fi signal strength changes in real-time during finger bending (signal variation from −55 dBm in straight state to −40 dBm in bent state), thereby avoiding material performance degradation caused by electrification-induced thermal effects.

Additionally, the poly(PT) hydrophobic main chains provide robust protection against MXene oxidative degradation, while the hydrogel can regain its EMI shielding performance (recovering to 27.53 dB) by reabsorbing moisture from the air after dehydration, demonstrating recyclability. This multifunctional hydrogel, combining stimuli-responsive EMI shielding tunability, strain sensing, rapid self-healing, and oxidation resistance, holds promising application prospects in flexible electronic skin, wearable strain sensors, human–machine interfaces, and electromagnetic protection devices. This MXene-reinforced hydrogel is uniquely positioned for applications requiring simultaneous strain sensing, electromagnetic shielding, and self-healing in flexible electronics. The high conductivity (1.24 S/m) and EMI shielding effectiveness (48.56 dB) enable real-time motion monitoring and electromagnetic protection. A key limitation, however, is the oxidative degradation susceptibility of MXene, which, although partially mitigated by the hydrophobic poly(PT) main chains, remains a concern for long-term device stability in ambient conditions.

The synergistic interplay between zwitterionic polymer systems and dynamic covalent bonds lies in the integration of antifouling properties and self-healing capability. Zwitterionic groups, through intrachain and interchain electrostatic self-association, form a uniform physical crosslinking network, and their strongly bound hydration layer effectively resists the nonspecific adhesion of proteins and bacteria. Dynamic covalent bonds, in turn, endow this antifouling hydrogel with the ability to recover its structure after damage—microcracks or scratches on the antifouling surface can be repaired through the exchange of dynamic covalent bonds, preventing defect sites from becoming entry points for biofouling. Meanwhile, the intrinsic ionic conductivity of the zwitterionic network, combined with the mechanical reversibility of dynamic covalent bonds, allows the gel electrolyte to maintain continuous ion transport pathways under repeated mechanical deformation, and even after local damage, to restore interfacial contact through covalent exchange. The synergy of the two ensures the dual electrical and mechanical reliability of flexible energy storage devices.

For example, Li et al. [156] developed a zwitterionic hydrogel via a self-aggregation-induced polymerization (SAIP) strategy, integrating the antifouling properties of zwitterionic polymers with the self-healing capability of disulfide dynamic covalent bonds. The SAIP mechanism, driven by the synergistic interaction between hydrophobic 1,2-dithiolane and strongly hydrophilic carboxybetaine moieties, enables efficient spontaneous ring-opening polymerization under mild aqueous conditions without the need for external catalysts, initiators, or harsh stimuli. The material relies on the intrachain and interchain electrostatic self-association of zwitterionic groups to construct a uniform and dense physical crosslinking network, while the incorporation of lipoic acid-modified hyaluronic acid (LAHA) as a crosslinker creates a dynamic hydrogel network with disulfide bonds. The resulting hydrogel exhibits rapid spontaneous gelation (as fast as 116 s for pLACB-LAHA-2.5), excellent injectability and moldability (enabling self-supporting structures such as “123” shapes and flower-like forms), robust mechanical properties (compressive fracture stress up to 3.86 MPa, toughness of 0.32 MJ/m^3^, and compressive modulus of 0.25 MPa), and high water content exceeding 76%. Notably, the hydrogel demonstrates exceptional antioxidant capacity, achieving over 90% DPPH radical scavenging efficiency and significantly reducing intracellular reactive oxygen species levels. The hydrogel maintains stability in water while exhibiting controlled depolymerization in physiological environments (complete dissociation within 4–7 days in PBS) and reductive-responsiveness (rapid degradation upon DTT treatment), ensuring biodegradability.

**Figure 12:**
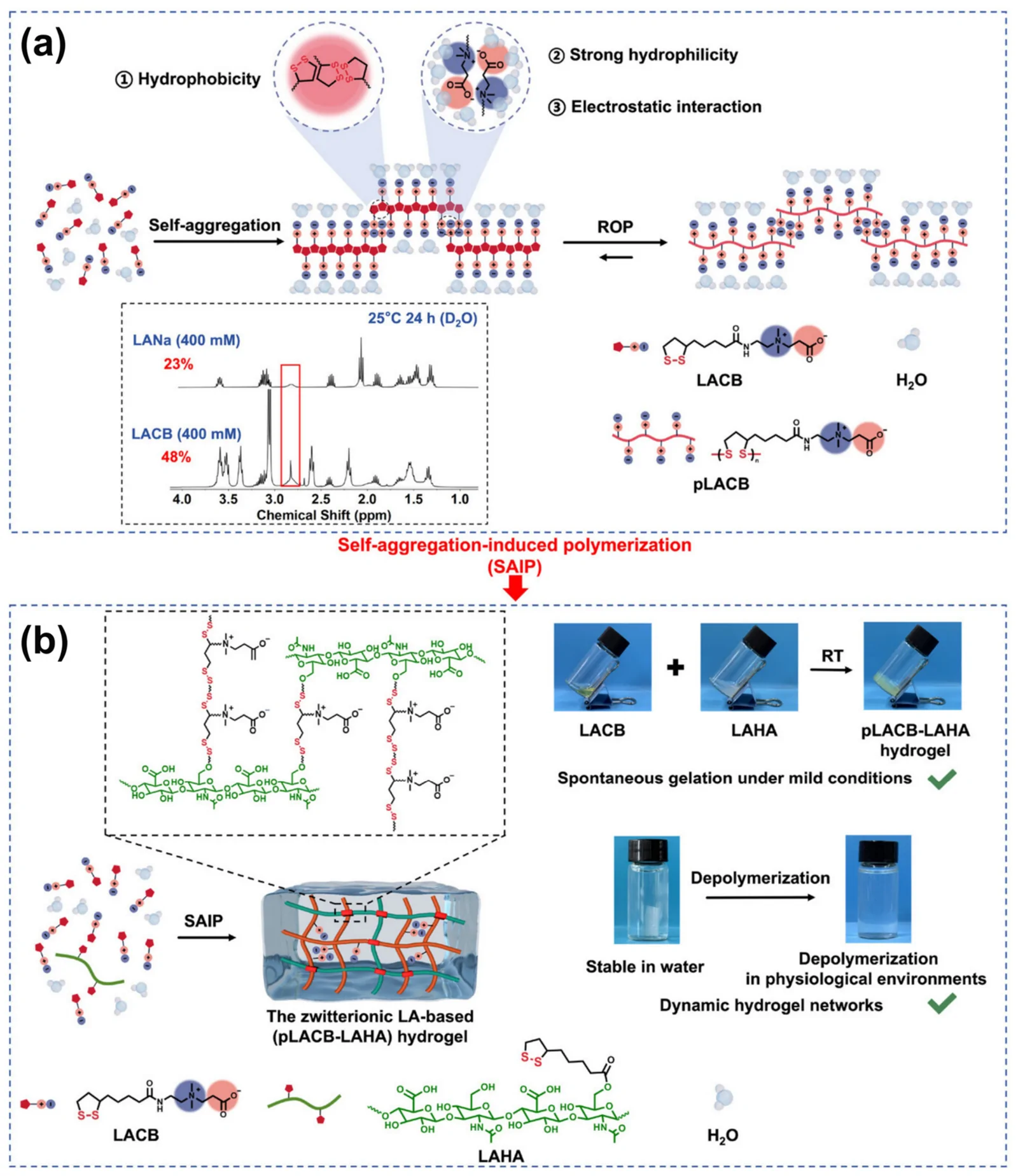
Schematic overview of self-aggregation-induced polymerization (SAIP) and fabrication of pLACB-LAHA zwitterionic dynamic hydrogel, with all visualized molecular and macroscopic elements explicitly correlated to the orthogonal synergistic mechanism of zwitterionic electrostatic physical crosslinks and disulfide dynamic covalent bonds. (**a**) Molecular schematic illustrating SAIP behavior of LACB monomers: the diagram visually displays the coexistence of hydrophobic 1,2-dithiolane rings and highly hydrophilic carboxybetaine zwitterionic segments, whose synergistic amphiphilic interaction drives monomer self-concentration and spontaneous ring-opening polymerization without external triggers. In the accompanying comparative ^1^H NMR spectrum, the red rectangular frame highlights the diagnostic proton signal region used to quantify ring-opening polymerization conversion. The comparative ^1^H NMR data presented in this panel further reflect the higher polymerization efficiency originating from zwitterion-mediated aggregate formation, which serves as the prerequisite for constructing zwitterionic physical crosslink networks. (**b**) Assembly schematic for pLACB-LAHA hydrogel and corresponding macroscopic photos: in the network sketch, orange lines represent polymerized pLACB chains, green lines denote LAHA polysaccharide segments, red blocks indicate cross-linking junctions, and blue-pink spheres visualize zwitterionic carboxybetaine moieties. The graphic distinguishes two orthogonal crosslinking modes coexisting within one single network—intermolecular electrostatic stacking of zwitterionic carboxybetaine groups forms dense antifouling physical crosslinks, while disulfide dynamic covalent bonds from LAHA crosslinkers provide reversible structural repair capacity. The photographs of rapid room-temperature gelation and PBS-triggered depolymerization directly visualize the complementary functional coordination of the two dynamic interactions: zwitterionic hydration networks confer stable antifouling matrix frameworks, and exchangeable disulfide bonds endow the system with reconfigurable, degradable dynamic characteristics, fully demonstrating the orthogonal synergy between electrostatic supramolecular assembly and dynamic covalent chemistry. Reprinted with permission from Ref. [156].

Furthermore, the hydrogel exhibits excellent biocompatibility, with low protein adsorption (BSA adsorption of only 1.1 ± 0.1 μg/cm^2^, comparable to highly biocompatible pCBMA), high cell viability (comparable to control groups in MTT assays), and only mild inflammatory responses upon subcutaneous implantation in mice (complete degradation within 7 days, unlike non-degradable pHEMA and pCBMA controls which remained for 14 days with visible inflammation). By virtue of its multiple advantages including mild gelation, antioxidant properties, and cytoprotective capabilities, the hydrogel can efficiently encapsulate and provide long-term protection for probiotics and mammalian cells under various challenging conditions, including simulated gastric fluid (preserving 91.3% probiotic viability), oxidative stress environments (maintaining 63.7% viability vs. 11.9% in control), and long-term refrigerated storage (60.3% viability vs. 5.0% in control after 14 days), holding broad application prospects in biomedical fields such as living cell encapsulation, minimally invasive delivery, and tissue repair (Figure 12). The zwitterionic design, combined with disulfide dynamic crosslinks, makes this hydrogel ideal for cell encapsulation and probiotic delivery applications. The strong hydration layer provides antifouling properties that resist protein adsorption and immune recognition, while the reductive responsiveness enables on-demand degradation in physiological environments. The rapid gelation (116 s) and cytoprotective capability (91.3% probiotic viability in simulated gastric fluid) are key advantages for oral delivery of live therapeutics.

In summary, the polyelectrolyte complex and zwitterionic polymer systems represent two parallel, complementary strategies rather than a sequential progression. Polyelectrolyte complexes excel in injectable and 3D-printable applications where rapid shape fixation and shear-thinning behavior are essential, and their responsiveness to salt and pH provides the first layer of environmental sensitivity in gastrointestinal delivery and similar applications. Zwitterionic systems, by contrast, are uniquely suited for antifouling interfaces and ionic conductive devices, where their strongly bound hydration layers and intrinsic ionic conductivity are critical. The choice between the two tracks depends on the target application: polyelectrolyte complexes for scenarios requiring processability and environmental responsiveness, and zwitterionic systems for those requiring biocompatibility and signal stability.

Looking ahead, several key directions merit particular attention for advancing synergistic electrostatic interaction/DCBs. First, integrating polyelectrolyte complex and zwitterionic strategies into a unified system remains largely unexplored. A hybrid architecture—such as zwitterionic surface layers grafted onto polyelectrolyte complex bulk networks—could potentially combine rapid shape fixation, environmental responsiveness, antifouling properties, and ionic conductivity within a single material. Second, the dynamic nature of electrostatic crosslinking offers a unique opportunity for 4D printing, where printed structures could undergo programmable shape evolution in response to changes in ionic strength or pH after fabrication. Third, the salt- and pH-responsiveness of these systems has been primarily studied in simple buffer solutions; systematic investigation in complex physiological environments—including serum, inflammatory exudates, and gastrointestinal fluids—is urgently needed to assess their translational potential for drug delivery and implantable devices. Fourth, machine learning-assisted formulation optimization could dramatically accelerate the identification of optimal polymer charge ratios and molecular weights for specific applications, thereby reducing reliance on trial-and-error approaches that currently dominate this field. Progress along these directions will be essential to transform electrostatic interaction/DCB synergistic hydrogels from promising laboratory concepts into robust, application-ready material platforms.

### 3.5. Synergistic Integration of DCBs and Hydrophobic Interaction

The core synergistic logic of combining hydrophobic physical crosslinking with dynamic covalent bonds lies in the following: hydrophobic crosslinking provides reversible physical network junctions and the capacity for loading hydrophobic cargos, while dynamic covalent bonds furnish a responsive framework capable of reversible cleavage under chemical or environmental stimuli, the two forming complementary integration across multiple structural hierarchies. In hydrogel fabrication, based on the structural organization of hydrophobic domains, three distinct levels can be distinguished: single-molecule hydrophobic modification, where long alkyl chains or cholesteryl groups grafted onto hydrophilic backbones form dispersed hydrophobic microdomains as physical crosslinks, while Schiff base or boronate ester bonds provide self-healing, pH responsiveness, and underwater adhesion—this level is suited for shape-memory and underwater adhesive hydrogels; block copolymer micelle crosslinking, where micellar cores serve as hydrophobic drug reservoirs and physical crosslinks with continuously tunable density, while dynamic covalent bonds provide stepwise mechanical modulation via chemical stimuli, enabling multi-mode tunability and spatiotemporal controlled release—this level is ideal for injectable drug depots; and nano-hydrophobic domains, where polymersomes or hydrophobically modified nanoparticles simultaneously serve as crosslinking nodes, drug reservoirs, and imaging carriers, while dynamic covalent bonds function as a disintegration switch triggered by pathological stimuli for synchronized structural breakdown and cargo release—this level enables integrated crosslinking, drug loading, and controlled release hydrogels.

In the single-molecule hydrophobic modification strategy, long alkyl chains (e.g., octyl groups from octenyl succinic anhydride) are grafted onto a hydrophilic polysaccharide backbone (such as chitosan or alginate). These grafts spontaneously self-assemble in aqueous media to form dispersed hydrophobic microdomains, which serve as reversible physical crosslinking points that effectively enhance the mechanical stability of the hydrogel network. Concurrently, dynamic covalent bonds—typically Schiff base linkages formed between aldehyde groups on oxidized polysaccharides (e.g., sodium alginate) and amino groups on the modified backbone—are introduced into the system. This dual crosslinking architecture, combining physical hydrophobic associations with chemical dynamic covalent bonds, endows the hydrogel with rapid autonomous self-healing capability, as disrupted Schiff base linkages can readily re-form at fractured interfaces. The reversible nature of the Schiff base bonds also renders the hydrogel pH-responsive, enabling tunable degradation or controlled cargo release. Furthermore, the hydrophobic microdomains serve as reservoirs for hydrophobic drugs, achieving high encapsulation efficiency and sustained release. By further functionalizing the backbone with bioactive moieties—such as catechol groups for enhanced adhesion and antioxidant activity, or quaternary ammonium salts for intrinsic antibacterial properties—the system can be tailored for specific biomedical applications, particularly infected wound healing.

For example, Li et al. [157] developed a single-molecule hydrophobically modified self-healing hydrogel based on dodecyl quaternary ammonium salt (Q12)-modified carboxymethyl chitosan (Q12-CMC) and oxidized sodium alginate (ASA) via dynamic Schiff base and Fe^3+^ coordination bonds to achieve antibacterial wound healing. This system adopts a single-molecule hydrophobic modification strategy by grafting dodecyl (C12) long-chain alkyl groups onto the hydrophilic carboxymethyl chitosan backbone via quaternary ammonium linkage, forming dispersed hydrophobic microdomains through hydrophobic association that serve as reversible physical crosslinking points, while the positively charged quaternary ammonium groups provide intrinsic antibacterial activity against both Gram-negative and Gram-positive bacteria. The dynamic Schiff base bonds between aldehyde groups of ASA and amino groups of Q12-CMC, together with Fe^3+^ coordination bonds, enable rapid autonomous self-healing: the separated hydrogels autonomously merged within 30 s at body temperature without any external stimulus, with G′ and G″ recovering to initial values upon strain return from 200% to 1%, and the collapse-recovery cycles could be alternated repeatedly, confirming excellent self-healing capacities. The dodecyl long-chain alkyl quaternary ammonium salt imparts superior antibacterial properties: the hydrogel effectively inhibited more than 98% of both *S. aureus* and *E. coli*, compared with only 50% reduction in hydrogels without Q12 modification. The antibacterial mechanism involves the synergistic effect of quaternary amine groups with 12-carbon alkyl chains and hydrophobic insertion, resulting in bacterial lysis.

**Figure 13:**
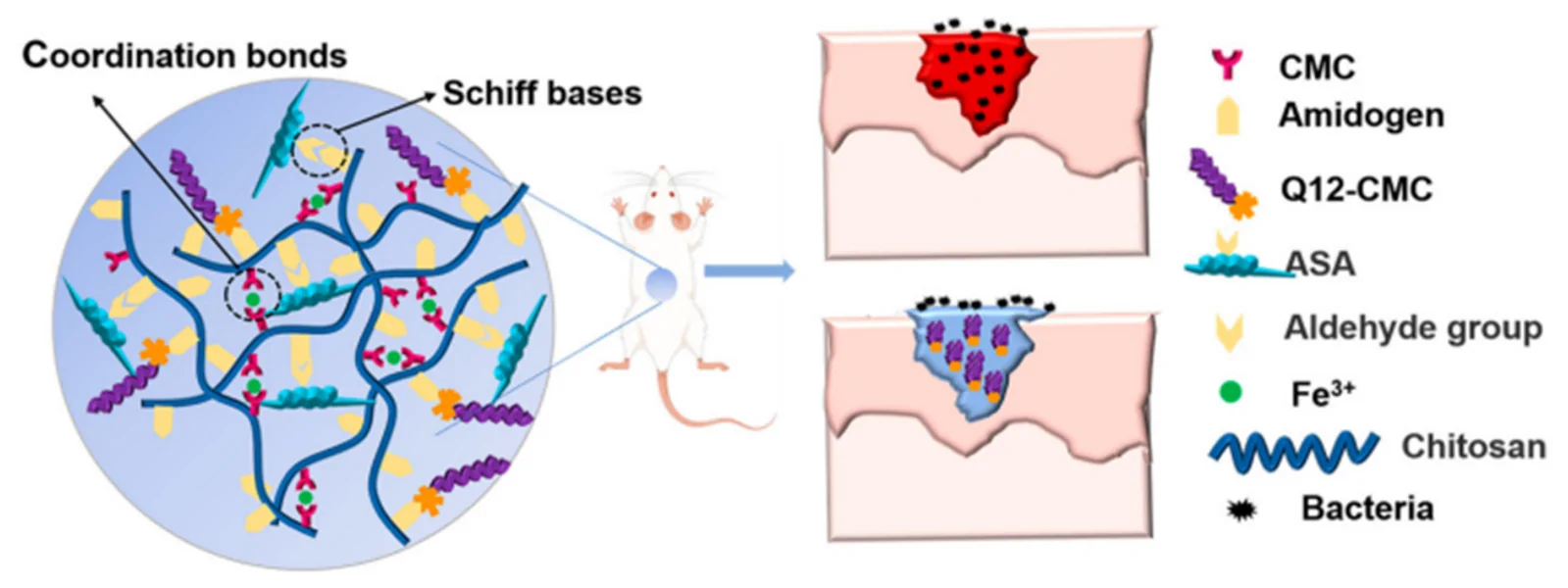
Schematic diagram illustrating the fabrication, internal crosslink architecture and in vivo wound treatment application of QAF hydrogel, with all visualized structural units explicitly mapped to the synergistic dual-crosslinking mechanism of hydrophobic physical aggregates and dynamic Schiff/Fe^3+^ coordination bonds. The left network schematic visually presents two orthogonally functioning reversible interactions coexisting within one unified polysaccharide framework: dodecyl quaternary ammonium alkyl side chains grafted on Q12-CMC spontaneously aggregate into hydrophobic microdomains to form reversible physical crosslinks, while dynamic Schiff base bonds between ASA aldehyde groups and Q12-CMC amines, together with Fe^3+^ metal-coordination interactions, constitute dual chemical crosslinking skeletons. The diagram directly demonstrates their kinetically complementary synergy: hydrophobic associations provide instantaneous interfacial adhesion upon fracture, and pH-responsive Schiff/coordination bonds complete long-term network reconstruction, jointly enabling rapid spontaneous self-healing. The right wound application graphic further reflects the functional orthogonality of the two crosslink types, where quaternary ammonium hydrophobic domains deliver intrinsic antibacterial performance, and the dynamic dual chemical network maintains intact, adaptive hydrogel scaffolds to accelerate infected skin tissue regeneration. Reprinted with permission from Ref. [157].

Meanwhile, the dynamic Schiff base and coordination crosslinking architecture renders the hydrogel pH-responsive and hemocompatible, with a hemolysis ratio of only 0.26%, superior hemostatic properties (BCI value of 0.44%), and significantly reduced blood loss (21.67 ± 3.76 mg vs. 133.40 ± 8.06 mg in the control group). In a mouse full-thickness skin defect model, the QAF hydrogel group exhibited a 66.45% wound-healing rate on day 7, outperforming the control groups (39.95%), and achieved complete wound closure by day 14, with reduced inflammatory response, increased collagen deposition, and improved vascularization. This hydrogel, featuring natural marine-derived polysaccharides (chitosan and alginate) and simple preparation, combines excellent self-healing capability, potent antibacterial activity, good hemocompatibility and cytocompatibility, and effective wound healing promotion, holding broad application prospects in the fields of infected wound dressings, burn wound repair, and postoperative tissue regeneration (Figure 13). This single-molecule hydrophobic modification hydrogel is specifically tailored for infected wound healing, where the dual antibacterial mechanism (quaternary ammonium + hydrophobic insertion) and rapid self-healing (30 s at body temperature) are critical for conformable wound coverage and bacterial eradication. The marine-derived polysaccharide backbone ensures biocompatibility, though the relatively low mechanical strength compared to synthetic polymer systems may limit its use in mechanically demanding wound sites.

**Figure 14:**
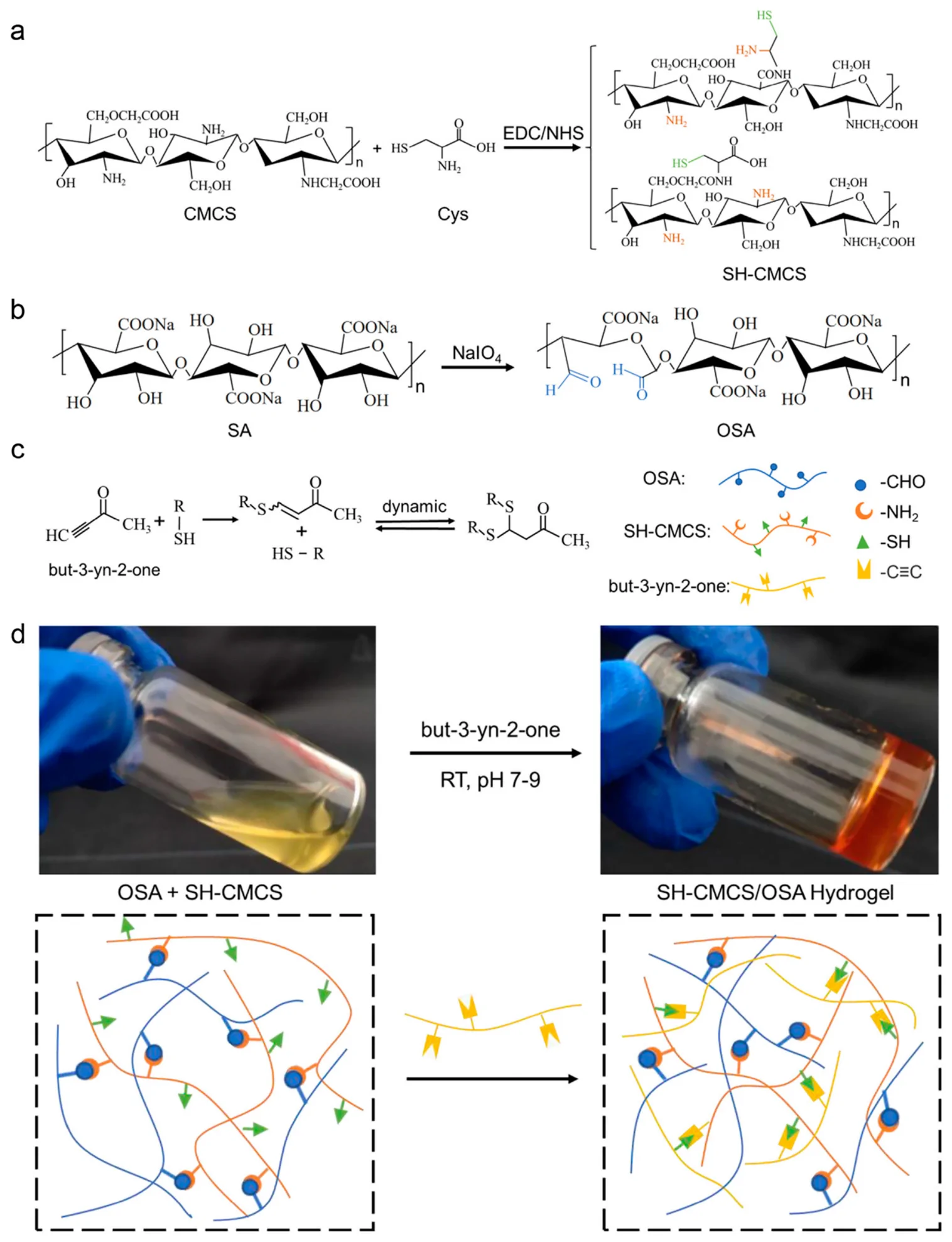
Synthetic schematic of SH-CMCS/OSA dual-dynamically crosslinked polysaccharide hydrogel, with each synthetic and crosslinking panel visually corresponding to the synergistic mechanism of hydrophobic physical aggregation and two types of dynamic covalent bonds. (**a**) Synthetic route of oxidized sodium alginate (OSA), which introduces aldehyde functional groups required for forming pH-sensitive Schiff base dynamic covalent crosslinks. (**b**) Synthetic pathway of cysteine-grafted carboxymethyl chitosan (SH-CMCS); the grafted thiol moieties shown in the diagram provide reactive sites for thiol-alkynone crosslinking, while the chitosan backbone’s hydrophobic segments visualized here spontaneously aggregate to form physical crosslink microdomains as described in the synergistic framework. (**c**) Schematic of thiol-alkynone double addition reaction, visually demonstrating its kinetically differentiated dual reactivity: the initial thiol-alkyne addition is irreversible, and the secondary crosslinking reaction is reversible, constituting the second dynamic covalent network that coordinates with Schiff bonds to realize multi-stage self-repair. (**d**) Integrated hydrogel formation schematic, explicitly showing the orthogonal synergistic triple crosslink architecture assembled from three interactive units: hydrophobic physical aggregates from chitosan segments, Schiff base dynamic imine bonds between OSA aldehydes and SH-CMCS amines, and reversible thiol-alkynone covalent linkages, fully mapping the dual dynamic covalent plus hydrophobic physical crosslinking synergistic system. Reprinted with permission from Ref. [158].

Zhang et al. [158] developed a dynamic double cross-linked self-healing polysaccharide hydrogel based on cysteine-modified carboxymethyl chitosan (SH-CMCS) and oxidized sodium alginate (OSA) via Schiff base and thiol-alkynone reactions to achieve enhanced mechanical properties and antibacterial wound healing. This system introduces thiol groups onto the carboxymethyl chitosan backbone through cysteine grafting, with the inherent hydrophobic acetylglucosamine residues of chitosan providing local hydrophobic microdomains that serve as physical crosslinking points through hydrophobic association, while the thiol-alkynone double addition reaction creates a second dynamic covalent network, substantially improving mechanical stability. The Schiff base bonds between aldehyde groups of OSA and amino groups of SH-CMCS provide rapid self-healing capability, while the reversible thiol-alkynone double addition reaction between sulfhydryl groups and the carbon–carbon triple bond of but-3-yn-2-one (methyl ethynyl ketone) imparts additional dynamic crosslinking: the first addition between sulfhydryl and alkyne is irreversible, whereas the second addition between the single adduct and the second sulfhydryl group is reversible, enhancing the dynamic self-healing capability of the hydrogel. The dual dynamic covalent network enables outstanding mechanical performance with gelation time as short as 230 s at optimal but-3-yn-2-one concentration (5 μL/mL), storage modulus significantly higher than loss modulus, and excellent shear-thinning behavior, with the hydrogel withstanding large elastic deformation up to 100% strain before network disruption, and G′/G″ recovery upon strain return from 550% to 1% confirming excellent self-healing and mechanical strength. The hydrogel exhibits good antibacterial properties against both *E. coli* and *S. aureus* through synergistic action of positively charged amino groups on chitosan and Schiff base complexes, with significantly elevated bacterial reactive oxygen species (ROS) levels confirming the antibacterial mechanism. In vivo compatibility tests demonstrated that the hydrogel effectively inhibited inflammation, promoted skin tissue regeneration, and accelerated wound healing, resulting in a denser skin structure, fewer inflammatory cells, and regenerated hair follicles and blood vessels compared with control groups. This hydrogel, featuring a dual dynamic crosslinking architecture (Schiff base and thiol-alkynone) and simple preparation, combines enhanced mechanical properties, rapid gelation, excellent self-healing capability, good antibacterial activity, and in vivo biocompatibility, holding broad application prospects in the fields of skin tissue engineering, infected wound dressings, and postoperative tissue regeneration (Figure 14).

Despite these advantages, the single-molecule hydrophobic modification strategy faces inherent limitations: the loading capacity of dispersed microdomains for hydrophobic guests remains constrained by their small and loosely packed nature, and the crosslinking density is not easily tunable because each grafted chain acts as an independent association unit with limited coordination. To overcome these limitations, block copolymer micelle crosslinking was developed, in which amphiphilic block copolymers self-assemble into core–shell micellar structures with well-defined hydrophobic cores. These micelles serve as robust physical crosslinking nodes with significantly higher association numbers and cohesive energy, while providing a spacious reservoir for hydrophobic drugs or functional molecules. Moreover, the micellar crosslinking density is continuously tunable with temperature or concentration, offering a level of mechanical modulation unattainable by dispersed single-molecule domains. This evolution from single-molecule to micellar hydrophobic crosslinking substantially increases structural hierarchy and functional capacity.

Within this micellar framework, the synergy with dynamic covalent bonds manifests in two complementary aspects. First, the micellar crosslinking density varies continuously with environmental conditions (temperature or concentration), whereas the dynamic covalent crosslinking density is regulated in a stepwise manner by chemical stimuli (pH or redox); the combination of continuous and stepwise modulation endows the hydrogel with multi-mode mechanical tunability over a broad range. Second, in drug delivery applications, the micelle cores serve as solubilization and storage compartments for hydrophobic drugs, while the density of dynamic covalent crosslinks governs the overall network degradation and thus the drug release kinetics. The micelles are responsible for drug encapsulation and protection, and the covalent network acts as the controlled-release switch—the two functions are clearly defined and synergistically linked, enabling precise spatiotemporal control over therapeutic delivery.

**Figure 15:**
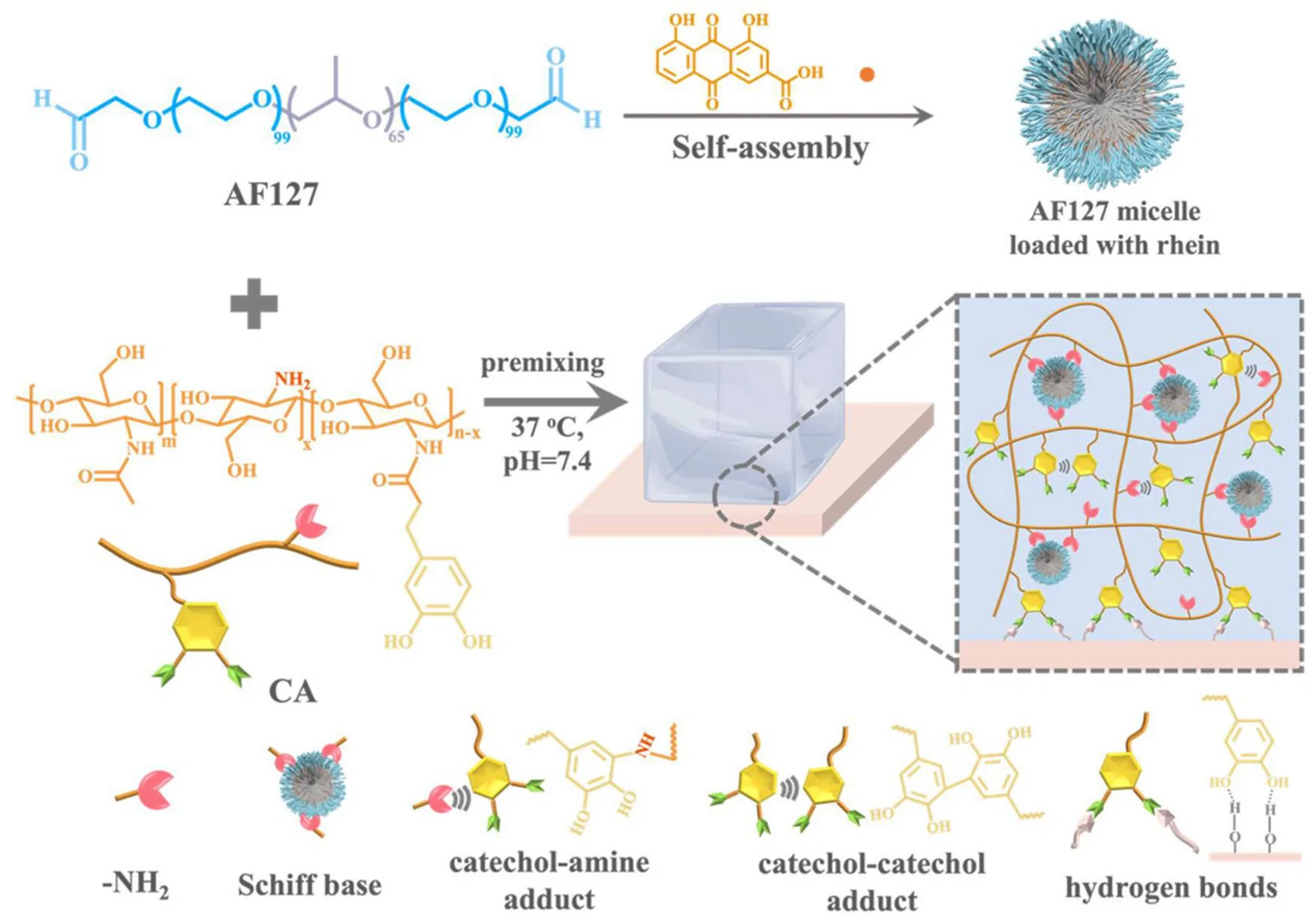
Synthetic schematic of micelle-crosslinked CA/AF dual synergistic hydrogel, with all visualized structural units explicitly mapped to the complementary coordination mechanism of micellar physical crosslinks and Schiff base dynamic covalent bonds described in the text. The graphic separately illustrates two core building blocks: catechol-functionalized chitosan (CA) chains and aldehyde-terminated Pluronic F127 (AF127) amphiphiles that self-assemble into micellar aggregates. The diagram visually distinguishes the two orthogonal crosslinking motifs: AF127 micelles act as continuous physical crosslink nodes and hydrophobic drug storage compartments, while pH-sensitive Schiff base imine bonds formed between CA amino groups and micelle aldehyde groups constitute discrete stimulus-responsive dynamic covalent skeletons. This schematic directly presents their functionally separated synergistic logic: micellar assemblies undertake hydrophobic drug encapsulation and temperature/concentration-dependent continuous network modulation, whereas Schiff base bonds serve as pH-triggered regulatory switches to control overall matrix degradation and payload release kinetics, fully visualizing the multi-mode tunable and spatiotemporally controllable dual crosslinking architecture. Reprinted with permission from Ref. [159].

For instance, Wang et al. [159] developed a multifunctional hydrogel dressing constructed with aldehyde-modified Pluronic F127 (AF127) micelles as crosslinking units combined with Schiff base dynamic covalent bonds. This system relies on the block copolymer micelle crosslinking mode, coupling hydrophobic interactions and dynamic covalent bonds to achieve multiple functional synergies. The amphiphilic Pluronic F127 self-assembles into micellar structures in aqueous solution, with the hydrophobic core capable of efficiently encapsulating hydrophobic rhein. The micelles serve as core physical crosslinking nodes to build the gel framework, while Schiff base dynamic covalent bonds form between aldehyde-modified micelles and catechol-functionalized chitosan, constructing a complete dynamic crosslinking network with a gelation time that can be flexibly adjusted from 65 to 20 min by varying the micelle concentration. Rheological tests demonstrated that the storage modulus (G′) of all hydrogels significantly exceeded the loss modulus (G″), confirming stable gel structures, with mechanical strength increasing notably as AF127 content rose from 7.2% to 9.0% (G′ reaching the highest level). Alternating high–low-strain cyclic experiments confirmed rapid network recovery after damage, exhibiting excellent self-healing performance, while the viscosity decreased with increasing shear rate, demonstrating typical shear-thinning and injectable properties. The catechol groups in the chitosan backbone form strong hydrogen-bonding interactions with hydroxyl and amino groups on tissue surfaces, enabling firm adhesion to various tissues, including skin and organs, and withstanding a 10 g load without detachment. The Schiff base bonds impart pH-responsive characteristics: under acidic conditions (pH 6.0), cumulative rhein release reached 41.34% within 48 h, significantly higher than at pH 6.8 (28.04%) and pH 7.4 (19.35%), demonstrating precise pH-responsive drug release capability. In vivo results from an *S. aureus*-infected full-thickness skin defect model showed that after 14 days of treatment, the drug-loaded hydrogel group achieved a wound healing rate of 97.66%, with completely regenerated epidermis without obvious scar formation. This micelle-crosslinked dynamic hydrogel integrates multiple functions including drug loading, self-healing, tissue adhesion, and pH-responsive controlled release, and holds exceptionally high application value in the fields of medical dressings for infected skin wound repair and chronic wound care (Figure 15). The micellar crosslinking architecture of this hydrogel enables dual functionality: the hydrophobic cores serve as drug depots for sustained release of rhein, while the Schiff base network provides pH-responsive dissociation in the acidic wound microenvironment. This hierarchical design is to chronic wound management, where prolonged antibacterial and anti-inflammatory activity is required. The pH-dependent release profile (41.34% at pH 6.0 vs. 19.35% at pH 7.4) ensures higher drug availability in infected wounds, though the potential for burst release upon initial gelation should be carefully controlled.

Despite the successful integration of drug loading and mechanical reinforcement achieved by block copolymer micelle crosslinking, the micelles themselves serve primarily as passive reservoirs—their disintegration and cargo release are typically coupled to the degradation of the overall network rather than being independently triggered. Nano-hydrophobic domains, such as polymersomes or hydrophobically modified nanoparticles, represent a further advancement in structural sophistication. These nanoscale assemblies not only function as physical crosslinking nodes and drug reservoirs but also incorporate responsive features that enable triggered release. When dynamic covalent bonds in the surrounding network undergo selective cleavage in response to pathological stimuli, the resulting network disintegration causes the nano-hydrophobic domains to release their cargo. This establishes a direct link between network responsiveness and cargo release, achieving synchronous triggering of reduced crosslinking density and drug release—a coupling that the micelle systems cannot accomplish. Furthermore, the use of nanoparticles as hydrophobic domains introduces additional functionalities such as imaging contrast, photothermal conversion, or magnetic responsiveness, opening up possibilities for theranostic applications.

Nano-hydrophobic domains employ polymersomes or hydrophobically modified nanoparticles as crosslinking nodes, and their synergistic interplay with dynamic covalent bonds lies in the integration of “crosslinking, drug loading, and responsiveness” into a single functional entity. The nano-hydrophobic domains simultaneously assume the triple roles of physical crosslinking, drug reservoirs, and imaging probe carriers, while dynamic covalent bonds serve as the responsive disintegration switch. Under pathological stimuli such as reducing or acidic conditions, the selective cleavage of dynamic covalent bonds leads to network disintegration, and the nano-hydrophobic domains subsequently release their encapsulated drug, achieving synchronous triggering of reduced crosslinking density and drug release. This precise coupling of structural disintegration and functional release constitutes the core design principle of such systems.

For example, Wang et al. [160] developed a nanogel delivery system based on acylated rapeseed protein isolate. This material relies on nano-hydrophobic domain structures, combining hydrophobic interactions with disulfide dynamic covalent bonds to achieve the integration of crosslinking, drug loading, and structural stability. After thermal denaturation of the acylated rapeseed protein, hydrophobic groups aggregate to form a nano-hydrophobic core, while intermolecular disulfide dynamic covalent networks are simultaneously generated. The two synergistically form a stable core–shell nanogel. The nanogel exhibited a uniform particle size, with a hydrodynamic diameter of 170 nm and a PDI of 0.26. It maintained good dispersion over a wide pH range (2.0–9.0) and in 0–200 mM salt environments. The structure and particle size showed no significant changes even after lyophilization-reconstitution and ten-fold dilution, demonstrating excellent acid–base tolerance, ionic stability, and processing adaptability. Performance tests revealed that the system achieved an encapsulation efficiency of up to 95.1% for hydrophobic curcumin. After drug loading, the nanogel had a particle size of 176.4 nm and a ζ-potential of −26.3 mV, still exhibiting outstanding dispersibility. This protein-based nanogel, through the synergistic enhancement of hydrophobic interactions and dynamic disulfide bonds, combines high drug loading capacity, excellent stability, and improved bioactivity, holding promising application prospects in the fields of functional foods, nutraceutical delivery, and antitumor drug carriers.

In summary, the synergistic combination of hydrophobic interactions with dynamic covalent bonds, through a structural hierarchy progressing from single-molecule side groups to block copolymer micelles and, ultimately, to nano-hydrophobic domains, achieves the functional integration of temperature responsiveness, underwater adhesion, and controlled release of hydrophobic drugs. The characteristic temperature-strengthening effect of hydrophobic crosslinking and the thermal reversibility or chemical responsiveness of dynamic covalent bonds form an orthogonal complementarity, while the drug-solubilizing capacity of hydrophobic domains and the controllability of the covalent network synergistically couple loading and release, providing a unique design dimension for intelligent drug delivery and shape-memory materials.

Despite these advances, several critical challenges and opportunities merit future investigation. First, the molecular-level understanding of hydrophobic domain formation and its dynamic exchange with the surrounding aqueous environment remains limited. Advanced characterization techniques—such as cryo-electron microscopy, solid-state NMR, and coarse-grained molecular dynamics simulations—are urgently needed to elucidate the structure–dynamics–property relationships of hydrophobic microdomains, thereby enabling more rational design of temperature-responsive and underwater adhesive systems. Second, translating hydrophobic interaction/DCBs-based hydrogels from aqueous environments to physiological conditions poses a major challenge. Most existing studies have been conducted in simple buffer systems, yet the presence of proteins, lipids, and cells in biological fluids can significantly alter hydrophobic association and swelling behavior. Systematic evaluation under physiologically relevant conditions—including serum-containing media, flowing fluids, and dynamic mechanical loading—is essential to assess their true translational potential for drug delivery and tissue adhesion. Third, integrating multiple hydrophobic structural motifs (e.g., combining long alkyl chains with aromatic groups or cholesteryl moieties) within a single network could create hierarchical hydrophobic domains with distinct temperature sensitivities and binding affinities, enabling multi-stage drug release or sequential shape-memory effects. Fourth, scalable and reproducible fabrication methods remain underdeveloped. Current approaches rely heavily on labor-intensive synthesis and purification steps; the development of continuous processing techniques—such as microfluidic-assisted assembly or extrusion-based manufacturing—will be critical for translating these materials from laboratory-scale demonstrations to industrial applications. Addressing these challenges will be essential to unlock the full potential of hydrophobic interaction/DCB synergistic hydrogels for intelligent drug delivery, underwater adhesives, and shape-memory devices.

### 3.6. Synergistic Integration of DCBs and π–π Stacking Interaction

The core synergistic logic of combining π–π stacking with dynamic covalent bonds lies in the following: π–π stacking provides rapidly reversible physical crosslinking points and molecular recognition capability, while dynamic covalent bonds furnish the mechanical framework and chemically responsive dimension, the two complementing each other in self-healing cascades, stimuli-responsive orthogonality, and network structure regulation.

Unlike the sequential progression observed in hydrogen bond- and metal-coordination-based systems, the development of π–π stacking and dynamic covalent bond systems has diversified into three distinct functional orientations, each addressing a different design priority rather than overcoming a predecessor’s limitation. These three orientations—self-healing, stimuli-responsiveness, and structure regulation—represent parallel evolutionary branches rather than a single linear progression.

Orientation 1: Self-Healing-Oriented Systems—In these systems, π–π stacking provides the rapid initial anchoring of fractured surfaces through its fast exchange kinetics, while dynamic covalent bonds subsequently complete the permanent repair through slow covalent exchange. The primary design goal is to achieve a balance between healing speed and healing efficiency, with the aromatic content and dynamic covalent chemistry independently tunable to customize the repair performance.

Orientation 2: Stimuli-Responsive-Oriented Systems—In these systems, the responsiveness of π–π stacking to temperature, light, and solvent environment is exploited as a functional output. Systems containing azobenzene groups are particularly representative, where UV/visible light reversibly modulates the stacking state and thus the physical crosslinking density. Dynamic covalent bonds provide an independent chemically responsive dimension, enabling the gel to achieve stepwise mechanical regulation under multiple stimuli. The key design goal is orthogonality: light and chemical signals act on π–π stacking and dynamic covalent bonds, respectively, without mutual interference.

Orientation 3: Structure-Regulation-Oriented Systems—In these systems, the directionality of π–π stacking is exploited to guide the formation of ordered network microstructures, such as nanofibers, lamellar structures, or helical assemblies. Dynamic covalent bonds lock these structures in place or endow them with reversible reorganization capability, allowing the gel microstructure to reversibly switch between thermodynamically equilibrated and kinetically trapped states. The key design goal is multi-scale structural control, from molecular stacking to nanostructures to macroscopic gels.

The following subsections will present these three orientations in parallel. They are not intended to supersede one another; rather, they offer distinct pathways for different application requirements, and their respective advantages will be summarized at the end of this section.

In self-healing-oriented systems, the synergistic core of π–π stacking and dynamic covalent bonds lies in the cascade coordination of repair kinetics. π–π stacking exhibits a fast exchange rate, enabling rapid re-establishment of inter-aromatic stacking interactions at fractured surfaces after damage and quickly aligning and apposing the two fractured interfaces at the molecular level. Dynamic covalent bonds, with their relatively slow exchange rate, form permanent connections at the interface through covalent bond cleavage and reformation after π–π stacking has accomplished the initial anchoring. This “fast stacking anchorage-slow covalent locking” repair sequence enables the hydrogel to recover high mechanical strength within a relatively short time, with repair efficiency surpassing that of systems relying solely on a single dynamic bond. The type and grafting density of the aromatic groups determine the binding strength and reformation rate of π–π stacking, while the type of dynamic covalent bond dictates the completion time of permanent repair and the final mechanical recovery ratio. The two are independently tunable, offering a flexible design space for the on-demand customization of the self-healing rate and repair strength of hydrogels.

For example, Marić et al. [161] developed a biocompatible supramolecular hydrogel based on aromatic pseudopeptide building blocks, integrating π–π stacking with disulfide and acylhydrazone dual dynamic covalent bonds. This system relies on aromatic ring-stacking interactions and two types of orthogonal dynamic covalent bonds to form a kinetically cascaded, synergistic network, achieving efficient self-healing and customizable performance regulation through a “fast stacking anchorage—slow covalent locking” repair mechanism. The rigid aromatic core within the molecular structure can form π–π stacking with a relatively fast exchange rate. After the gel network is fractured, the aromatic rings can rapidly re-stack at the fractured surfaces, precisely apposing and temporarily anchoring the two sides at the molecular level. On this basis, the disulfide and acylhydrazone dynamic covalent bonds, possessing relatively slower exchange rates, progressively undergo bond reorganization, forming stable permanent connections at the apposed interface. The temporal coordination of the two greatly enhances the self-healing efficiency, with performance superior to that of gels containing only a single type of dynamic bond. The structure and grafting density of the aromatic groups can regulate the binding strength and reformation rate of π–π stacking, while the two types of dynamic covalent bonds—disulfide and acylhydrazone—can independently optimize bonding kinetics and final mechanical properties, enabling flexible customization of self-healing rate and repair strength. The hydrogel can undergo gelation at building block concentrations as low as 0.025 wt%, with gelation time flexibly adjustable within the range of 2–10 min depending on the type of crosslinker. Rheological tests quantitatively validated the self-healing performance: after subjecting the gel to large strain to disrupt the network, the strain was removed and the modulus recovery was monitored. The storage modulus of the gel gradually recovered; although the fiber reassembly rate was slower than the initial gelation rate, the network structure was effectively re-established. Gels prepared with different crosslinkers all achieved considerable modulus recovery after structural damage, directly confirming the self-healing capability endowed by the synergy of π–π stacking and dual dynamic covalent bonds.

**Figure 16:**
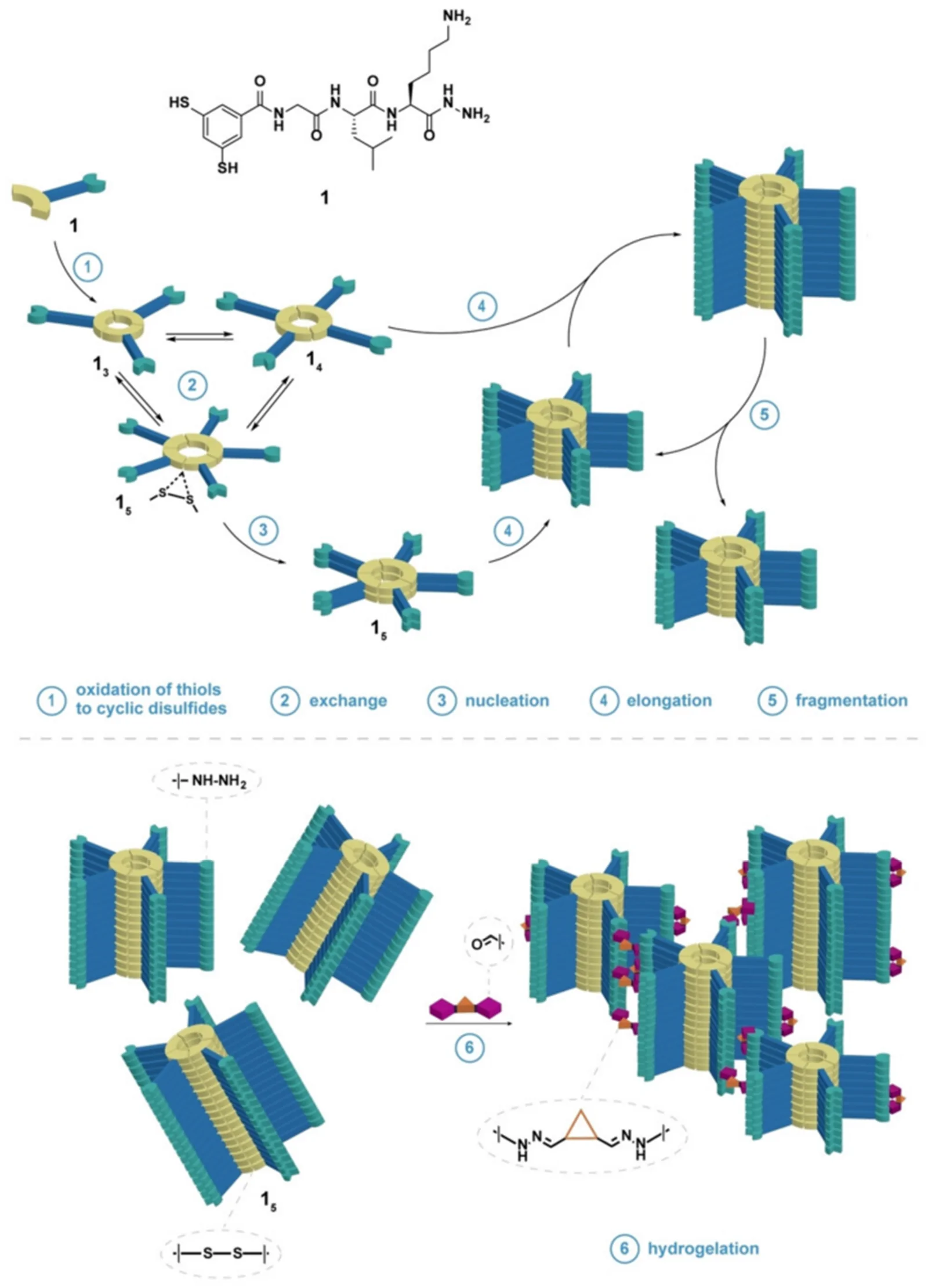
Schematic diagrams illustrating the stepwise construction of kinetically cascaded supramolecular hydrogel, with all visualized molecular and assembly elements explicitly mapped to the “fast π–π anchorage, slow dual dynamic covalent locking” synergistic self-healing mechanism. The fiber formation schematic visually presents aromatic dithiol building blocks that rely on fast reversible π–π stacking interactions for molecular aggregation, alongside thiol oxidation-driven disulfide dynamic covalent exchange. The sequential steps of oxidation, cyclic oligomer exchange, nucleation, fiber elongation, and fragmentation shown in the panel reflect the rapid supramolecular stacking as the primary fast-acting physical anchoring motif prior to covalent network formation. The hydrogelation schematic further demonstrates the secondary crosslinking step via acylhydrazone dynamic covalent bonds between pre-assembled nanofibers and dialdehyde crosslinkers. The two distinct reversible interactions (fast π–π stacking supramolecular association, slow disulfide/acylhydrazone dual dynamic covalent bonds) visualized in panels a and b form temporally coordinated cascaded repair units: π–π stacking rapidly aligns fractured fiber surfaces, while disulfide and acylhydrazone bonds gradually reconstruct stable permanent crosslinks, fully visualizing the kinetic complementary synergy between supramolecular stacking and orthogonal dual dynamic covalent bonds. Adapted with permission from Ref. [161].

Meanwhile, the acylhydrazone bonds possess typical pH-responsive characteristics. Under acidic conditions (pH 4.5), the gradual hydrolysis of acylhydrazone bonds leads to slow degradation of the gel over 4 days and upon re-addition of the aldehyde crosslinker, the complete network can be reconstructed, demonstrating reversible sol–gel transition capability. The hydrogel can be conveniently grafted with cell adhesion peptides such as RGD and LDV via the acylhydrazone bond reaction (completing functionalization within 30 min), and the modified systems can still undergo rapid gelation within 2 min. Cell experiment results showed that human bone marrow mesenchymal stem cells cultured in the gel matrix maintained a viability of nearly 100% after 24 h, with cells adhering and spreading normally, demonstrating excellent biocompatibility. With its customizable network structure, outstanding self-healing performance, environmentally responsive characteristics, and favorable cell affinity, this supramolecular hydrogel holds broad application prospects in biomedical fields such as three-dimensional cell culture, tissue engineering scaffolds, and targeted drug delivery (Figure 16). The remarkably low gelation concentration (0.025 wt%) and rapid self-healing (2–10 min gelation time) of this pseudopeptide hydrogel make it ideal for three-dimensional cell culture and injectable tissue engineering scaffolds. The synergy between π–π stacking (rapid physical anchoring) and dual dynamic covalent bonds (permanent covalent repair) enables efficient healing without external stimuli. The pH-responsive degradation (4 days at pH 4.5) allows controlled scaffold resorption, though the relatively slow degradation rate may require tailoring for specific tissue regeneration timelines.

Stimuli-responsive-oriented systems exploit the orthogonal characteristics of π–π stacking and dynamic covalent bonds responding to different external signals, endowing the hydrogel with multi-modal mechanical regulation capability. Systems containing azobenzene groups are a typical representative of this direction—UV/visible light reversibly regulates the configuration of azobenzene and the state of aromatic ring stacking, altering the physical crosslinking density, while dynamic covalent bonds respond to chemical signals such as pH or redox conditions, thereby altering the chemical crosslinking state. Light and chemical signals act on π–π stacking and dynamic covalent bonds, respectively, without mutual interference, enabling the hydrogel to achieve stepwise regulation of mechanical properties under multiple stimuli. The sensitivity of π–π stacking to temperature and solvent environment further expands the stimuli-responsive dimensions, complementing the chemical responsiveness of dynamic covalent bonds, and allowing the hydrogel to execute differentiated response behaviors to different combinations of environmental signals.

**Figure 17:**
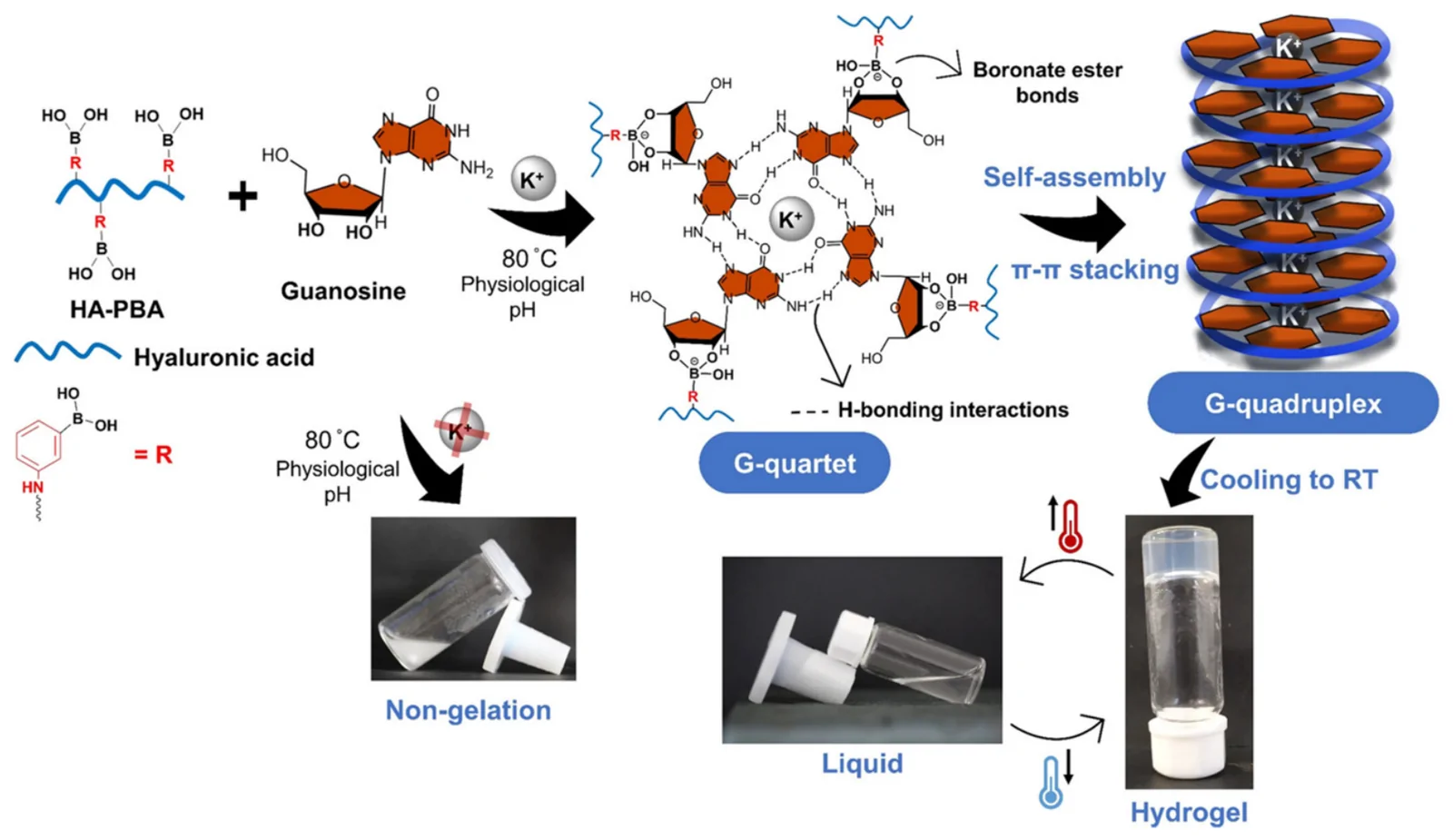
Schematic depiction of the orthogonal dual-crosslink assembly mechanism for K^+^-stabilized HA-PBA/G supramolecular hydrogel, with all visualized molecular assemblies directly mapped to the non-interfering multi-stimuli synergistic mechanism of π–π stacking and boronate ester dynamic bonds. The diagram separately illustrates two orthogonally responsive reversible interactions coexisting within one network: guanosine monomers form G-quartets via Hoogsteen hydrogen bonds, then stack into columnar backbones through temperature/K^+^-sensitive π–π supramolecular interactions, while pH-dependent boronate ester dynamic covalent linkages form between phenylboronic acid moieties on HA-PBA and vicinal diol groups of guanosine. In this schematic, upward-pointing arrow paired with red thermometer icon denotes temperature increase, and downward-pointing arrow paired with blue thermometer icon represents temperature decrease; red cross over K^+^ symbol indicates potassium-ion removal that disrupts G-quartet stacking. Visually distinguished in the graphic, the two crosslink motifs respond to fully independent environmental triggers without mutual interference: π–π stacking governs thermally reversible gel–sol transition and relies exclusively on K^+^ cation templating for stable fiber assembly, whereas boronate ester bonds serve as pH-tunable chemical crosslinks that regulate permanent network connectivity. This schematic fully visualizes the orthogonal functional division of supramolecular stacking and dynamic covalent bonds, which enable multi-modal, differentiated responsiveness to combined thermal, ionic and pH stimuli as the core synergistic feature of the hydrogel system. Reprinted with permission from Ref. [162].

For example, Vera Sousa et al. [162] developed a hyaluronic acid-functionalized G-quadruplex hydrogel exploiting orthogonal π–π stacking and boronate ester dynamic bonds. Guanosine units form G-quartets via Hoogsteen hydrogen bonding, which assemble into columnar backbones through π–π stacking (sensitive to temperature/metal ions), while boronate ester bonds between HA-PBA and guanosine vicinal diols respond specifically to pH. The two interactions respond independently to distinct signals, enabling differentiated responses to combined stimuli. Ion screening confirmed that only K^+^ (250 mM, 1.52 Å) stabilized the π–π network to form a transparent self-supporting gel without G precipitation; NH_4_^+^ formed only a loose gel with G precipitation, while Na^+^, Li^+^, Ca^2+^, Mg^2+^, and Cs^+^ all failed. Temperature sweeps showed G′ reached 9000 Pa upon cooling from 80 °C to room temperature, while reheating caused complete gel–sol transition due to hydrogen bond disruption, repeatable without compromising gel formation. pH experiments confirmed boronate ester formation was hindered at pH 4, nearly complete at pH 10, and stable at pH 7.4. Rheological cycling showed 1.5% and 2.5% hydrogels recovered ~100% of original G′ after deformation, while 3.5% showed ~95% recovery; G′ increased from 21 to 137 kPa with concentration from 1.5% to 3.5% (*w*/*v*). The hydrogel is injectable (16 G–25 G), self-heals within 30 min at RT, conductive (LED), and has 88–94% water content. Its intrinsic instability enables controlled degradation (hours at 37 °C in PBS/culture medium, 2 weeks stable at RT), allowing use as a sacrificial template to fabricate perfusable microchannels (384 ± 4 μm or 711 ± 27 μm) in GelMA matrices, enhancing nutrient/oxygen transport and cell viability for applications in 3D cell culture, organ-on-a-chip, and tissue regeneration (Figure 17). The G-quadruplex/HA hydrogel is uniquely positioned for organ-on-a-chip and vascularized tissue engineering applications, where the sacrificial template capability enables fabrication of perfusable microchannels (384–711 μm) within photo-crosslinkable matrices. The orthogonal responsiveness of π–π stacking (temperature/metal ion) and boronate ester bonds (pH) provides independent control over gelation and degradation. However, the intrinsic instability of the G-quadruplex network in physiological media (over hours at 37 °C) may limit its long-term in vivo applications.

Structure-regulation-oriented systems focus on exploiting the directionality of π–π stacking to guide the formation of network microstructures, while dynamic covalent bonds either lock these structures in place or endow them with reversible reorganization capability. Directional π–π stacking between aromatic groups can drive polymer chains to form ordered nanofibers, lamellar structures, or helical structures, imparting unique micromorphology and mechanical anisotropy to the hydrogel. The role of dynamic covalent bonds in such systems is to perform secondary crosslinking and locking of the ordered structures formed by π–π stacking, thereby fixing the supramolecular structures through covalent bonds to prevent their uncontrolled disintegration under environmental changes. Simultaneously, the reversibility of dynamic covalent bonds allows these structures to undergo topological rearrangement under specific stimuli, enabling the microstructure of the gel to reversibly switch between a thermodynamically equilibrated state and a kinetically trapped state. This synergistic mode—π–π stacking guiding ordered assembly, dynamic covalent bonds locking or regulating the assembled structures—achieves multi-scale structural control from molecular stacking to nanostructures and further to macroscopic gels.

**Figure 18:**
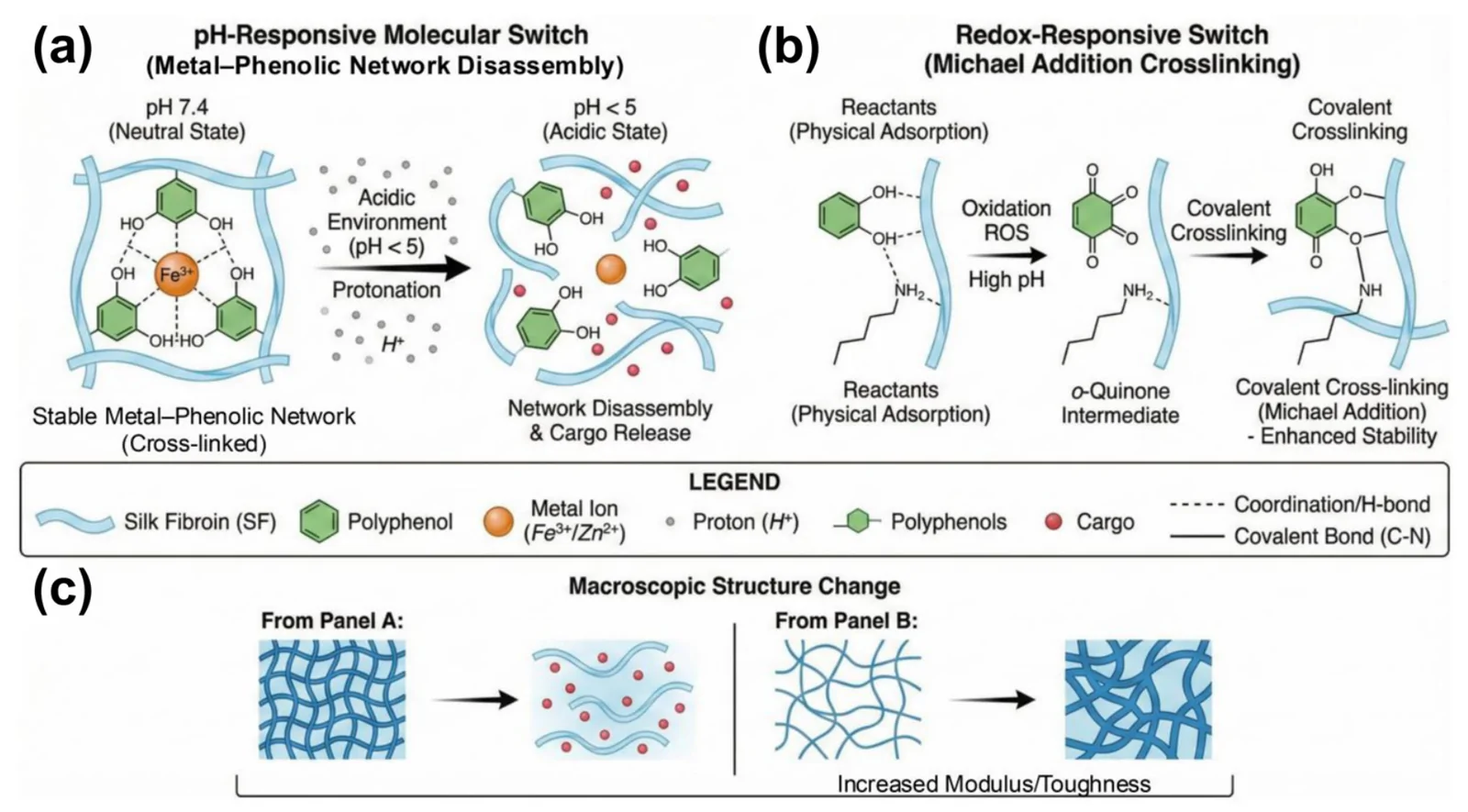
Schematic diagram illustrating the orthogonal synergistic structural regulation mechanism of directional π–π stacking and reversible dynamic crosslinks in silk fibroin (SF)-polyphenol complexes, with each subgraph’s visual elements directly correlated to the “stacking-guided assembly + dynamic bond locking” core synergistic principle. (**a**) pH-triggered disassembly schematic of metal–phenolic coordination networks: The drawing shows ordered nanofibrillar architectures pre-organized by directional aromatic π–π stacking, while metal–polyphenol dynamic coordination bonds act as secondary locking crosslinks. Acidic protonation disrupts metal–ligand coordination without fully breaking π–π stacking aggregates, triggering network dissociation and cargo release, which visually demonstrates that dynamic bonds govern stimulus-responsive topological rearrangement while π–π stacking maintains partial ordered skeletons. (**b**) Redox-activated quinone-mediated Michael addition covalent crosslinking pathway: The graphic displays polyphenol aromatic rings forming pre-assembled ordered chains via π–π stacking; oxidative redox stimuli convert phenols to quinone intermediates, which generate permanent dynamic covalent linkages to lock the π–π stacked ordered microstructures, preventing disordered disassembly of supramolecular assemblies under environmental fluctuations. (**c**) Macroscopic morphological comparison chart: The left disassembly model corresponds to pH-induced cleavage of metal–phenolic dynamic crosslinks (retaining weak π–π stacking association for loose networks), while the right reinforced network arises from redox-driven covalent locking on π–π stacked templates, visually contrasting the two distinct macroscopic outcomes originating from the orthogonal functional division of π–π stacking (ordered microarchitecture template) and dynamic crosslinks (reversible structural lockers). Collectively, the figure visualizes the multi-scale synergistic regulation logic: directional π–π stacking dictates the intrinsic nanofibrillar ordered morphology, and diverse dynamic crosslinks enable reversible locking or stimulus-triggered structural reorganization. Reprinted with permission from Ref. [163].

For example, Ma et al. [163] conducted a systematic review of research on silk fibroin-polyphenol gels and hydrogels. This system is a typical structure-regulation-oriented hydrogel platform, relying on the synergistic effect of directional π–π stacking and dynamic crosslinking networks to achieve precise multi-scale structural control, fully conforming to the mode of action in which π–π stacking guides ordered assembly and dynamic covalent bonds lock and regulate the assembled structures. In this system, the aromatic groups on silk fibroin molecular chains and polyphenols form strongly directional π–π stacking interactions, driving protein chains to arrange in an orderly manner and to autonomously construct ordered microscopic morphologies, such as nanofibers and lamellar-like structures. The dynamic crosslinking structures within the system, including enzymatic covalent bonds, metal–polyphenol coordination networks, and oxidative crosslinking, serve as secondary crosslinking sites that complete structural locking of the ordered framework formed by π–π stacking, effectively preventing supramolecular assemblies from disintegrating under environmental changes, while the reversible characteristics of dynamic covalent bonds enable responses to external stimuli such as pH, redox conditions, temperature, and light, driving topological rearrangement of the gel microstructure and allowing the system to switch between thermodynamically equilibrated and kinetically trapped states reversibly. Rheological and microscopic characterizations have demonstrated that the synergistic interplay between π–π stacking and dynamic crosslinking enables precise tuning of the hydrogel’s storage modulus and compressive strength across a wide range, while also directing the formation of uniform nanofibrillar networks with tunable density. Besides structural regulation, the system also integrates excellent wet adhesion, intrinsic self-healing capability, multiple stimulus responsiveness (pH/redox/temperature/light), tunable enzyme-mediated degradation, good biocompatibility, and broad application prospects in bioadhesives, wound dressings, flexible bioelectronics, and drug delivery platforms (Figure 18). The structure-regulation capability of this silk fibroin-polyphenol platform enables precise control over nanofibrillar network morphology, making it a versatile system for applications ranging from bioadhesives to flexible bioelectronics. The directional π–π stacking drives ordered assembly of silk fibroin chains, while dynamic crosslinks (enzymatic, metal–phenolic, and oxidative) lock these structures in place. This hierarchical control over microstructure-to-macroscopic properties is particularly valuable for tissue engineering scaffolds, where mechanical anisotropy and bioactivity are simultaneously required.

In summary, the three functional orientations of π–π stacking and dynamic covalent bond systems represent parallel strategies rather than a sequential progression. Self-healing-oriented systems prioritize rapid repair kinetics and high healing efficiency, making them suitable for applications where mechanical recovery is the primary requirement. Stimuli-responsive-oriented systems prioritize multi-modal responsiveness and stepwise mechanical regulation, making them suitable for applications requiring programmable deformation or controlled actuation. Structure-regulation-oriented systems prioritize the construction of ordered microstructures and mechanical anisotropy, making them suitable for applications in tissue engineering and biomimetic materials.

Looking ahead, several critical directions merit particular attention for advancing synergistic π–π stacking/DCB hydrogels. First, the integration of the three functional orientations into unified systems remains largely unexplored. For example, self-healing systems with structure-regulating capability could combine rapid repair with anisotropic mechanical properties, while stimuli-responsive systems with enhanced mechanical performance could enable programmable actuation under load-bearing conditions. Achieving such integration will require precise kinetic matching between π–π stacking and dynamic covalent bonds to ensure that fast interfacial recognition, intermediate structural reorganization, and slow permanent repair operate in a coordinated cascade. Second, incorporating carbon nanomaterials (e.g., graphene, carbon nanotubes) and two-dimensional materials (e.g., MXene) via π–π stacking interactions represents a particularly promising direction for extending this field from molecular aromatic systems to nanomaterial-enhanced systems. Beyond electrical conductivity and photothermal conversion, such integration could introduce electromagnetic interference shielding, electrochemical energy storage, and biosensing functionalities, dramatically expanding the application scope. However, the trade-off between nanomaterial loading and gel processability must be carefully managed to avoid compromising the dynamic reversibility that defines these systems. Third, despite the demonstrated potential of π–π stacking for constructing ordered nanofibers and helical structures, translating these ordered microstructures into predictable macroscopic mechanical properties remains a significant challenge. Establishing quantitative structure–property relationships that connect molecular-level aromatic stacking to bulk-scale mechanical anisotropy, toughness, and fatigue resistance will be essential for rational material design. Fourth, the long-term stability of π–π stacking-based hydrogels under physiological conditions—including exposure to proteins, lipids, and mechanical fatigue—has not been systematically evaluated. Systematic in vivo studies assessing degradation kinetics, host tissue integration, and immune responses are urgently needed before clinical translation can be considered. Progress along these directions will be essential to unlock the full potential of π–π stacking/DCB synergistic hydrogels for self-healing tissue engineering scaffolds, photothermal conversion devices, and intelligent actuation systems.

### 3.7. Hierarchical and Emerging Multiple Dynamic Systems

**Figure 19:**
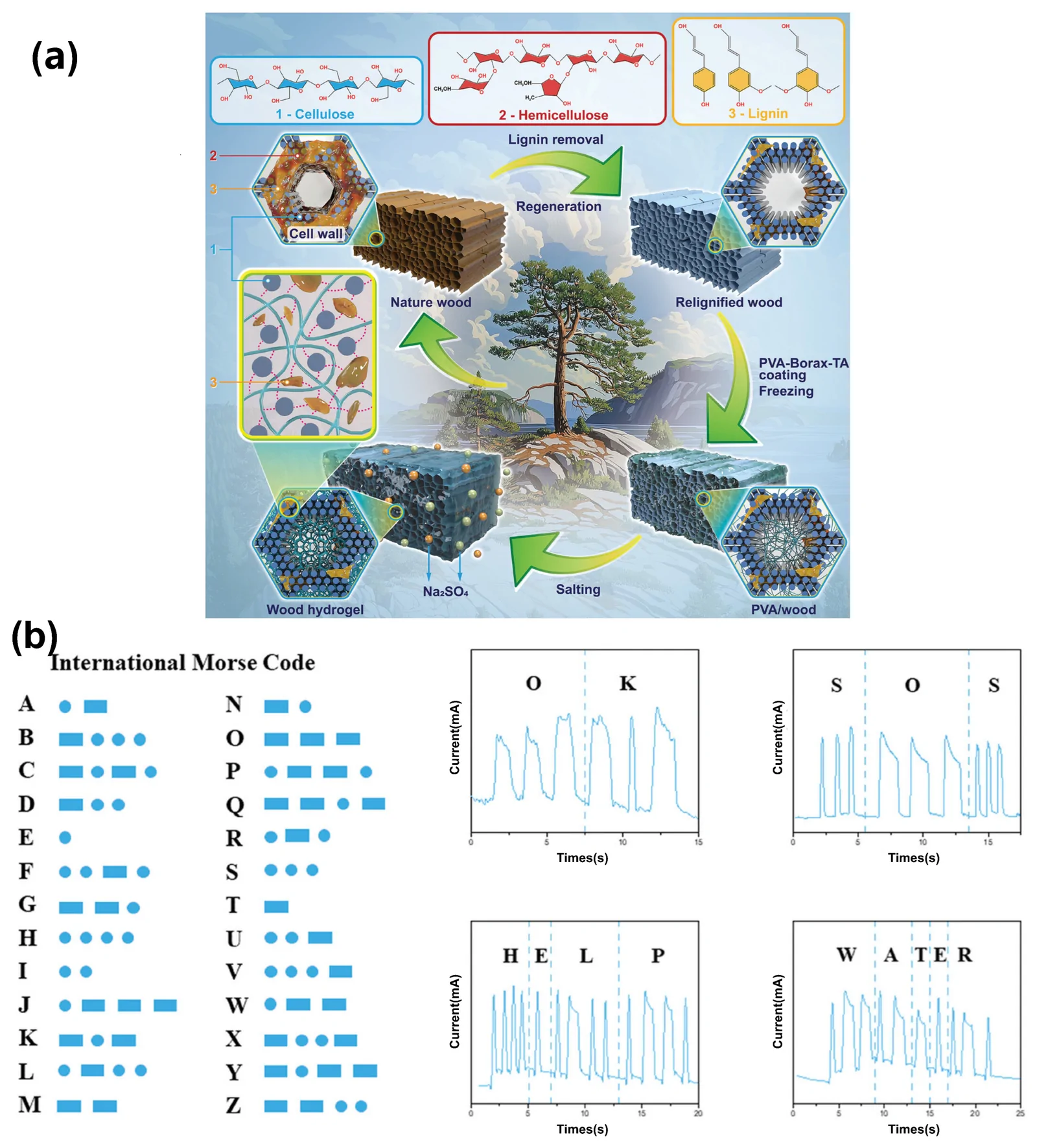
Composite schematic and functional demonstration of wood-derived WPBTS anisotropic hydrogel, with all graphical elements explicitly correlated to the hierarchical synergistic energy-dissipation mechanism combining multiple non-covalent interactions and boronate ester dynamic covalent bonds. (**a**) Synthetic schematic illustrating the fabrication workflow of WPBTS hydrogel: The diagram visualizes the coexistence of three orthogonal reversible crosslink motifs within one cellulose-supported network—intermolecular hydrogen bonds between cellulose/PVA hydroxyl groups, hydrophobic aggregation of tannic acid aromatic segments, and reversible boronate ester dynamic covalent linkages formed by borate ions. These multi-type dynamic interactions act synergistically to construct a hierarchically crosslinked framework that delivers broadband tissue-mimicking energy dissipation, as the schematic’s multi-scale molecular assembly directly reflects the hybrid supramolecular + covalent network design described in the mechanistic section. (**b**) Underwater signal sensing performance of WPBTS hydrogel: The real-time current response curves for Morse code transmission demonstrate the functional outcome of the multi-bond synergistic architecture. The combined hydrogen bonding, hydrophobic forces and boronate ester bonds jointly maintain stable conductive ionic pathways under repeated deformation and aqueous environments; the layered dynamic crosslink system enables rapid structural recovery after strain, supporting reliable, continuous electrical signal output for underwater information encoding, fully embodying the enhanced mechanical stability and environmental adaptability brought by multi-interaction synergistic energy dissipation. Reprinted with permission from Ref. [164].

Beyond the six principal strategies discussed above—each centered on a single type of non-covalent interaction combined with dynamic covalent bonds—researchers have also explored more complex and sophisticated architectures that extend beyond this binary framework. These advanced systems can be broadly categorized into three tiers of increasing complexity:

Tier 1: Multiple Non-Covalent Interactions with Dynamic Covalent Bonds—Some systems integrate two or more types of non-covalent interactions (e.g., hydrogen bonds + hydrophobic interactions, or hydrogen bonds + π–π stacking + metal coordination) with dynamic covalent bonds within a single network. These hybrid systems are designed to achieve more refined hierarchical energy dissipation and broader stimuli-responsiveness than any single non-covalent strategy alone. For instance, Teng et al. [164] integrated hydrogen bonds, hydrophobic interactions, and boronate ester dynamic covalent bonds into a wood-based anisotropic hydrogel, enabling the gel to exhibit biological tissue-like damping characteristics across a wide frequency range (Figure 19). Similarly, Li et al. [165] combined hydrogen bonds, electrostatic interactions, hydrophobic interactions, and disulfide bonds in a protein/polysaccharide interpenetrating network, achieving multiple orthogonal stimuli-responsiveness and tunable mechanical properties.

**Figure 20:**
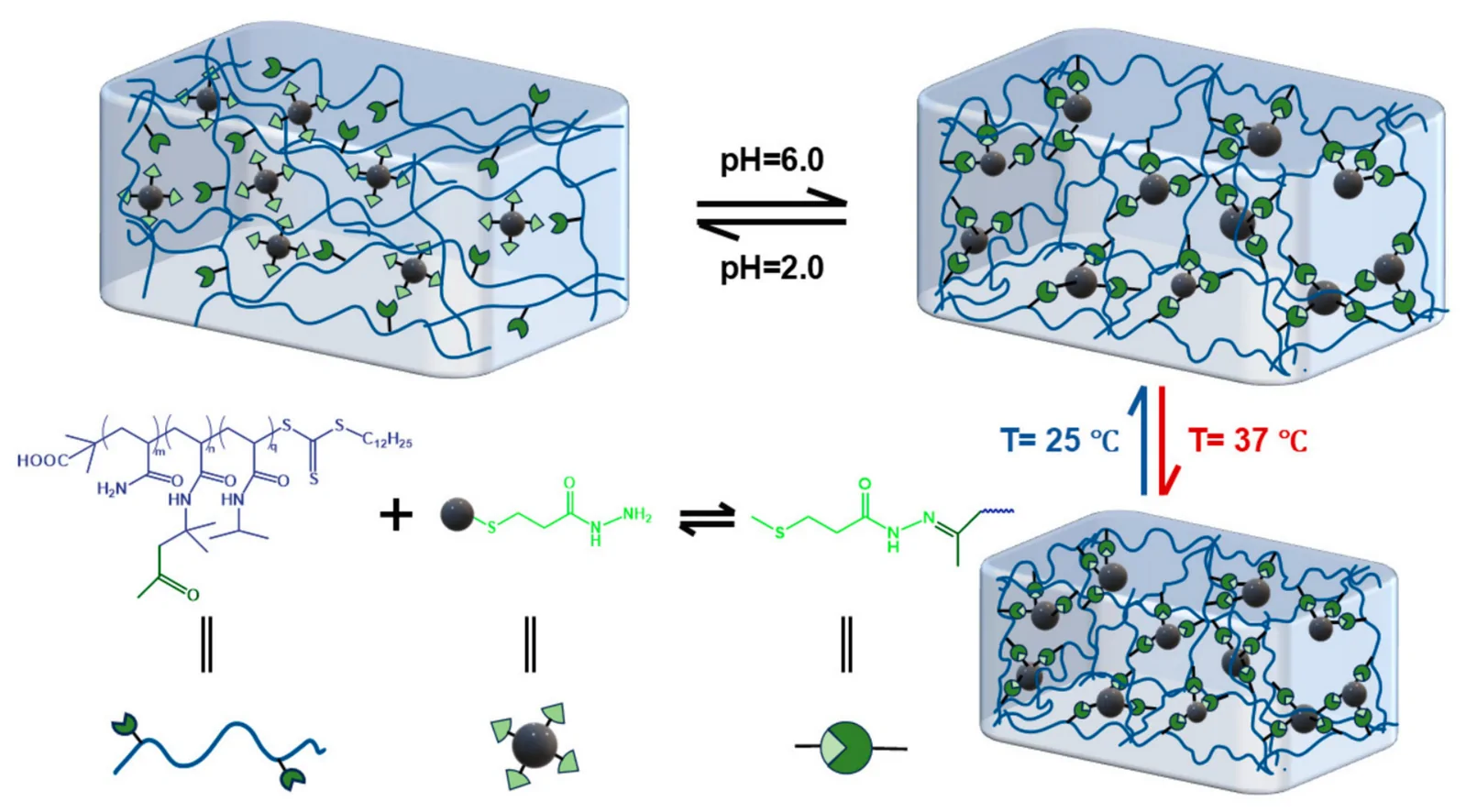
Schematic fabrication route for a multifunctional conductive hydrogel crosslinked by hydrazide-modified silver nanoparticles (AgNPs); the single unified molecular assembly graphic visualizes the synergistic mechanism of nanoparticle crosslinking nodes and acylhydrazone dynamic covalent bonds. The whole scheme explicitly presents that surface-functionalized AgNPs act as multi-effect crosslinking units: hydrazide groups on AgNP surfaces form reversible acylhydrazone dynamic covalent linkages with ketone groups of thermoresponsive poly(NIPAM-co-DAAM-co-AM) chains to build the primary dynamic network. As multifunctional nano-fillers, AgNP cores synergistically introduce continuous conductive channels and sustained antibacterial silver ion release, while the dynamic acylhydrazone framework provides self-repair, pH-responsive sol–gel switching and thermally tunable polymer chain conformation, jointly realizing integrated strain/temperature wearable sensing, conductivity and bacteriostasis—consistent with the mechanism that nanoscale fillers form multi-interfacial dynamic connections with polymer matrix to simultaneously strengthen mechanical properties and append brand-new electrical/biological functional dimensions. Reprinted with permission from Ref. [166].

Tier 2: Nanomaterial-Enhanced Multiple Dynamic Systems—Surface-functionalized nanoparticles—such as gold nanoparticles, nanocellulose, MXene, silver nanowires, and metal-organic frameworks—can be embedded as multifunctional crosslinking nodes into the dynamic network, forming multiple dynamic interfacial connections with the polymer matrix through dynamic covalent bonds and various non-covalent interactions. These nanomaterials not only act as reinforcing fillers that significantly enhance modulus and toughness but also, by virtue of their intrinsic electrical, magnetic, photothermal, or catalytic properties, endow the hydrogel with entirely new functional dimensions. For example, Qi et al. [166] incorporated hydrazide-functionalized silver nanoparticles into a thermo-responsive copolymer network through dynamic acylhydrazone bonds, endowing the hydrogel with high conductivity (6.85 S/m), antimicrobial activity, and dual strain/temperature sensing capabilities for wearable electronics and health monitoring (Figure 20). Qin et al. [167] utilized gold nanoparticles as multifunctional crosslinkers via dynamic Au–thiolate coordination to construct a highly branched nanocomposite hydrogel, achieving exceptional mechanical strength (>1 MPa), notch insensitivity, and rapid near-infrared-triggered self-healing with ~96% efficiency in 1 min for potential biomedical applications.

**Figure 21:**
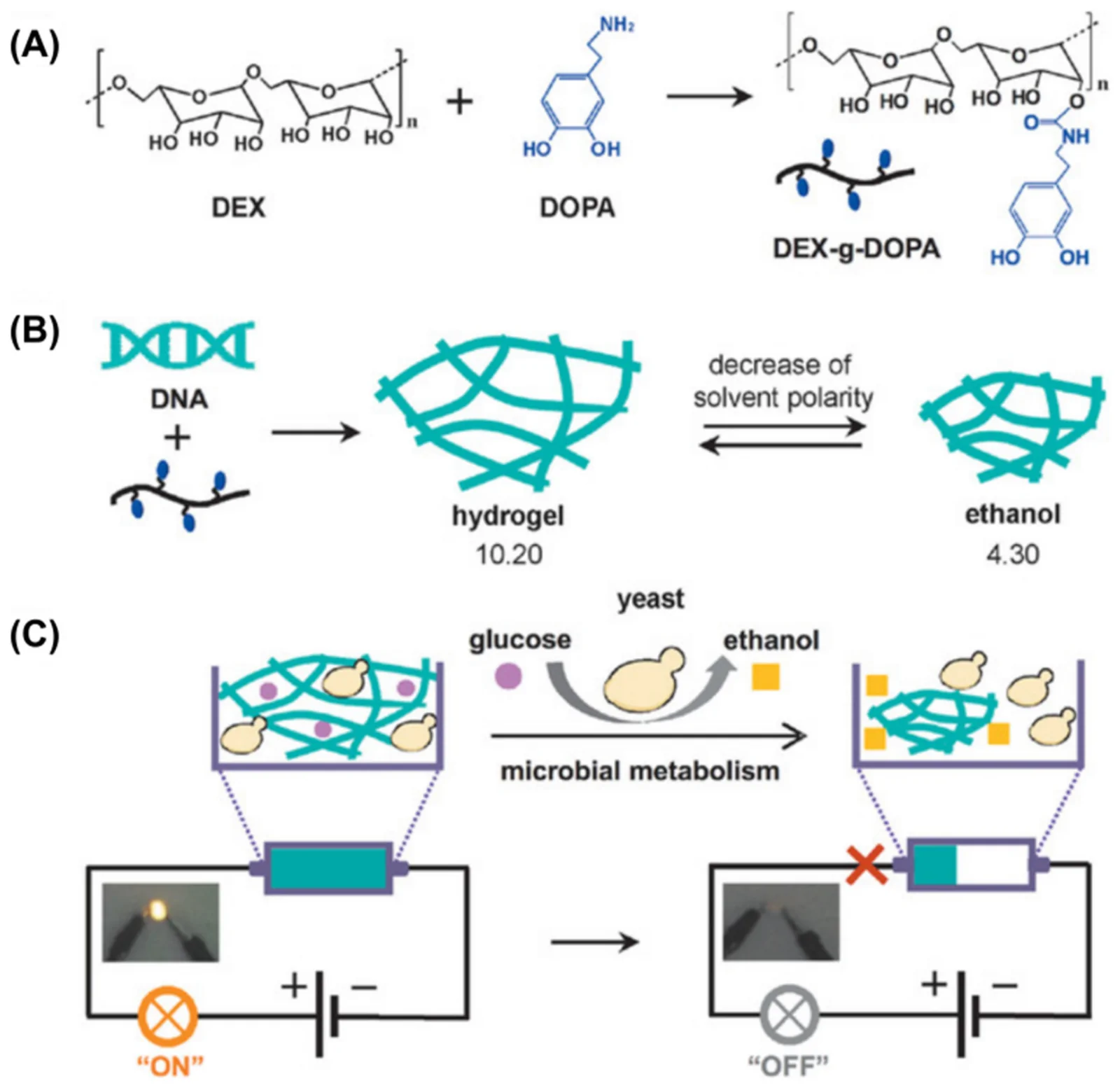
Schematic molecular design, fabrication and bio-responsive circuit demonstration of biogenic dual-crosslink DNA/DEX-g-DOPA hydrogel, with each subgraph explicitly correlated to the synergistic mechanism of biological macromolecular dynamic interactions and synthetic dopamine-based dynamic covalent bonds. (**A**) Synthetic pathway of dopamine-grafted dextran (DEX-g-DOPA): The molecular graphic visualizes the introduction of catechol dopamine motifs onto dextran backbones, which provide synthetic dynamic covalent crosslinking sites (DOPA-DOPA oxidative covalent bonding) to match the biological dynamic interactions of DNA chains, establishing the dual biogenic-synthetic crosslink precursor for the hybrid network. (**B**) Assembly process of nanofibrous DNA/DEX-g-DOPA hydrogel and its solvent polarity-triggered volumetric switching behavior: This panel illustrates the formation of synergistic dual networks: ultra-long DNA chains form biological dynamic physical entanglements and base stacking supramolecular interactions as the biological crosslinking component, while DOPA-mediated hydrogen bonds, π–π stacking and permanent oxidative covalent linkages serve as synthetic dynamic crosslinks. The orthogonal two types of dynamic interactions jointly endow the hydrogel with ultra-soft mechanical properties and rapid, reversible volume shrinkage/swelling upon variation in solvent polarity, thereby enabling biocompatible environmental responsiveness unavailable in fully synthetic hydrogel systems. (**C**) Proof-of-concept demonstration of microbial metabolism-activated electrical circuits using the dual-crosslink hydrogel as conductive dynamic wires: The graphic shows the functional output originating from the biogenic-synthetic bond synergy; yeast fermentation produces ethanol to alter medium polarity, which sequentially modulates the DNA supramolecular stacking and DOPA-based dynamic crosslink density, triggering reversible hydrogel volume change to switch the circuit on/off. The red cross mark denotes the disconnection of conductive hydrogel wire and circuit turn-off state. This bio-triggered logical responsiveness is uniquely enabled by the combination of DNA-based molecular recognition dynamics and synthetic dopamine dynamic covalent chemistry, highlighting the superior biocompatibility and biological signal-response capacity of hybrid biodynamic hydrogel platforms. Reprinted with permission from Ref. [168].

Tier 3: Biological Macromolecule Dynamic Systems—Moving beyond purely synthetic systems, biological macromolecule dynamic crosslinking systems utilize biologically derived dynamic bonds—such as DNA base pairing, antigen–antibody binding, and enzyme-responsive peptide segments—in combination with synthetic dynamic covalent bonds. These systems achieve levels of molecular recognition specificity, biocompatibility, and logical responsiveness that are difficult for traditional fully synthetic systems to reach. For example, Han et al. [168] constructed a DNA/dopamine-grafted dextran hydrogel that responds to solvent polarity and microbial metabolites, enabling the construction of electric circuits controlled by microbial metabolism (Figure 21). Yang et al. [169] developed a DNA-based hydrogel co-assembled from amyloid fibers and clay nanosheets, achieving injectability, self-healing, and 3D printability for vascularized bone regeneration.

It is important to note that these three tiers are not a strict progression; rather, they represent different levels of architectural complexity that can be combined in various ways. Tier 1 introduces multiple non-covalent synergies; Tier 2 adds nanomaterials as functional crosslinking nodes; Tier 3 incorporates biological macromolecules for unparalleled bio-adaptability. The boundaries between these tiers are increasingly blurred, and future systems are likely to integrate elements from all three tiers—for instance, nanomaterial-enhanced, biologically functionalized multi-non-covalent dynamic networks.

To provide readers with a quantitative overview of the mechanical performance and self-healing capabilities across the six combination strategies discussed above, we summarize the key metrics of representative systems in Table 3. This comparison allows for a direct assessment of performance trade-offs: hydrogen-bond-based systems excel in stretchability and rapid room-temperature healing, metal–ligand systems offer the highest mechanical strength and programmable stiffness, host–guest systems provide exceptional extensibility and modular responsiveness, electrostatic systems deliver high ionic conductivity and biocompatibility, hydrophobic systems combine strong adhesion with pH-responsive drug release, and π–π stacking systems enable low-concentration gelation with stimuli-responsive microstructural control. Such cross-strategy comparison highlights the importance of selecting appropriate dynamic bond combinations based on specific application requirements.

## 4. Conclusions and Recommendations

The strategy of multiple dynamic bond crosslinking, through the synergistic assembly of non-covalent interactions and dynamic covalent bonds within a single hydrogel network, has systematically overcome multiple bottlenecks of traditional hydrogels, such as weak mechanical strength, low self-healing efficiency, single-mode stimuli-responsiveness, and poor processability, thereby opening up vast opportunities for creating a new class of soft materials with tissue-like mechanics and intelligent environmental responsiveness. Focusing on the types of non-covalent interactions, this review has systematically elucidated the construction logic, microscopic mechanisms, and application scenarios of synergistic systems combining dynamic covalent bonds with four categories of non-covalent forces: hydrogen bonds, host–guest recognition and metal coordination, ionic bonds, and hydrophobic interactions and π–π stacking. Hydrogen bonds, due to their ubiquity and rapid reversibility, have become a “universal module” for combination with various dynamic covalent bonds, demonstrating application potential first in self-healing sensors and biolubrication. Host–guest recognition provides molecular-level precise crosslinking and orthogonal regulation, offering a platform for programmable release and logic-responsive materials. Metal coordination provides tunable, high-energy sacrificial bonds and multifunctionality, significantly enhancing the mechanical and biological performance of hydrogels. Ionic bonds exhibit dual-factor regulatory capabilities and intrinsic ionic conductivity, offering clear advantages in 3D printing, ionic skins, and gastrointestinal drug delivery. The temperature-enhancement and aggregation-induced conductivity characteristics of hydrophobic and π–π stacking interactions introduce unique functionalities into shape-memory, underwater adhesion, and photo-controlled intelligent devices. Furthermore, frontier directions such as hierarchical composites and bio-hybrid systems are continuously expanding the functional boundaries of multiple dynamic bond-crosslinked hydrogels.

Nevertheless, the field still faces a series of core challenges on its path toward practical application. The first is the insufficient quantitative understanding of structure–property relationships: current knowledge of the coupled exchange kinetics and cross-scale synergistic mechanisms of multiple dynamic bonds within complex networks remains at the level of qualitative descriptions and phenomenological summaries. There is an urgent need to develop theoretical models capable of cross-scale simulation, from molecular dynamics to continuum mechanics, to guide the rational design of materials. The second is the test of long-term stability and durability: although the reversibility of dynamic bonds endows materials with short-term self-healing capability, under demanding conditions in long-term use, particularly full immersion, repeated large strains, and extreme temperatures, the network may undergo slow irreversible dissociation or mechanical aging. Anti-fatigue and anti-drying properties require systematic optimization. Biosafety and clinical translation are also critical hurdles that cannot be ignored: most systems still lack complete long-term in vivo biological evaluation data, and key information such as the metabolic pathways and immunogenicity of degradation products remains to be supplemented. The pathway from laboratory-scale synthesis to large-scale, regulation-compliant production must also be established. The inherent contradictions among multiple functionalities—such as the trade-offs between high water content and high strength, high ionic conductivity and mechanical robustness, and rapid self-healing and high modulus—still need to be resolved through more sophisticated network topology design and interfacial engineering.

Looking ahead, translating multiple dynamic bond-crosslinked hydrogels from laboratory prototypes to practical technologies requires concerted efforts across several actionable fronts.

First, AI-driven materials discovery must evolve from brute-force screening to knowledge-guided design. By training machine learning models on existing datasets of dynamic bond kinetics (e.g., bond exchange rates, activation energies, and stimuli-response thresholds) and corresponding hydrogel properties (e.g., mechanical strength, healing efficiency, and degradation profiles), researchers can predict optimal bond combinations and crosslinking densities for target applications without exhaustive experimental iteration. This approach is particularly promising for navigating the vast combinatorial space of multiple dynamic bonds, where the number of possible pairings far exceeds what can be empirically tested.

Second, 4D bioprinting offers a transformative platform for encoding the temporal responsiveness of multiple dynamic bonds into spatially patterned constructs. By leveraging the orthogonal stimuli-responsiveness of different bond types—for instance, photo-responsive host–guest crosslinks for spatial patterning and pH-responsive Schiff base bonds for temporal evolution—printed structures can undergo programmed deformation, functional differentiation, or staged degradation post-printing. This capability opens new paradigms for fabricating dynamic tissue scaffolds that evolve with regenerating tissues.

Third, clinical translation demands systematic evaluation beyond acute biocompatibility. Key challenges include long-term in vivo stability under mechanical loading and enzymatic degradation, sterilizability without compromising dynamic bond functionality, and scalable manufacturing under Good Manufacturing Practice (GMP) compliance. Establishing standardized characterization protocols for dynamic bond behavior in physiologically relevant conditions—including dynamic mechanical analysis under cyclic loading and in situ bond exchange monitoring—will be essential for regulatory approval.

Fourth, green and circular chemistry principles should guide the selection of renewable raw materials, aqueous-phase synthesis routes, and degradable or recyclable network designs. The reversibility inherent to dynamic bonds offers a unique advantage for closed-loop material lifecycles, enabling depolymerization and re-polymerization without loss of functionality.

Fifth, theranostic platforms will benefit from integrating multiple dynamic bonds with diagnostic and therapeutic modules within a single network. For example, metal-coordinated hydrogels with bioactive ions can combine real-time imaging contrast (via magnetic or photoacoustic properties) with on-demand therapeutic release triggered by pathological signals, forming closed-loop systems that integrate disease monitoring, treatment, and therapeutic feedback.

It is foreseeable that with the accelerating convergence of dynamic covalent chemistry, supramolecular chemistry, polymer physics, and information science, multiple dynamic bond-crosslinked hydrogels will continue to evolve toward more refined structures, more integrated functions, and more intelligent responsiveness, and are bound to have a profound impact on many fields including intelligent medicine, flexible electronics, soft robotics, and sustainable materials.

Returning to the challenges articulated in the Introduction—insufficient mechanical strength, slow and inefficient self-healing, limited environmental responsiveness, and the lack of multifunctional integration—the strategies presented in Section 3.1, Section 3.2, Section 3.3, Section 3.4, Section 3.5, Section 3.6 and Section 3.7 collectively demonstrate how multiple dynamic bond crosslinking has systematically addressed these limitations. As summarized in Table 1, each challenge has been tackled by one or more combination strategies, with the field progressively advancing from single-bond systems through dual-bond synergies to the hierarchical and emerging systems discussed in Section 3.7. The journey from basic principles (Section 2) to strategy implementation (Section 3) and finally to applications and future directions (Section 4) reveals a clear trajectory: the field is moving toward increasingly sophisticated systems where mechanical performance, dynamic reversibility, environmental responsiveness, and biological functionality are not merely coexisting but are synergistically integrated into a single, programmable network. While significant challenges remain—particularly regarding long-term stability, biosafety, and the resolution of trade-offs among multiple functionalities—the foundational principles and design strategies established in this review provide a robust framework for the continued development of intelligent, multifunctional hydrogel materials.

## Figures and Tables

**Table 1:** Mapping of key challenges to the six combination strategies.

Key Challenge	Corresponding Strategy (Section)	Representative Dynamic Bond Combination
Insufficient mechanical strength	DCBs + metal–ligand/hydrophobic interaction	Fe^3+^-catechol/disulfide; hydrophobic association/Schiff base
Slow self-healing rate	DCBs + hydrogen bonds/host–guest interaction	Hydrogen bonds/Schiff base; β-CD/adamantane/acylhydrazone
Low healing efficiency	DCBs + π–π stacking/metal–ligand interaction	π–π stacking/disulfide; Ca^2+^-carboxyl/disulfide
Lack of environmental stimuli-responsiveness	DCBs + electrostatic/host–guest interaction	Polyelectrolyte/boronate ester; α-CD/azobenzene/imine
Poor electrical conductivity	DCBs + electrostatic/π–π stacking interaction	Zwitterionic/disulfide; graphene/dynamic bonds
Limited functional integration	DCBs + metal–ligand/π–π stacking interaction	Bioactive ions/Schiff base; carbon nanomaterials / DCBs
Weak interfacial/tissue adhesion	DCBs + hydrogen bonds/hydrophobic interaction	PU hard-segment/acylhydrazone; urushiol/boronate ester

**Table 2:** Quantitative comparison of host–guest pairs for dynamic hydrogel construction.

Host–Guest Pair	Binding Constant (K_a_)	Primary Stimulus	Response Timescale	Key Feature
β-CD/Adamantane	10^4^–10^5^ M^−1^	Competitive guest (e.g., 1-adamantanol)	Minutes to hours	High physiological stability; injectable hydrogels [125,144]
α-CD/Azobenzene	~2.73 × 10^4^ M^−1^	UV/Vis light (trans–cis isomerization)	Seconds to minutes	Photo-reversible; spatiotemporal control [126]
CB[8]/bis-guest	~10^13^ M^−2^ (K_eq_)	Chemical displacement	Seconds to minutes	Ultra-high affinity; ternary complex; hierarchical energy dissipation [127]
β-CD/Ferrocene	10^3^–10^4^ M^−1^	Redox (electrochemical oxidation/reduction)	Seconds	Electrochemically reversible; cyclable; conductive [145]

Notes: K_a_ values are association constants reported in the corresponding references. For CB[8]/bis-guest, K_eq_ represents the equilibrium constant for ternary complex formation (1:2 host–guest stoichiometry). Response timescales are approximate and depend on specific experimental conditions (e.g., light intensity, redox potential, guest concentration).

**Table 3:** Representative multiple dynamic hydrogels: key mechanical properties and self-healing performance across different combination strategies.

Strategy	Representative System (Ref.)	Dynamic Bond Combination	Mechanical Property	Self-Healing Performance	Functionality Highlights
DCBs + H-bonds	SH-WPU/PAMAM DN [128]	Disulfide + acylhydrazone + H-bonds	Tensile: 0.77 MPa; swelling: 354%	87.35% (48 h, RT); 31.58% (1 h)	Multi-environment adaptability
	D_x_A_y_WPU Elastomer [141]	Oxime-carbamate + H-bonds (hard segments)	Tensile: 33.04 MPa; elongation: ~955%; toughness: 90.66 MJ/m^3^	90.1% (24 h, 80 °C)	Wearable strain sensor (CNT composite): highest tensile strength
	LA-XEA Ionogel [130]	Disulfide + H-bonds + Bmim^+^–O coordination	Elongation: ~1500%	98.92% (12 h, RT)	Bio-based; wearable ionogel: highest healing efficiency
DCBs + Host–Guest	PEG/CB[8] Hydrogel [127]	Acylhydrazone + CB[8]–guest ternary complexes	Elongation: ~7000%; modulus: ~50 kPa (fast rate); *τ*_R_: 6.6 s	—	Movable supramolecular cores; rate-dependent mechanics: highest extensibility
	PVA/PN/P(β-CD) DN [145]	Boronate ester + β-CD/Fc	Fracture stress: 41.0 kPa; strain: 436%; adhesion: 11.1 kPa	95.3% (5 min, RT); healed stress: 39.1 kPa	Strain sensor (GF = 5.9); conductive
DCBs + Metal–Ligand	Poly(DMAm-AA-tPy) [124]	Transient anhydride + M^2+^–terpyridine	Peak stress: 120 kPa (EDC-fueled); ΔG′: 17 kPa (Ni^2+^)	100% (24 h, Ni^2+^); Peak recovery: 400% (1 h)	Rewritable stiffness patterns (3 levels); programmable
	PL-Cat/Fe/ODex [147]	Schiff base + Fe^3+^–catechol	Compressive: 80 kPa; adhesion: 15 kPa (porcine skin)	Compressive recovery: 91%	Injectable; pH-responsive dissolution; wound closure
	CSp-OxD@Cu^2+^/G [148]	Schiff base + boronate ester + Cu^2+^–phenolic	Compressive: withstands ~150 kPa, 80% strain	Injectable; self-healing	pH-responsive drug release; immunomodulatory; diabetic wound healing
DCBs + Electrostatic	Poly(PT)/PVA/p-MXene [155]	Disulfide + H-bonds + electrostatic + Al^3+^ coord.	Stress: 126.70 kPa; strain: 881.6%; conductivity: 1.24 S/m	Resistance recovery: 2.2 s (3 cycles)	EMI shielding: 48.56 dB; strain sensor (GF = 1.05): highest conductivity
	pLACB-LAHA Zwitterionic [156]	Disulfide + zwitterionic self-association	Compressive: 3.86 MPa; modulus: 0.25 MPa; toughness: 0.32 MJ/m^3^	—	Antioxidant (>90% DPPH); probiotic protection (91.3% viability): highest compressive strength
DCBs + Hydrophobic	QAF Hydrogel [157]	Schiff base + Fe^3+^ coord. + hydrophobic domains	BCI: 0.44%; blood loss: 21.67 mg (vs. 133.40 mg control)	30 s (body temp.); G′/G″ recovery from 200% strain	Antibacterial (>98%); wound healing (66.45% in 7 days): fastest healing
	CA/AF Micellar [159]	Schiff base + hydrophobic micelles (F127)	G′ tunable (7.2%→9.0% AF127); adhesion withstands 10 g load	Shear-thinning; injectable; network recovery after cyclic strain	pH-responsive release (41.34% at pH 6.0); wound healing (97.66% in 14 days)
DCBs + π–π Stacking	Pseudopeptide Hydrogel [161]	Disulfide + acylhydrazone + π–π stacking	Gelation: 0.025 wt%; gelation time: 2–10 min	Modulus recovery after large strain	3D cell culture (viability ~100%); pH-responsive (pH 4.5 degradation): lowest gelation concentration
	G-quadruplex/HA [162]	Boronate ester + π–π stacking (G-quartets)	G′: 21–137 kPa (1.5–3.5% *w*/*v*); water content: 88–94%	~100% G′ recovery; 30 min (RT)	Thermoresponsive; injectable (16G–25G); sacrificial template for microchannels

Note: Values are extracted from the original references. “—” indicates data not reported in the corresponding reference. DCBs: dynamic covalent bonds; RT: room temperature; GF: gauge factor; EMI: electromagnetic interference; BCI: blood clotting index; G′: storage modulus.

## Data Availability

No new data were created or analyzed in this study. Data sharing is not applicable to this article.

## Abbreviations

AAm	Acrylamide
AAPBA	(3-Acrylamidophenyl) Boronic Acid
AFPBA	4-(2-Acrylamidoethylcarbamoyl)-3-fluorophenylboronic Acid
AgNWs	Ag Nanowires
AHAMA	Aldehyde-modified Hyaluronic Acid Methacrylate
Alg-PBA	Phenylboronic Acid-grafted Sodium Alginate
Al^3+^-TA	Al^3+^ and Tannic Acid
AM	Acrylamide
APS	Ammonium Persulfate
Azo	Azobenzene
BAL	2,3-Dimercapto-1-propanol
bFGF	basic Fibroblast Growth Factor
BIS	N,N′-Methylenebisacrylamide
Bmim^+^	1-Butyl-3-methylimidazolium
BSA	Bovine Serum Albumin
BV/TV	Bone Volume Fraction
CMCS	Carboxymethyl Chitosan
CXB	Celecoxib
DA	Diels–Alder
DAAm	Diacetone Acrylamide
DCBs	Dynamic Covalent Bonds
DEX-g-DOPA	Dopamine-grafted Dextran
DH	Degree of Hysteresis
DMSA	*Meso*-2,3-dimercaptosuccinic Acid
DN	Double Network
DNase I	Deoxyribonuclease I
DPBA	3-(Acrylamido)phenylboronic Acid
DPPH	2,2-Diphenyl-1-picrylhydrazyl
DTP	3,3′-Dithiobis-(propionohydrazide)
DTT	Dithiothreitol
EG	Ethylene Glycol
EGCG	Epigallocatechin Gallate
EIS	Electrochemical Impedance Spectroscopy
EMI	Electromagnetic interference
Fc	Ferrocene
FTIR	Fourier Transform Infrared Spectroscopy
F127DA	F127 Diacrylate
Gel	Gelatin
GelMA	Methacrylated Gelatin
GelMA-DA	Dopamine-grafted Gelatin Methacrylamide
GF	Gauge Factor
GG	Galactomannan
GSH	Glutathione
HA-ADH	Adipic Acid Dihydrazide-modified Ayaluronic Acid
HABI	Hexaarylbiimidazole
HA-PBA	Phenylborate-modified Hyaluronic Acid
hBM-MSCs	Human Bone Marrow Mesenchymal Stem Cells
HEA	2-Hydroxyethyl Acrylate
HEAA	N-2-hydroxyethyl acrylamide
ILs	1-Vinyl-3-butylimidazole Bromide Salt
IPN	Interpenetrating Polymer Network
LACB	Lipoic Acid-carboxybetaine
LPS	Lipopolysaccharide
LVER	Linear Viscoelastic Region
MRSA	Methicillin-Resistant Staphylococcus aureus
NMR	Nuclear Magnetic Resonance
ODex	Oxidized Dextran
OHA	Oxidized Hyaluronic Acid
OSA	Oxidized Sodium Alginate
PAA-TA	Thioctate-doped Poly(acrylic acid)
PAM	Polyacrylamide
PAMAM	Polyamide Amine
PBS	Phosphate Buffer Solution
pCBMA	Poly(carboxybetaine methacrylate)
PDGF	Platelet-derived Growth Factor
PDI	Polydispersity index
PE	Polyethylene
PEG	Polyethylene Glycol
PEGSD	Poly(glycerol sebacate)-co-poly(ethylene glycol)-g-catechol
PHA-I	Poly(HEA-co-AAPBA-co-ILs)
pHEMA	Poly(hydroxyethyl methacrylate)
PL-Cat	Catechol-modified ε-Poly-l-lysine
PNIPAM	Poly(N-isopropyl acrylamide)
PTFE	Polytetrafluoroethylene
PVA	Polyvinyl Alcohol
PVP	Polyvinyl Pyrrolidone
RGD	Arginine-Glycine-Aspartic Acid
ROS	Reactive Oxygen Species
RT	Room Temperature
SA	Sodium Alginate
SAIP	Self-Aggregation-Induced Polymerization
SEM	Scanning Electron Microscopy
SFMA	Glycidyl-methacrylate-modified Silk Fibroin
SH-WPU	Self-healing Waterborne Polyurethane
SP	Sodium Periodate
TEG	Triethylene Glycol
TGA	Thermogravimetric Analysis
TLA	Three-Letter Acronym
UPy	Ureidopyrimidinone
VEGF	Vascular Endothelial Growth Factor
WPBTS	Wood-based PVA-borate-TA-sodium Sulfate
XPS	X-ray Photoelectron Spectroscopy
XRD	X-ray Diffraction

## References

[1] Dong S., An S., Saiding Q., Chen Q., Liu B., Kong N., Chen W., Tao W. (2025). Therapeutic Hydrogels: Properties and Biomedical Applications. Chem. Rev..

[2] Liu Q., Dong X., Qi H., Zhang H., Li T., Zhao Y., Li G., Zhai W. (2024). 3D Printable Strong and Tough Composite Organo-Hydrogels Inspired by Natural Hierarchical Composite Design Principles. Nat. Commun..

[3] Li J., Mooney D.J. (2016). Designing Hydrogels for Controlled Drug Delivery. Nat. Rev. Mater..

[4] Zhang B., Chen Y., Hou S., Liu Z., Yang Y., Li T., Zhao L., Zhang H., Wei H., Meng L. (2026). Smart Microenvironment-Adaptive Nanocatalytic Hydrogel for Sequential Antibacterial, Anti-Inflammatory, and Regenerative Therapy of Biofilm-Infected Wounds. Bioact. Mater..

[5] Zheng B., Zhou H., Zhao G., Wang K., Wu P., Liu H., Wang P., Yao Y., Xu F. (2025). Bioinspired Electrically Conductive Hydrogels: Rational Engineering for Next-Generation Flexible Mechanosensors. Mater. Sci. Eng. R Rep..

[6] Ma Y., Weder C., Du Prez F.E., Berrocal J.A. (2025). From Beta-Dicarbonyl Chemistry to Dynamic Polymers. Chem. Rev..

[7] Malik U.S., Niazi M.B.K., Jahan Z., Zafar M.I., Vo D.-V.N., Sher F. (2021). Nano-Structured Dynamic Schiff Base Cues as Robust Self-Healing Polymers for Biomedical and Tissue Engineering Applications: A Review. Environ. Chem. Lett..

[8] Cromwell O.R., Chung J., Guan Z. (2015). Malleable and Self-Healing Covalent Polymer Networks through Tunable Dynamic Boronic Ester Bonds. J. Am. Chem. Soc..

[9] Xue X., Li C., Yu X., Chenchai K., Zhang X., Zhang X., Zhang G., Zhang D. (2025). Photo-Patternable and Healable Polymer Semiconductor Enabled by Dynamic Covalent Disulfide Bonding. Angew. Chem. Int. Ed..

[10] Zhang Z., Le G.N.T., Ge Y., Tang X., Chen X., Ejim L., Bordeleau E., Wright G.D., Burns D.C., Tran S. (2023). Isomerization of Bioactive Acylhydrazones Triggered by Light or Thiols. Nat. Chem..

[11] Ratwani C.R., Kamali A.R., Abdelkader A.M. (2023). Self-Healing by Diels-Alder Cycloaddition in Advanced Functional Polymers: A Review. Prog. Mater. Sci..

[12] Li X., Zhang Y., Shi Z., Wang D., Yang H., Zhang Y., Qin H., Lu W., Chen J., Li Y. (2024). Water-Stable Boroxine Structure with Dynamic Covalent Bonds. Nat. Commun..

[13] Zhao H., Li R., Li H., Jin L., Lan Y., Jiang S., Liu M. (2026). Reprogrammable Thermochromic Fiber Actuators for Spatial Temperature Indication Devices. Adv. Funct. Mater..

[14] Yu C., Li X., Yang X., Qiu X., Zhang X., Chen Z., Luo Y. (2024). Dynamic Covalent Bonded Gradient Structured Actuators with Mechanical Robustness and Self-Healing Ability. Small.

[15] Wu J., Zeng F., Fan Z., Xuan S., Hua Z., Liu G. (2024). Hierarchical Hydrogen Bonds Endow Supramolecular Polymers with High Strength, Toughness, and Self-Healing Properties. Adv. Funct. Mater..

[16] Zhou H.-X., Pang X. (2018). Electrostatic Interactions in Protein Structure, Folding, Binding, and Condensation. Chem. Rev..

[17] Miyamae K., Nakahata M., Takashima Y., Harada A. (2015). Self-Healing, Expansion–Contraction, and Shape-Memory Properties of a Preorganized Supramolecular Hydrogel through Host–Guest Interactions. Angew. Chem. Int. Ed..

[18] Lewis J.E.M., Beer P.D., Loeb S.J., Goldup S.M. (2017). Metal Ions in the Synthesis of Interlocked Molecules and Materials. Chem. Soc. Rev..

[19] Wang S., Jiang L. (2007). Definition of Superhydrophobic States. Adv. Mater..

[20] Silverstein T.P. (1998). The Real Reason Why Oil and Water Don’t Mix. J. Chem. Educ..

[21] Zhuang W.R., Wang Y., Cui P.F., Xing L., Lee J., Kim D., Jiang H.L., Oh Y.K. (2019). Applications of Pi-Pi Stacking Interactions in the Design of Drug-Delivery Systems. J. Control. Release.

[22] Appel E.A., del Barrio J., Loh X.J., Scherman O.A. (2012). Supramolecular Polymeric Hydrogels. Chem. Soc. Rev..

[23] Goor O., Hendrikse S.I.S., Dankers P.Y.W., Meijer E.W. (2017). From Supramolecular Polymers to Multi-Component Biomaterials. Chem. Soc. Rev..

[24] Yadav J., Chahal S., Kumar P., Kumar C. (2026). Thermo-Responsive Smart Hydrogels: Molecular Engineering, Dynamic Cross-Linking Strategies, and Therapeutics Applications. Gels.

[25] Peng J., Song W., Zhang W., Li X., Ma Q., Wu B., Fujishige M., Takeuchi K., Endo M., Ji C. (2025). Bone-Inspired Sustainable Hydrogel Electrolytes for Zn Metal Batteries. Angew. Chem. Int. Ed..

[26] Zhang Z., Xian Y., Zhou D., Liu Y., Liu C., Lu W.W., Liu H., Wu D. (2025). Spider Silk Inspired Hierarchical Fibrous Hydrogels with Extreme Robustness Via a Top-down Multidimensional Engineering Strategy. Adv. Funct. Mater..

[27] Han L., Liu K., Wang M., Wang K., Fang L., Chen H., Zhou J., Lu X. (2018). Mussel-Inspired Adhesive and Conductive Hydrogel with Long-Lasting Moisture and Extreme Temperature Tolerance. Adv. Funct. Mater..

[28] Xie C., Wang X., He H., Ding Y., Lu X. (2020). Mussel-Inspired Hydrogels for Self-Adhesive Bioelectronics. Adv. Funct. Mater..

[29] Wang C., Gao X., Zhang F., Hu W., Gao Z., Zhang Y., Ding M., Liang Q. (2022). Mussel Inspired Trigger-Detachable Adhesive Hydrogel. Small.

[30] Choi S., Moon J.R., Park N., Im J., Kim Y.E., Kim J.-H., Kim J. (2023). Bone-Adhesive Anisotropic Tough Hydrogel Mimicking Tendon Enthesis. Adv. Mater..

[31] Wu T., Cui C., Fan C., Xu Z., Liu Y., Liu W. (2021). Tea Eggs-Inspired High-Strength Natural Polymer Hydrogels. Bioact. Mater..

[32] Guo R., Su Q., Zhang J., Dong A., Lin C., Zhang J. (2017). Facile Access to Multisensitive and Self-Healing Hydrogels with Reversible and Dynamic Boronic Ester and Disulfide Linkages. Biomacromolecules.

[33] Chen J., Su Q., Guo R., Zhang J., Dong A., Lin C., Zhang J. (2017). A Multitasking Hydrogel Based on Double Dynamic Network with Quadruple-Stimuli Sensitiveness, Autonomic Self-Healing Property, and Biomimetic Adhesion Ability. Macromol. Chem. Phys..

[34] Ma M., Yang J., Ye Z., Dong A., Zhang J., Zhang J. (2021). A Facile Strategy for Synergistic Integration of Dynamic Covalent Bonds and Hydrogen Bonds to Surmount the Tradeoff between Mechanical Property and Self-Healing Capacity of Hydrogels. Macromol. Mater. Eng..

[35] Li Q., Wang S., Wang Z., Tong W. (2026). Hydrogen Bond Rearrangement of Polyvinyl Alcohol Hydrogel Achieving High Triboelectric Generation. Nano Energy.

[36] Cao H., Xiang D., Zhou X., Yue P., Zou Y., Zhong Z., Ma Y., Wang L., Wu S., Ye Q. (2023). High-Strength, Antibacterial, Antioxidant, Hemostatic, and Biocompatible Chitin/Pegde-Tannic Acid Hydrogels for Wound Healing. Carbohydr. Polym..

[37] Gao M., Hao X., Li Y., Tang Y., Yang W., Zhang K., Lu Y., Zhou X. (2025). Engineering Supramolecular Composite Hydrogels Via “Ph-Induced Ureidopyimidinone (Upy) Tautomerization” Strategy. Adv. Funct. Mater..

[38] Wu Q., Ghosal K., Kana’an N., Roy S., Rashed N., Majumder R., Mandal M., Gao L., Farah S. (2025). On-Demand Imidazolidinyl Urea-Based Tissue-Like, Self-Healable, and Antibacterial Hydrogels for Infectious Wound Care. Bioact. Mater..

[39] Liu T., Wang F., Wu Q., Chen T., Sun P. (2021). Fluorescent, Electrically Responsive and Ultratough Self-Healing Hydrogels Via Bioinspired All-in-One Hierarchical Micelles. Mater. Horiz..

[40] Huang S., Hou L., Li T., Jiao Y., Wu P. (2022). Antifreezing Hydrogel Electrolyte with Ternary Hydrogen Bonding for High-Performance Zinc-Ion Batteries. Adv. Mater..

[41] Chen H., Yuan X., Du M., Wu Q., Kong W., Zhu Z., Wu M., He Y. (2026). Tough Hydrogels with Robust Wet Adhesion Via Entropy-Driven Hydrogen Bond Reorganization. Adv. Mater..

[42] Wang R., Xu T., Yang Y., Zhang M., Xie R., Cheng Y., Zhang Y. (2025). Tough Polyurethane Hydrogels with a Multiple Hydrogen-Bond Interlocked Bicontinuous Phase Structure Prepared by in Situ Water-Induced Microphase Separation. Adv. Mater..

[43] Chen J., Wang D., Wang L.-H., Liu W., Chiu A., Shariati K., Liu Q., Wang X., Zhong Z., Webb J. (2020). An Adhesive Hydrogel with “Load-Sharing” Effect as Tissue Bandages for Drug and Cell Delivery. Adv. Mater..

[44] Tang L., Liu W., Liu G. (2010). High-Strength Hydrogels with Integrated Functions of H-Bonding and Thermoresponsive Surface-Mediated Reverse Transfection and Cell Detachment. Adv. Mater..

[45] Hu X., Vatankhah-Varnoosfaderani M., Zhou J., Li Q., Sheiko S.S. (2015). Weak Hydrogen Bonding Enables Hard, Strong, Tough, and Elastic Hydrogels. Adv. Mater..

[46] Zhao J., Dai H., Qiao K., Wang T., Zhao F., Wang J., Li J., Bahatibieke A., Xie Y., Liu Y. (2026). Viscoelastic Polyurethane Discs with Host-Guest Interaction for Temporomandibular Joint Function Restoration. Adv. Funct. Mater..

[47] Han S., Zhao J., Naskar S., Wu D., Herrmann C., Xia J., Li H. (2026). Host–Guest Interactions Enhance Charge Transport across Single Cyclodextrin/Azobenzene Complex Junction. J. Am. Chem. Soc..

[48] Wu T., Yin J., Zhu S., Hai H., Xie Y., Lu C. (2026). From Cucurbit[7]Uril Armor-Equipped Ferrocene to Nitrogen Self-Doped Porous Carbon Hosting Fe Single Atoms and Atomic Clusters for Orr and Zinc-Air Batteries. Angew. Chem. Int. Ed..

[49] Wang Y., Zhang X., Dai Y., Xia F. (2023). A Photo-Modulated Organometallic Nanozyme Based on Β-Cyclodextrin-Capped Gold Nanoparticles for the Detection of Zn(Ii) and Cascade Catalysis. Chem. Eng. J..

[50] Yu M., Ye Z., Liu S., Zhu Y., Niu X., Wang J., Ao R., Huang H., Cai H., Liu Y. (2024). Redox-Active Ferrocene Quencher-Based Supramolecular Nanomedicine for Nir-Ii Fluorescence-Monitored Chemodynamic Therapy. Angew. Chem. Int. Ed..

[51] Bernhard S., Ritter L., Müller M., Guo W., Guzzi E.A., Bovone G., Tibbitt M.W. (2024). Modular and Photoreversible Polymer–Nanoparticle Hydrogels Via Host–Guest Interactions. Small.

[52] Ren Z., Ke T., Ling Q., Zhao L., Gu H. (2021). Rapid Self-Healing and Self-Adhesive Chitosan-Based Hydrogels by Host-Guest Interaction and Dynamic Covalent Bond as Flexible Sensor. Carbohydr. Polym..

[53] Iudin D., van Steenbergen M.J., Masereeuw R., van Ravensteijn B.G.P., Vermonden T. (2025). Shrinkable Hydrogels through Host–Guest Interactions: A Robust Approach to Obtain Tubular Cell-Laden Scaffolds with Small Diameters. Adv. Funct. Mater..

[54] Pourbadiei B., Adlsadabad S.Y., Rahbariasr N., Pourjavadi A. (2023). Synthesis and Characterization of Dual Light/Temperature-Responsive Supramolecular Injectable Hydrogel Based on Host-Guest Interaction between Azobenzene and Starch-Grafted Β-Cyclodextrin: Melanoma Therapy with Paclitaxel. Carbohydr. Polym..

[55] Sima H., Liu B., Shi X., Zhang C. (2025). Cnt@Cnf/Mxene Hydrogel with Complete Conductive Network for Flexible Anti-Freeze Sensor and Electromagnetic Shielding. Carbohydr. Polym..

[56] Ma A., Wang G., Yang Z., Bai L., Chen H., Wang W., Yang H., Wei D., Yang L. (2020). Fabrication of Janus Graphene Oxide Hybrid Nanosheets by Pickering Emulsion Template for Self-Healing Nanocomposite Hydrogels. Chem. Eng. J..

[57] Shuai F., Zhang Y., Yin Y., Zhao H., Han X. (2021). Fabrication of an Injectable Iron (Iii) Crosslinked Alginate-Hyaluronic Acid Hydrogel with Shear-Thinning and Antimicrobial Activities. Carbohydr. Polym..

[58] Ren A., Jia L., Wang P., Xiang T., Zhou S. (2024). Toughening of Anti-Freezing Ionic Hydrogels with Zr4+-Dicarboxylic Acid Coordination Complex for Low Temperature Sensing Applications. Chem. Eng. J..

[59] Zhou X., Liu Y., Liu L., Wang M., Shi Y., Yu H., Miao Y., Yue B., Zhu L. (2026). Metal-Coordinated Sacrificial-Bond Dual-Network Toughness Architecture: An Ultra-Stretchable and Highly Sensitive Wearable Hydrogel Keyboard for Gesture Recognition. Chem. Eng. J..

[60] Li H., Zhang H., Liu X., Jie J., Yin M., Du J. (2025). Chinese Nian Gao Inspired Textured Janus Hydrogel for Body Signal Sensing and Human Machine Interaction. Adv. Sci..

[61] Xu X., Jerca F.A., Jerca V.V., Hoogenboom R. (2019). Covalent Poly(2-Isopropenyl-2-Oxazoline) Hydrogels with Ultrahigh Mechanical Strength and Toughness through Secondary Terpyridine Metal-Coordination Crosslinks. Adv. Funct. Mater..

[62] Hong L., Chen G., Cai Z., Liu H., Zhang C., Wang F., Xiao Z., Zhong J., Wang L., Wang Z. (2022). Balancing Microthrombosis and Inflammation Via Injectable Protein Hydrogel for Inflammatory Bowel Disease. Adv. Sci..

[63] Pandit S., Ghosh A., Ghanta S., Ghosh P., Mandal M., Roy A., Das R.K. (2025). Mechanically Robust, Stimuli-Responsive, Multi-Functional Luminescent Hydrogels through Strategic Exfoliation of Nano-Clay and Dynamic Lanthanide Ion Complexation. Small.

[64] Li Y., Jiang F., Li X., Wu Y., Wang Y., Peng H., Peng J., Li J., Zhai M. (2025). An Ultrastretchable and Multifunctional Hydrophobic/Electrostatic Dual-Crosslinked Hydrogel for Self-Healing Flexible Touch Panel and Sensor. npj Flex. Electron..

[65] Yan S., Chen Y., Li D., Zheng Y., Fu X., Yu B., Chen S., Ni C., Qi H., Zhou W. (2024). Mechanically Robust, Transparent, Conductive Hydrogels Based on Hydrogen Bonding, Ionic Coordination Interactions and Electrostatic Interactions for Light-Curing 3D Printing. Chem. Eng. J..

[66] Yang L., Li S., Zhao Z., Wang J., Yang X., Lv H. (2024). A Stiff and Tough Triple-Shape Memory Hydrogel Triggered at Body Temperature by Hydrophobic Association and Electrostatic Interaction. Chem. Eng. J..

[67] Lei T., Duan X., Zhao H., Ma S., Ma X., Wang N., Zhang Q., Wan A., Xia Z., Shou W. (2025). A Multifunctional Flexible Wearable Hydrogel Sensor with Anti-Swelling Via Supramolecular Interactions for Underwater Motion Detection and Information Transmission. Chem. Eng. J..

[68] Zhang T., Cheng Q., Ye D., Chang C. (2017). Tunicate Cellulose Nanocrystals Reinforced Nanocomposite Hydrogels Comprised by Hybrid Cross-Linked Networks. Carbohydr. Polym..

[69] Zhang H., Chen L., Xiao Y., Guo X., Zhao S., Guan R., Long T., Cheng X., Zhou C. (2025). Development of Strong and Tough Silicone Hydrogels Enabled by Polymerization-Induced Microphase Separation and Multiscaled Electrostatic Interactions and Their Application as Water-Shrinkable Sleeves. Adv. Funct. Mater..

[70] Xiang M., Xiao A., Rodrigue D., Chen X.Y., Wu Y., Liu Y., Ma F., Kong J., Wang Y. (2025). Controlled Hydrogel Surfaces Adhesion Via Macrophase Separation Polymerization Triggered by Electrostatic Interaction for Wound Dressing and Bio-Sensor. Adv. Funct. Mater..

[71] Yang X., Qie H., Ma S., Yang X., Li S., Li N., Liu P., Zhang C., Lü S. (2026). Beyond Swelling and Shrinking: Achieving a Quasi-Isovolumetric Phase Transition in Water-Driven Thermo-Responsive Hydrogels Via Enthalpy–Entropy Compensation. Angew. Chem. Int. Ed..

[72] Yang L., Ren H., Lei P., Long Y., Chen Z., Ge X., Yao J. (2026). Hydrophobic Association Enabled Salt-Concentrated All-Cellulose Hydrogel Electrolytes for High-Temperature Zinc-Ion Hybrid Supercapacitors. Angew. Chem. Int. Ed..

[73] Li K., Jia J., Lu J., Hai N., Li Y., Yan Y., Zhang J. (2026). Tardigrade-Inspired Wide-Temperature-Adaptable and Shape-Programmable Hydrogel Dampers. Adv. Funct. Mater..

[74] Basu A., Kunduru K.R., Doppalapudi S., Domb A.J., Khan W. (2016). Poly(Lactic Acid) Based Hydrogels. Adv. Drug Deliv. Rev..

[75] Webber M.J., Pashuck E.T. (2021). (Macro)Molecular Self-Assembly for Hydrogel Drug Delivery. Adv. Drug Deliv. Rev..

[76] Hoang H.T., Vu T.T., Karthika V., Jo S.H., Jo Y.J., Seo J.W., Oh C.W., Park S.H., Lim K.T. (2022). Dual Cross-Linked Chitosan/Alginate Hydrogels Prepared by Nb-Tz ‘Click’ Reaction for Ph Responsive Drug Delivery. Carbohydr. Polym..

[77] Kono H., Otaka F., Ozaki M. (2014). Preparation and Characterization of Guar Gum Hydrogels as Carrier Materials for Controlled Protein Drug Delivery. Carbohydr. Polym..

[78] Hou Y., Li Y., Li Y., Li D., Guo T., Deng X., Zhang H., Xie C., Lu X. (2023). Tuning Water-Resistant Networks in Mussel-Inspired Hydrogels for Robust Wet Tissue and Bioelectronic Adhesion. ACS Nano.

[79] Fan X., Fang Y., Zhou W., Yan L., Xu Y., Zhu H., Liu H. (2021). Mussel Foot Protein Inspired Tough Tissue-Selective Underwater Adhesive Hydrogel. Mater. Horiz..

[80] Chen R., Xin M., Yi Z., Yan X., Chen T. (2026). Cohesion-Adhesion Balance Hydrogel by Confining Polymer Chain Mobility Via Hydrophobic Chains. Chem. Eng. J..

[81] Gao Y., Wang Y., Liao Y., Chang D., Peng L., Li R., Zhao X., Li K., Li H., Cao M. (2026). π–π Stacking Origin of Irreversible Dispersibility of Graphene Oxide. Nat. Commun..

[82] Deng S., He S., Yan G., Wang L., Li Z., Chen J., Lai J., Li D., Xiang D., Zhao C. (2025). π–π Conjugated Bonds Stacking/Scattering for Switchable Lubrication in Supramolecular Hydrogel. Adv. Sci..

[83] Song J., Zeng J., Chen X., Wang J., Zhang Y., Gao Y., Wang R., Jiang N., Lin Y., Li R. (2025). Anti-Neuroinflammatory Agent Rhein Lysinate-Based Self-Assembled Injectable Hydrogel Loaded with Zl006 for Promoting Post-Stroke Functional Recovery. Biomaterials.

[84] Du H., Liu J., Pan B., Yang H.-Y., Liu G.-B., Lu K. (2022). Fabrication of the Low Molecular Weight Peptide-Based Hydrogels and Analysis of Gelation Behaviors. Food Hydrocoll..

[85] Liu Q., Chiu A., Wang L., An D., Li W., Chen E.Y., Zhang Y., Pardo Y., McDonough S.P., Liu L. (2020). Developing Mechanically Robust, Triazole-Zwitterionic Hydrogels to Mitigate Foreign Body Response (Fbr) for Islet Encapsulation. Biomaterials.

[86] Wang Z., Li T., Huang X., Xu R., Zhao Y., Wei J., Pi W., Yao S., Lu J., Zhang X. (2025). Chiral Helix Amplification and Enhanced Bioadhesion of Two-Component Low Molecular Weight Hydrogels Regulated by Oh to Eradicate Mrsa Biofilms. Mater. Horiz..

[87] Li Y., Yang D., Wu Z., Gao F.-L., Gao X.-Z., Zhao H.-Y., Li X., Yu Z.-Z. (2023). Self-Adhesive, Self-Healing, Biocompatible and Conductive Polyacrylamide Nanocomposite Hydrogels for Reliable Strain and Pressure Sensors. Nano Energy.

[88] Chen P., He S., Zou Z., Wang T., Hu J., Tao J., Yang L., Li Y. (2025). Intelligent Hydrogels Enabled Large-Scale Variability in Continuously Tunable Microwave Absorption. Adv. Funct. Mater..

[89] Sang C., Wang S., Jin X., Cheng X., Xiao H., Yue Y., Han J. (2024). Nanocellulose-Mediated Conductive Hydrogels with Nir Photoresponse and Fatigue Resistance for Multifunctional Wearable Sensors. Carbohydr. Polym..

[90] Liu T., Xie Z., Chen M., Tang S., Liu Y., Wang J., Zhang R., Wang H., Guo X., Gu A. (2022). Mussel-Inspired Dual-Crosslinked Polyamidoxime Photothermal Hydrogel with Enhanced Mechanical Strength for Highly Efficient and Selective Uranium Extraction from Seawater. Chem. Eng. J..

[91] Luo J., Shi X., Li L., Tan Z., Feng F., Li J., Pang M., Wang X., He L. (2021). An Injectable and Self-Healing Hydrogel with Controlled Release of Curcumin to Repair Spinal Cord Injury. Bioact. Mater..

[92] Jing X., Mi H.-Y., Napiwocki B.N., Peng X.-F., Turng L.-S. (2017). Mussel-Inspired Electroactive Chitosan/Graphene Oxide Composite Hydrogel with Rapid Self-Healing and Recovery Behavior for Tissue Engineering. Carbon.

[93] Han H., Wang Z., Wu T., Zhao F., Huang S., Peng B., Zhao C., Han Q., Liu E., Liu Q. (2026). Dual-Crosslinked Injectable Polysaccharide-Based Hydrogel with Synergistic Photothermal-Controlled Release of Black Phosphorus-Baicalein for Accelerating Wound Healing. Carbohydr. Polym..

[94] Huan Y., Kong Q., Tang Q., Wang Y., Mou H., Ying R., Li C. (2022). Antimicrobial Peptides/Ciprofloxacin-Loaded O-Carboxymethyl Chitosan/Self-Assembling Peptides Hydrogel Dressing with Sustained-Release Effect for Enhanced Anti-Bacterial Infection and Wound Healing. Carbohydr. Polym..

[95] Zhou T., Ran J., Xu P., Shen L., He Y., Ye J., Wu L., Gao C. (2022). A Hyaluronic Acid/Platelet-Rich Plasma Hydrogel Containing Mno(2) Nanozymes Efficiently Alleviates Osteoarthritis in Vivo. Carbohydr. Polym..

[96] Tohamy H.-A.S. (2024). Cellulosic Schiff Base Hydrogel Biosensor for Bacterial Detection with Ph/Thermo-Responsitivity: Dft Calculations and Molecular Docking. Int. J. Biol. Macromol..

[97] Shi H., Ma D., Wu D., Qiu X., Yang S., Wang Y., Xiao L., Ji X., Zhang W., Han S. (2024). A Ph-Responsive, Injectable and Self-Healing Chitosan-Coumarin Hydrogel Based on Schiff Base and Hydrogen Bonds. Int. J. Biol. Macromol..

[98] Li S., Pei M., Wan T., Yang H., Gu S., Tao Y., Liu X., Zhou Y., Xu W., Xiao P. (2020). Self-Healing Hyaluronic Acid Hydrogels Based on Dynamic Schiff Base Linkages as Biomaterials. Carbohydr. Polym..

[99] Li J., Du C., Yang X., Yao Y., Qin D., Meng F., Yang S., Tan Y., Chen X., Jiang W. (2025). Instantaneous Self-Healing Chitosan Hydrogels with Enhanced Drug Leakage Resistance for Infected Stretchable Wounds Healing. Small.

[100] Zhao G., Gu T., Chen L., Wang L., Petrov Y.V., Baulin V.E., Tsivadze A.Y., Jia D., Zhou Y., Li B. (2025). Synergistic Adhesion Enhancement of Double-Crosslinking Chitosan Hydrogel Via Catechol-Fe(3+) Coordination and Schiff Base. Carbohydr. Polym..

[101] Su H., Zheng R., Jiang L., Zeng N., Yu K., Zhi Y., Shan S. (2021). Dextran Hydrogels Via Disulfide-Containing Schiff Base Formation: Synthesis, Stimuli-Sensitive Degradation and Release Behaviors. Carbohydr. Polym..

[102] Mai X., Yang Y., Wang D., Xu W., Sun Z., Liu F. (2025). Novel Dual-Crosslinked Antimicrobial Hydrogels of Oxidized Hyaluronic Acid/Gelatin Grafted with Curcumin-Zn2+ Complexes for Preservation of Chilled Chicken. Food Packag. Shelf Life.

[103] Gu Z., Wang J., Shi X., Zhou Y., Chen Y., He Y., Wu L., Cui Y., Gu Z. (2026). Dynamic Boronate Ester Crosslinked Hyaluronic Acid Hydrogel Patch Enabling Fast Dissolution and Rapid Oral Mucosal Delivery of Amorphous Sumatriptan. Carbohydr. Polym..

[104] Marozas I.A., Anseth K.S., Cooper-White J.J. (2019). Adaptable Boronate Ester Hydrogels with Tunable Viscoelastic Spectra to Probe Timescale Dependent Mechanotransduction. Biomaterials.

[105] Neumann M., Kozinec D., Hene D., van Uden L., Schneeberger K., van der Laan L.J.W., Drost J., Slaats G.G., Verhaar M.C., Vermonden T. (2026). Dynamic Matrix Remodeling in Boronate Ester Hydrogels for 3d Organoid Cultures. J. Control. Release.

[106] Zhou Y., Liang X., Shen Z., Zhang R., Zhang G., Yu B., Li Y., Xu F.J. (2026). Glucose-Responsive Hydrogel with Adaptive Insulin Release to Modulate Hyperglycemic Microenvironment and Promote Wound Healing. Biomaterials.

[107] Wang Y., Yu H., Wang L., Zhang L., Hu J., Feng J., Deng Z., Chen C. (2026). A Two-Birds-with-One-Stone Polysaccharide-Based Microneedle Therapy: Prolonged Glycemic Regulation and Accelerated Diabetic Wound Healing. Carbohydr. Polym..

[108] Ali A., Govindharaj M., Fatma B., Alshehhi K.H., Islayem D., Alsaafeen N.B., Pappa A.M., Pitsalidis C. (2025). In Situ Development of Self-Healing, Injectable, Glucose and Ph-Responsive Electroconductive Composite Hydrogels. Adv. Compos. Hybrid Mater..

[109] Zhu S., Dai Q., Yao L., Wang Z., He Z., Li M., Wang H., Li Q., Gao H., Cao X. (2022). Engineered Multifunctional Nanocomposite Hydrogel Dressing to Promote Vascularization and Anti-Inflammation by Sustained Releasing of Mg2+ for Diabetic Wounds. Compos. Part B Eng..

[110] Fass D., Thorpe C. (2018). Chemistry and Enzymology of Disulfide Cross-Linking in Proteins. Chem. Rev..

[111] Wu Y., Chang X., Yang G., Chen L., Wu Q., Gao J., Tian R., Mu W., Gooding J.J., Chen X. (2023). A Physiologically Responsive Nanocomposite Hydrogel for Treatment of Head and Neck Squamous Cell Carcinoma Via Proteolysis-Targeting Chimeras Enhanced Immunotherapy. Adv. Mater..

[112] Xu K., Yao H., Fan D., Zhou L., Wei S. (2021). Hyaluronic Acid Thiol Modified Injectable Hydrogel: Synthesis, Characterization, Drug Release, Cellular Drug Uptake and Anticancer Activity. Carbohydr. Polym..

[113] Guo T., Wang W., Song J., Jin Y., Xiao H. (2021). Dual-Responsive Carboxymethyl Cellulose/Dopamine/Cystamine Hydrogels Driven by Dynamic Metal-Ligand and Redox Linkages for Controllable Release of Agrochemical. Carbohydr. Polym..

[114] Liu Y., Zhang F., Ru Y. (2015). Hyperbranched Phosphoramidate-Hyaluronan Hybrid: A Reduction-Sensitive Injectable Hydrogel for Controlled Protein Release. Carbohydr. Polym..

[115] Feng S., Niu L., Wang X., Zhang Q., You R., Li M., Feng Y. (2025). Injectable Self-Crosslinking Hyaluronic Acid/Silk Fibroin Blend Hydrogel Based on Disulfide Bond. Carbohydr. Polym..

[116] Yang X., Yang H., Jiang X., Yang B., Zhu K., Lai N.C., Huang C., Chang C., Bian L., Zhang L. (2021). Injectable Chitin Hydrogels with Self-Healing Property and Biodegradability as Stem Cell Carriers. Carbohydr. Polym..

[117] Shen J., Chang R., Chang L., Wang Y., Deng K., Wang D., Qin J. (2022). Light Emitting Cmc-Cho Based Self-Healing Hydrogel with Injectability for in Vivo Wound Repairing Applications. Carbohydr. Polym..

[118] Xu Y., Lu G., Chen M., Wang P., Li Z., Han X., Liang J., Sun Y., Fan Y., Zhang X. (2020). Redox and Ph Dual-Responsive Injectable Hyaluronan Hydrogels with Shape-Recovery and Self-Healing Properties for Protein and Cell Delivery. Carbohydr. Polym..

[119] Zhao J., Hu Y., Liu C., Yan C., Liu Z., Chen Z., Nie Z., Xu X., Cui M., Zhao P. (2026). Tissue-Conforming Organoselenium Hydrogel with Microphase-Controlled Acylhydrazone Crosslinking Kinetics Expedites Diabetic Wound Healing by Inhibiting Age-Rage Pathways. Adv. Mater..

[120] Ilochonwu B.C., van der Lugt S.A., Annala A., Di Marco G., Sampon T., Siepmann J., Siepmann F., Hennink W.E., Vermonden T. (2023). Thermo-Responsive Diels-Alder Stabilized Hydrogels for Ocular Drug Delivery of a Corticosteroid and an Anti-Vegf Fab Fragment. J. Control. Release.

[121] Li D.Q., Wang S.Y., Meng Y.J., Guo Z.W., Cheng M.M., Li J. (2021). Fabrication of Self-Healing Pectin/Chitosan Hybrid Hydrogel Via Diels-Alder Reactions for Drug Delivery with High Swelling Property, Ph-Responsiveness, and Cytocompatibility. Carbohydr. Polym..

[122] Bi B., Ma M., Lv S., Zhuo R., Jiang X. (2019). In-Situ Forming Thermosensitive Hydroxypropyl Chitin-Based Hydrogel Crosslinked by Diels-Alder Reaction for Three Dimensional Cell Culture. Carbohydr. Polym..

[123] Yang Z., Zhao F., Zhang W., Yang Z., Luo M., Liu L., Cao X., Chen D., Chen X. (2021). Degradable Photothermal Bioactive Glass Composite Hydrogel for the Sequential Treatment of Tumor-Related Bone Defects: From Anti-Tumor to Repairing Bone Defects. Chem. Eng. J..

[124] Rajawasam C.W.H., Saha N.K., Tran C., Costantino S.M., Weeks M., McCoy K., Sparks J.L., Hartley C.S., Konkolewicz D. (2026). Programmable Hydrogels by Combining Persistent and Transient Dynamic Bonds. Chem. Eng. J..

[125] Mantooth S.M., Munoz-Robles B.G., Webber M.J. (2019). Dynamic Hydrogels from Host–Guest Supramolecular Interactions. Macromol. Biosci..

[126] Li S., Chu X., Hao A., Shang N., Wang C. (2018). A Triply-Responsive Supramolecular Vesicle Fabricated by A-Cyclodextrin Based Host–Guest Recognition and Double Dynamic Covalent Bonds. Soft Matter.

[127] Zou L., Zhang Y., Ning F., Fu X., Webber M.J., Zhang Y. (2025). Dynamic Hydrogels Using Movable Supramolecular Cores to Prepare Multiarm Macromer Analogs. Macromolecules.

[128] Huang X., Wang X., Shi C., Liu Y., Wei Y. (2021). Research on Synthesis and Self-Healing Properties of Interpenetrating Network Hydrogels Based on Reversible Covalent and Reversible Non-Covalent Bonds. J. Polym. Res..

[129] Wang G., Zheng M., Chen H., Liu R., Wu W., Fang X., Shen C., Chen H., Gao H. (2026). A Dual-Dynamic-Bond Network Enabled, Ultra-Stretchable, and Self-Healing Hydrogel for Fresh Produce Damage Prevention in Postharvest Logistics. Chem. Eng. J..

[130] Jia S., Feng Z., Yan X., Zhang Z., Rao J., Shi Z., Ren J., Peng F. (2025). Multifunctional Bio-Based Wearable Ionogel with Hierarchical Dynamic Covalent Crosslinked Double Networks Enabled by Surface Active Xylan. Carbohydr. Polym..

[131] Ren L., Luo Y., Huang Y., Shi X., Lin H., Zhang T., Gong Y., Liu Y., Zheng D., Li W. (2025). Engineering Ros-Responsive Double Network Hydrogel as Bioactive Barrier for Postoperative Abdominal Adhesions Prevention. Bioact. Mater..

[132] Ke Y., Lan K., Wong J.Y., Lu H., Gao S., Ryu K., Chen F., Loh W.W., Dong Z., Lim J.Y.C. (2025). Sustainable DNA-Polysaccharide Hydrogels as Recyclable Bioplastics. Nat. Commun..

[133] Huang Z., Wang M., Chai L., Chen H., Chen D., Li Y., Liu H., Wu Y., Yang X., He L. (2024). Glucose-Responsive, Self-Healing, Wet Adhesive and Multi-Biofunctional Hydrogels for Diabetic Wound Healing. Mater. Today Bio.

[134] Chen B., Liu Z., Shen Z., Gong H., Jiang Y., Su Y., Zhou J., Li Y. (2025). Dual-Property Lignin Macromolecule Featuring Rigidity and Flexibility for the Preparation of Supramolecular Hydrogels with Creep Resistance and Excellent Toughness. Chem. Eng. J..

[135] Guo Z., Gu H., He Y., Zhang Y., Xu W., Zhang J., Liu Y., Xiong L., Chen A., Feng Y. (2020). Dual Dynamic Bonds Enable Biocompatible and Tough Hydrogels with Fast Self-Recoverable, Self-Healable and Injectable Properties. Chem. Eng. J..

[136] González K., García-Astrain C., Santamaria-Echart A., Ugarte L., Avérous L., Eceiza A., Gabilondo N. (2018). Starch/Graphene Hydrogels Via Click Chemistry with Relevant Electrical and Antibacterial Properties. Carbohydr. Polym..

[137] Zhao H., Meng D., Wang Y., Wang Y., Li Q., Guo Y., Song Q., Wang L. (2026). A Multifunctional Hydrogel for Obesity-Associated Tumor Immunotherapy and Postsurgical Wound Healing Promotion. Bioact. Mater..

[138] Xie Z., Chen Z., Lu Q., Chen R., Meng Y., Tian L., Zhu H., He H., Wang L., Wang S. (2025). Benign Separated Cellulose Adhesive Hydrogel Via Constructing Double Dynamic Covalent Bonds for Ultra-Fast Hemostasis and Antibacterial. Chem. Eng. J..

[139] Zhang F., Zhang S., Cui S., Jing X., Feng Y., Coseri S. (2024). Rapid Self-Healing Carboxymethyl Chitosan/Hyaluronic Acid Hydrogels with Injectable Ability for Drug Delivery. Carbohydr. Polym..

[140] Liang R., Zhang H., Wang Y., Ye J., Guo L., He L., Li X., Qiu T., Tuo X. (2022). Dual Dynamic Network System Constructed by Waterborne Polyurethane for Improved and Recoverable Performances. Chem. Eng. J..

[141] Zhang W., Ren M., Chen M., Wu L. (2025). Tough and Self-Healing Waterborne Polyurethane Elastomers Via Hydrogen Bonds and Oxime–Carbamate Design for Wearable Flexible Strain Sensors. RSC Adv..

[142] Fang Y., Xu J., Gao F., Du X., Du Z., Cheng X., Wang H. (2021). Self-Healable and Recyclable Polyurethane-Polyaniline Hydrogel toward Flexible Strain Sensor. Compos. Part B Eng..

[143] Xiang S.-L., Su Y.-X., Yin H., Li C., Zhu M.-Q. (2021). Visible-Light-Driven Isotropic Hydrogels as Anisotropic Underwater Actuators. Nano Energy.

[144] Yu S., Liu C., Lou Z., Qiao W. (2025). Sodium Alginate Silk Fibroin Hydrogel Loaded with Neural Stem Cells for Treatment of Cerebral Palsy. Biomacromolecules.

[145] Liu X., Ren Z., Liu F., Zhao L., Ling Q., Gu H. (2021). Multifunctional Self-Healing Dual Network Hydrogels Constructed Via Host–Guest Interaction and Dynamic Covalent Bond as Wearable Strain Sensors for Monitoring Human and Organ Motions. ACS Appl. Mater. Interfaces.

[146] Du J., Wang X., Song R., Lv H., Zong S., Zhao Q., Wu J., Wen X., Jiang J., Duan J. (2023). Application of Adhesive Controllable Galactomannan Hydrogel Initiated by Aluminum Ions at Room Temperature in Flexible Sensors. React. Funct. Polym..

[147] Li S., Chen N., Li X., Li Y., Xie Z., Ma Z., Zhao J., Hou X., Yuan X. (2020). Bioinspired Double-Dynamic-Bond Crosslinked Bioadhesive Enables Post-Wound Closure Care. Adv. Funct. Mater..

[148] Liao Y., Zhao Y., Yu C., Zhang Z., Yuan Y., Ouyang L., Hu W., Zhang S., Al-Smadi F., Mi B. (2026). Ph-Responsive and Dual-Dynamically Crosslinked Metal-Phenolic Hydrogel for Synergistic Macrophage and Th17/Treg Reprogramming in Diabetic Wounds. J. Nanobiotechnol..

[149] Liang Y., Li Z., Huang Y., Yu R., Guo B. (2021). Dual-Dynamic-Bond Cross-Linked Antibacterial Adhesive Hydrogel Sealants with on-Demand Removability for Post-Wound-Closure and Infected Wound Healing. ACS Nano.

[150] Xu J., Yan S., Qi B., Jiang L. (2025). New Insights into the Cross-Linking Mechanism of Soybean Protein-Based Double Dynamic Cross-Linking Hydrogels for the Controlled Delivery of Curcumin. Food Res. Int..

[151] Zhang Z., Ding Y., Yuan H., Rui C., Fan P., Ji Y., Xiao Y., Dai J., Li L. (2025). A Multiple-Crosslinked Injectable Hydrogel for Modulating Tissue Microenvironment and Accelerating Infected Diabetic Wound Repair. J. Nanobiotechnol..

[152] Duan Y., Li Y., Wang Y., Su L., Li Q., Jiang F., Liu S., Huang Z., Zhou X., Tang H. (2025). An All-in-One “Multifunctional Hydrogel”: Through Antibacterial, Anti-Inflammatory, and Angiogenic to Promoting Mrsa-Infected Bone Defect Repair. Chem. Eng. J..

[153] Fu H., Wang B., Li J., Cao D., Zhang W., Xu J., Li J., Zeng J., Gao W., Chen K. (2024). Ultra-Strong, Nonfreezing, and Flexible Strain Sensors Enabled by Biomass-Based Hydrogels through Triple Dynamic Bond Design. Mater. Horiz..

[154] Yang L., Jing F., Wei D., Zhao X., Tao Y., Liu T., Zhang T. (2025). Assembled Granular Hydrogels Loaded with Growth Factors for Enhanced Mesenchymal Stem Cell Therapy in Abdominal Wall Defect Repair. J. Control. Release.

[155] Qian K., Zhou J., Miao M., Thaiboonrod S., Fang J., Feng X. (2024). Stretchable Supramolecular Hydrogel with Instantaneous Self-Healing for Electromagnetic Interference Shielding Control and Sensing. Compos. Part B Eng..

[156] Li X., Gao J., Gao L., Jin Y., Cai M., Liu J., Zhang Y., Li J., Wu T., Elsabahy M. (2025). Self-Aggregation-Induced Polymerization for Constructing Multifunctional Dynamic Zwitterionic Hydrogels. Aggregate.

[157] Li R.K., Qi Q.B., Wang C.H., Hou G.G., Li C.B. (2023). Self-Healing Hydrogels Fabricated by Introducing Antibacterial Long-Chain Alkyl Quaternary Ammonium Salt into Marine-Derived Polysaccharides for Wound Healing. Polymers.

[158] Zhang Z.J., Bu J.S., Li B.Y., Xuan H.Y., Jin Y., Yuan H.H. (2022). Dynamic Double Cross-Linked Self-Healing Polysaccharide Hydrogel Wound Dressing Based on Schiff Base and Thiol-Alkynone Reactions. Int. J. Mol. Sci..

[159] Wang C., Wei P., Li B., Sun N. (2025). Multifunctional Dynamic Hydrogel Based on a Micelle-Cross-Linked Chitosan Network for Infected Wound Healing. ACS Omega.

[160] Wang Z., Zhang R.X., Zhang C., Dai C., Ju X., He R. (2019). Fabrication of Stable and Self-Assembling Rapeseed Protein Nanogel for Hydrophobic Curcumin Delivery. J. Agric. Food Chem..

[161] Marić I., Yang L., Li X., Santiago G.M., Pappas C.G., Qiu X., Dijksman J.A., Mikhailov K., van Rijn P., Otto S. (2023). Tailorable and Biocompatible Supramolecular-Based Hydrogels Featuring Two Dynamic Covalent Chemistries. Angew. Chem. Int. Ed..

[162] Sousa V., Amaral A.J.R., Castanheira E.J., Marques I., Rodrigues J.M.M., Félix V., Borges J., Mano J.F. (2023). Self-Supporting Hyaluronic Acid-Functionalized G-Quadruplex-Based Perfusable Multicomponent Hydrogels Embedded in Photo-Cross-Linkable Matrices for Bioapplications. Biomacromolecules.

[163] Ma S., Wang Z., Fan H., He H. (2026). Silk Fibroin–Polyphenol Gels and Hydrogels: Molecular Interactions, Gelation Strategies, Responsive Behaviors, and Multifunctional Applications. Gels.

[164] Teng Y., Zhang Z., Cui Y., Su Z., Godwin M., Chung T., Zhou Y., Leontowich A.F.G., Islam M.S., Tam K.C. (2025). High-Sensitivity and Flexible Motion Sensing Enabled by Robust, Self-Healing Wood-Based Anisotropic Hydrogel Composites. Small.

[165] Li J., Chuang R., Liu M., Ma Y., Zhang H., Li H., Xia N., Rayan A.M., Ghamry M. (2025). Kappa-Carrageenan Enhances the Mechanical and Rheological Properties of Egg Yolk Low-Density Lipoprotein Gels with an Interpenetrating Network Structure. Food Hydrocoll..

[166] Qi T., Liu X.F., Zheng N., Huang J., Xiang W.L., Nie Y.J., Guo Z.R., Cai B.X. (2025). Self-Healable, Antimicrobial and Conductive Hydrogels Based on Dynamic Covalent Bonding with Silver Nanoparticles for Flexible Sensor. Polymers.

[167] Qin H., Zhang T., Li H.-N., Cong H.-P., Antonietti M., Yu S.-H. (2017). Dynamic Au-Thiolate Interaction Induced Rapid Self-Healing Nanocomposite Hydrogels with Remarkable Mechanical Behaviors. Chem.

[168] Han J., Cui Y., Han X., Liang C., Liu W., Luo D., Yang D. (2020). Super-Soft DNA/Dopamine-Grafted-Dextran Hydrogel as Dynamic Wire for Electric Circuits Switched by a Microbial Metabolism Process. Adv. Sci..

[169] Yang Q., Miao Y., Luo J., Chen Y., Wang Y. (2023). Amyloid Fibril and Clay Nanosheet Dual-Nanoengineered DNA Dynamic Hydrogel for Vascularized Bone Regeneration. ACS Nano.

